# The impact of the Fungus-Host-Microbiota interplay upon *Candida albicans* infections: current knowledge and new perspectives

**DOI:** 10.1093/femsre/fuaa060

**Published:** 2020-11-24

**Authors:** Christophe d'Enfert, Ann-Kristin Kaune, Leovigildo-Rey Alaban, Sayoni Chakraborty, Nathaniel Cole, Margot Delavy, Daria Kosmala, Benoît Marsaux, Ricardo Fróis-Martins, Moran Morelli, Diletta Rosati, Marisa Valentine, Zixuan Xie, Yoan Emritloll, Peter A Warn, Frédéric Bequet, Marie-Elisabeth Bougnoux, Stephanie Bornes, Mark S Gresnigt, Bernhard Hube, Ilse D Jacobsen, Mélanie Legrand, Salomé Leibundgut-Landmann, Chaysavanh Manichanh, Carol A Munro, Mihai G Netea, Karla Queiroz, Karine Roget, Vincent Thomas, Claudia Thoral, Pieter Van den Abbeele, Alan W Walker, Alistair J P Brown

**Affiliations:** Unité Biologie et Pathogénicité Fongiques, Département de Mycologie, Institut Pasteur, USC 2019 INRA, 25, rue du Docteur Roux, 75015 Paris, France; Aberdeen Fungal Group, Institute of Medical Sciences, University of Aberdeen, Ashgrove Road West, Foresterhill, Aberdeen AB25 2ZD, UK; BIOASTER Microbiology Technology Institute, 40 avenue Tony Garnier, 69007 Lyon, France; Université de Paris, Sorbonne Paris Cité, 25, rue du Docteur Roux, 75015 Paris, France; Microbial Immunology Research Group, Emmy Noether Junior Research Group Adaptive Pathogenicity Strategies, and the Department of Microbial Pathogenicity Mechanisms, Leibniz Institute for Natural Product Research and Infection Biology – Hans Knöll Institute, Beutenbergstraße 11a, 07745 Jena, Germany; Institute of Microbiology, Friedrich Schiller University, Neugasse 25, 07743 Jena, Germany; Gut Microbiology Group, Rowett Institute, University of Aberdeen, Ashgrove Road West, Foresterhill, Aberdeen AB25 2ZD, UK; Unité Biologie et Pathogénicité Fongiques, Département de Mycologie, Institut Pasteur, USC 2019 INRA, 25, rue du Docteur Roux, 75015 Paris, France; Université de Paris, Sorbonne Paris Cité, 25, rue du Docteur Roux, 75015 Paris, France; Unité Biologie et Pathogénicité Fongiques, Département de Mycologie, Institut Pasteur, USC 2019 INRA, 25, rue du Docteur Roux, 75015 Paris, France; Université de Paris, Sorbonne Paris Cité, 25, rue du Docteur Roux, 75015 Paris, France; ProDigest BV, Technologiepark 94, B-9052 Gent, Belgium; Center for Microbial Ecology and Technology (CMET), Department of Biotechnology, Faculty of Bioscience Engineering, Ghent University, Coupure Links, 9000 Ghent, Belgium; Immunology Section, Vetsuisse Faculty, University of Zurich, Winterthurerstrasse 266a, Zurich 8057, Switzerland; Institute of Experimental Immunology, University of Zurich, Winterthurerstrasse 190, Zürich 8057, Switzerland; Mimetas, Biopartner Building 2, J.H. Oortweg 19, 2333 CH Leiden, The Netherlands; Department of Internal Medicine and Radboud Center for Infectious Diseases, Radboud University Medical Center, Geert Grooteplein 28, 6525 GA Nijmegen, The Netherlands; Microbial Immunology Research Group, Emmy Noether Junior Research Group Adaptive Pathogenicity Strategies, and the Department of Microbial Pathogenicity Mechanisms, Leibniz Institute for Natural Product Research and Infection Biology – Hans Knöll Institute, Beutenbergstraße 11a, 07745 Jena, Germany; Gut Microbiome Group, Vall d'Hebron Institut de Recerca (VHIR), Vall d'Hebron Hospital Universitari, Vall d'Hebron Barcelona Hospital Campus, Passeig Vall d'Hebron 119–129, 08035 Barcelona, Spain; Unité Biologie et Pathogénicité Fongiques, Département de Mycologie, Institut Pasteur, USC 2019 INRA, 25, rue du Docteur Roux, 75015 Paris, France; Magic Bullet Consulting, Biddlecombe House, Ugbrook, Chudleigh Devon, TQ130AD, UK; BIOASTER Microbiology Technology Institute, 40 avenue Tony Garnier, 69007 Lyon, France; Unité Biologie et Pathogénicité Fongiques, Département de Mycologie, Institut Pasteur, USC 2019 INRA, 25, rue du Docteur Roux, 75015 Paris, France; Université Clermont Auvergne, INRAE, VetAgro Sup, UMRF0545, 20 Côte de Reyne, 15000 Aurillac, France; Microbial Immunology Research Group, Emmy Noether Junior Research Group Adaptive Pathogenicity Strategies, and the Department of Microbial Pathogenicity Mechanisms, Leibniz Institute for Natural Product Research and Infection Biology – Hans Knöll Institute, Beutenbergstraße 11a, 07745 Jena, Germany; Microbial Immunology Research Group, Emmy Noether Junior Research Group Adaptive Pathogenicity Strategies, and the Department of Microbial Pathogenicity Mechanisms, Leibniz Institute for Natural Product Research and Infection Biology – Hans Knöll Institute, Beutenbergstraße 11a, 07745 Jena, Germany; Microbial Immunology Research Group, Emmy Noether Junior Research Group Adaptive Pathogenicity Strategies, and the Department of Microbial Pathogenicity Mechanisms, Leibniz Institute for Natural Product Research and Infection Biology – Hans Knöll Institute, Beutenbergstraße 11a, 07745 Jena, Germany; Unité Biologie et Pathogénicité Fongiques, Département de Mycologie, Institut Pasteur, USC 2019 INRA, 25, rue du Docteur Roux, 75015 Paris, France; Immunology Section, Vetsuisse Faculty, University of Zurich, Winterthurerstrasse 266a, Zurich 8057, Switzerland; Institute of Experimental Immunology, University of Zurich, Winterthurerstrasse 190, Zürich 8057, Switzerland; Gut Microbiome Group, Vall d'Hebron Institut de Recerca (VHIR), Vall d'Hebron Hospital Universitari, Vall d'Hebron Barcelona Hospital Campus, Passeig Vall d'Hebron 119–129, 08035 Barcelona, Spain; Aberdeen Fungal Group, Institute of Medical Sciences, University of Aberdeen, Ashgrove Road West, Foresterhill, Aberdeen AB25 2ZD, UK; Department of Internal Medicine and Radboud Center for Infectious Diseases, Radboud University Medical Center, Geert Grooteplein 28, 6525 GA Nijmegen, The Netherlands; Mimetas, Biopartner Building 2, J.H. Oortweg 19, 2333 CH Leiden, The Netherlands; NEXBIOME Therapeutics, 22 allée Alan Turing, 63000 Clermont-Ferrand, France; BIOASTER Microbiology Technology Institute, 40 avenue Tony Garnier, 69007 Lyon, France; NEXBIOME Therapeutics, 22 allée Alan Turing, 63000 Clermont-Ferrand, France; ProDigest BV, Technologiepark 94, B-9052 Gent, Belgium; Gut Microbiology Group, Rowett Institute, University of Aberdeen, Ashgrove Road West, Foresterhill, Aberdeen AB25 2ZD, UK; MRC Centre for Medical Mycology, Department of Biosciences, University of Exeter, Geoffrey Pope Building, Stocker Road, Exeter EX4 4QD, UK

**Keywords:** *Candida*, *Candida* infections, antifungal immunity, microbiota, mycobiota, fungus-host-microbiota interactions, patient variability, fungal variability, microbiota variability

## Abstract

*Candida albicans* is a major fungal pathogen of humans. It exists as a commensal in the oral cavity, gut or genital tract of most individuals, constrained by the local microbiota, epithelial barriers and immune defences. Their perturbation can lead to fungal outgrowth and the development of mucosal infections such as oropharyngeal or vulvovaginal candidiasis, and patients with compromised immunity are susceptible to life-threatening systemic infections. The importance of the interplay between fungus, host and microbiota in driving the transition from *C. albicans* commensalism to pathogenicity is widely appreciated. However, the complexity of these interactions, and the significant impact of fungal, host and microbiota variability upon disease severity and outcome, are less well understood. Therefore, we summarise the features of the fungus that promote infection, and how genetic variation between clinical isolates influences pathogenicity. We discuss antifungal immunity, how this differs between mucosae, and how individual variation influences a person's susceptibility to infection. Also, we describe factors that influence the composition of gut, oral and vaginal microbiotas, and how these affect fungal colonisation and antifungal immunity. We argue that a detailed understanding of these variables, which underlie fungal-host-microbiota interactions, will present opportunities for directed antifungal therapies that benefit vulnerable patients.

## Teaser

The complexity and variability of FunHoMic interactions between the fungal pathogen, its human host and the Microbiota strongly influence the development and outcomes of the superficial and systemic *Candida albicans* infections that plague human health worldwide.

## INTRODUCTION

Fungal pathogens have a major global impact upon human health. Estimates suggest that, at any given time, over a quarter of the world's population have a fungal infection of the skin, that 75% of women suffer at least one episode of vulvovaginal candidiasis during their lifetime, and that over a million people die each year from an invasive fungal infection (Brown *et al*. 2012). Mortality rates for those suffering systemic fungal infections are unacceptably high, reaching 50% in many cases. This is because fungal infections are often difficult to diagnose, and are particularly challenging to treat (Perlroth, Choi and Spellberg 2007; Brown *et al*. 2012; Köhler, Casadevall and Perfect 2014). There is a clear and urgent medical need for more accurate diagnostics, for safer and more effective antifungal drugs, and for host-directed therapies. The search for antifungal drug targets is somewhat constrained by the fact that, as eukaryotes, fungi share fundamental mechanisms of cell growth and division with humans. The search for diagnostic markers that can distinguish infection from fungal commensalism is especially challenging. Therefore, the development of potent new clinical tools is dependent upon a comprehensive understanding of fungal pathogenicity and antifungal immunity.

*Candida* species are amongst the top fungal killers (Brown *et al*. 2012). Of these, *Candida albicans* remains the most common cause of life-threatening systemic candidiasis, although the frequent prophylactic use of azole antifungal drugs has led to the emergence of other *Candida* species with intrinsic resistance to these drugs (Nguyen *et al*. 1996; Silva *et al*. 2012; Chowdhary, Sharma and Meis 2017). Nevertheless, in this review we focus on *C. albicans*, because a combination of three main factors arguably makes this species unique amongst fungal pathogens: (a) its lifestyle as both a commensal and potent pathogen; (b) the range and frequency of infections that it causes; and (c) its pathobiology has been studied in greater depth than most other fungal pathogens.

*Candida albicans* is an opportunistic pathogen that exists as a commensal in most individuals, and is a frequent cause of mucosal and systemic infections (See *The Fungus*). Unlike most fungal pathogens, *C. albicans* is generally considered to be obligately associated with warm-blooded animals (Odds 1988). Environmental isolates of *C. albicans* continue to be reported (Bensasson *et al*. 2019; Maciel *et al*. 2019; Opulente *et al*. 2019). However, although the existence of an environmental reservoir cannot be excluded, it is apparently not necessary for human colonisation.

*Candida albicans* is transmitted vertically from mother to child, and infections arise predominantly from the endogenous microbiota rather than other sources (d Enfert 2009; Miranda *et al*. 2009; Zhai *et al*. 2020) (see *The Microbiota*). This contrasts with other major pathogens such as *Aspergillus, Cryptococcus* and *Histoplasma* species, which are fundamentally environmental fungi that have evolved traits that promote pathogenicity in humans, possibly through their transient passage in niches that have similarities with those encountered in the human host, for example, their association with rodents or contact and evasion of amoebic predation in the environment (Steenbergen, Shuman and Casadevall 2001; Malliaris, Steenbergen and Casadevall 2004; Van Waeyenberghe *et al*. 2013; Hillmann *et al*. 2015). *Pneumocystis jirovecii* is obligately associated with humans, but this major pathogen differs from *C. albicans* in that it is unable to thrive outside its host (Liu, Fahle and Kovacs 2018). Consequently, key aspects of *Pneumocystis jirovecii* biology remain unexplored. The lifestyle of *C. albicans* even differs considerably from its distant cousin, *C*. (Brunke and Hube 2013; Kasper, Seider and Hube 2015). Genetic evidence suggests that, although it is often presumed to be a human commensal such as *C. albicans, C. glabrata* seems to be only secondarily associated with humans and is likely to have environmental reservoirs (Gabaldón and Fairhead 2019).

**Figure 1.**
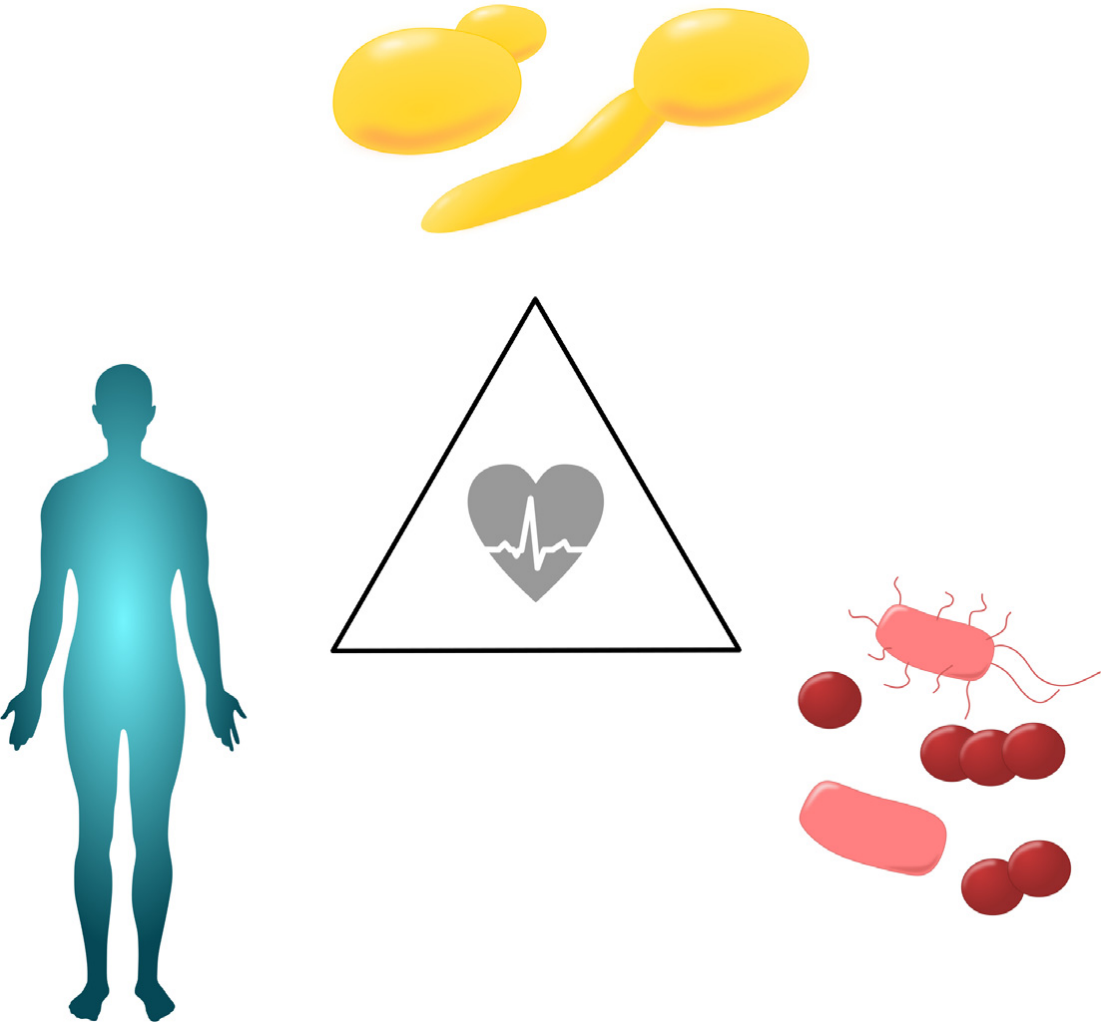
Three-way interactions between the fungus, the host and the local microbiota strongly influence the likelihood and severity of *C. albicans* infections. See text.

The biology, epidemiology, pathogenicity and immunology of *C. albicans* have been studied in greater depth than for any other fungal pathogen. This depth of knowledge provides a strong platform for studies of the relationships between the fungal pathogen, host immunity and local microbiota that lie at the heart of fungal infection (Casadevall and Pirofski 1999, 2003, 2015; Jabra-Rizk *et al*. 2016) (Fig. 1). Other major fungal pathogens infect humans by different routes to *C. albicans*, but many principles that are emerging for *C. albicans* may be applicable to these pathogens. Therefore, we present underlying principles of *C. albicans* colonisation and infection, antifungal immune defences, and the protective properties of the local microbiota in the gastrointestinal (GI) tract, oral cavity and vagina. We also address the variability that influences the *Fungus-Host-Microbiota* interplay and how this impacts infection. A detailed understanding of this tripartite interplay is essential to optimise therapeutic strategies for individual patients (d Enfert 2009; Pirofski and Casadevall 2020).

## THE FUNGUS

### C. albicans commensalism and pathogenicity

**Figure 2.**
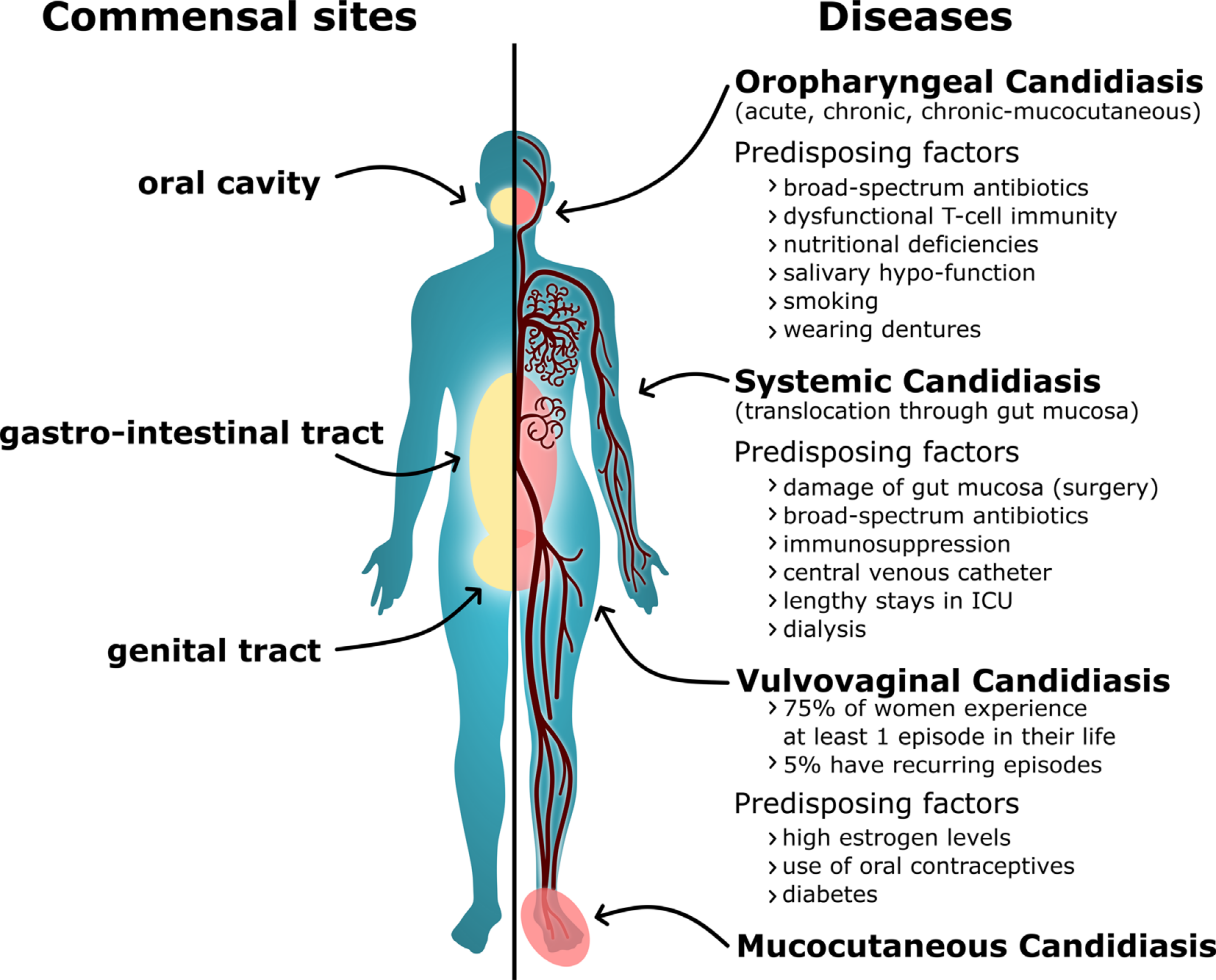
**Sites of *C. albicans* commensalism and disease on the human body**. Sites of *C. albicans* commensalism (left side) include the oral cavity, gastrointestinal tract (gut) and the genital tract. *C. albicans* can infect these sites (right side) to cause oropharyngeal or vulvovaginal candidiasis. *C. albicans* can also cause systemic infections of the blood and internal organs, which often arise *via* translocation of *C. albicans* from the gut into the bloodstream. *Candida albicans* also causes mucocutaneous infections of the skin and nails. Factors that predispose individuals to such infections are listed. See text.

*C. albicans* frequently inhabits the oral, vaginal and GI mucosa of healthy individuals as a harmless commensal (Ghannoum *et al*. 2010; Drell *et al*. 2013; Nash *et al*. 2017) (Fig. 2). Indeed, *C. albicans* is present on the mucosa of most people in most human populations (Neville, d Enfert and Bougnoux 2015; Prieto *et al*. 2016; Mishra and Koh 2018). However, this fungus can cause infections if the local microbiota becomes perturbed, normal tissue barriers are weakened or immune defences become compromised.

Mucosal infections, characterised by fungal colonisation (i.e. overgrowth) associated with an inflammatory host response, are extremely common and can have a major impact upon the quality of life for many individuals (Fig. 2). For instance, most women of reproductive age (75%) will experience at least one episode of VVC (‘thrush’) in their lifetime, and up to 9% suffer from recurrent VVC, as defined by multiple episodes of vaginitis per annum (Foxman *et al*. 2013; Yano *et al*. 2019; Rosati, Bruno, Jaeger, Ten Oever *et al*. 2020). Risk factors for VVC include high estrogen levels, the use of oral contraceptives and uncontrolled diabetes. However, episodes can be idiopathic (i.e. of unknown cause) and VVC, unlike oral candidiasis, can occur in apparently healthy individuals (see *Innate antifungal responses*).

Oropharyngeal candidiasis (OPC) can broadly be classified into three main conditions, namely acute, chronic and chronic mucocutaneous candidiasis syndromes (Vila *et al*. 2020) (Fig. 2). Predisposing factors include nutritional deficiencies, local dysbiosis, salivary hypo-function, smoking, wearing dentures and dysfunctional T-cell immunity due to genetic alterations or other infections. Indeed, OPC is the most frequently diagnosed oral opportunistic infection in HIV-positive individuals and many acute cases are caused by broad-spectrum antibiotic treatments (Samaranayake 1992; Vila *et al*. 2020).

Life-threatening systemic *C. albicans* infections can arise when the fungus enters the bloodstream (Fig. 2). Candidaemia is the fourth most common nosocomial bloodstream infection in North America (Pfaller and Diekema 2010), but the incidence of invasive candidiasis in European countries is generally lower (Meyer *et al*. 2013; Yapar 2014). The presence of a central venous catheter, dialysis, antibiotic treatment, lengthy stays in intensive care units (ICUs), recent major surgery, and receiving total parenteral nutrition are among the predisposing factors for systemic candidiasis (Pappas *et al*. 2018). Most disseminated infections arise from *Candida* escaping the patient's own GI tract (Miranda *et al*. 2009; Gouba and Drancourt 2015; Zhai *et al*. 2020). Systemic infections arise when host defences are compromised by, for example, damage to the intestinal barrier (e.g. surgery or trauma), medically induced immunosuppression (corticosteroids or chemotherapy-induced neutropenia), or the use of broad-spectrum antibiotics (Pappas *et al*. 2018). A combination of these factors is typically needed to allow *C. albicans* to translocate from the gut (Koh *et al*. 2008; Papon, Bougnoux and d Enfert 2020). Once in the blood, *C. albicans* can disseminate to almost all organs including kidney, liver, and spleen (Pappas *et al*. 2018). The mortality rate for these infections, which varies across geographical regions, is reported to lie between 10% and 47% despite the availability of antifungal therapies (Brown *et al*. 2012). This is unacceptably high.

Clearly, knowledge about the factors and conditions that promote *C. albicans* commensalism or opportunism is important for an understanding of the mechanisms that underlie the transition from commensalism to pathogenicity. Much work has focussed on the virulence factors and fitness attributes that promote *C. albicans* infection (see *Virulence Factors* and *Fitness attributes*). However, the pathogenesis of *C. albicans* also depends on the host site of colonisation (Fidel *et al*. 2020). *Candida albicans* asymptomatically inhabits the oral mucosa and only causes infection when host defences are weakened. In contrast, *C. albicans* is an immunoreactive coloniser during vulvovaginal infection, eliciting host damage *via* a hyperactive immune response. Meanwhile, systemic infections are mostly nosocomial and are generally associated with predisposing conditions. The fungus is able to cause these different types of infection by tuning the expression of its arsenal of virulence factors and fitness attributes to the local niche.

### Virulence factors

#### Cellular polymorphism

**Figure 3.**
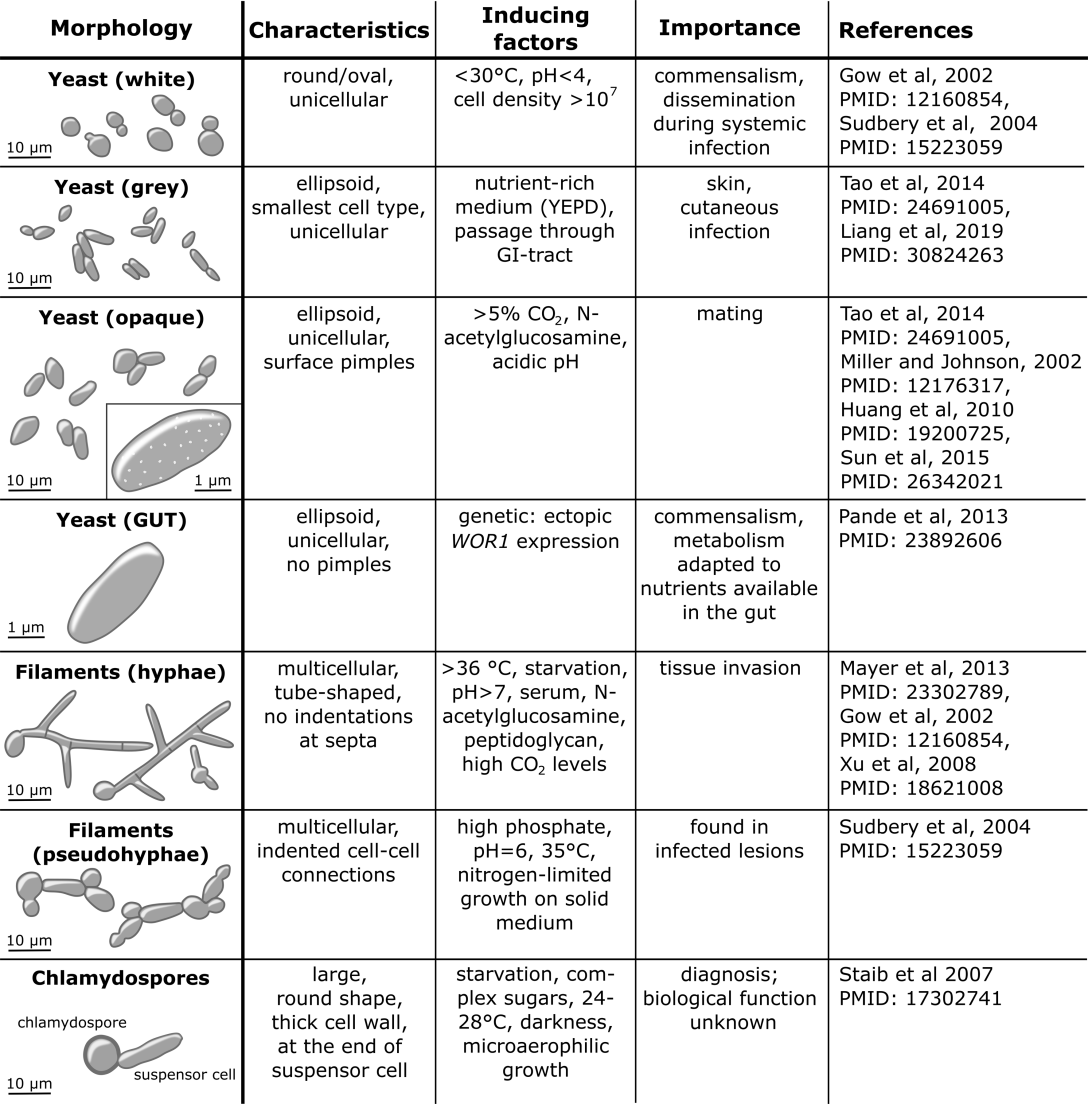
***Candida albicans* is polymorphic, displaying a range of cellular growth forms**. *C. albicans* yeast cells can undergo phenotypic switching between white, grey and opaque growth forms that present with different shapes and cell surface characteristics (Gow, Brown and Odds 2002; Sudbery, Gow and Berman 2004; Xu *et al*. 2008; Huang *et al*. 2009; Mayer, Wilson and Hube 2013; Tao *et al*. 2014; Sun *et al*. 2015). These forms are induced in response to different environmental inputs, and hence are associated with different types of infection (Gow, Brown and Odds 2002). Significantly, the opaque form is associated with efficient mating in *C. albicans* (Miller and Johnson 2002), with grey cells displaying an intermediate mating competence between opaque and white cells (Tao *et al*. 2014). The gastrointestinally induced transition (GUT) phenotype is observed in *C. albicans* cells that ectopically express *WOR1* (Pande, Chen and Noble 2013), a key regulator of commensalism. The transition from (white) yeast cells to pseudohyphae or hyphae is stimulated by a wide variety of environmental inputs, which include elevated temperatures, pH and peptidoglycan. Pseudohyphae can be distinguished from hyphae on the basis of the position of the septal junction between a mother yeast cell and its filamentous daughter, and by the presence of invaginations at these septal junctions in pseudohyphae, but not hyphae (Merson-Davies and Odds 1989; Sudbery 2001; Sudbery, Gow and Berman 2004). *Candida albicans* can be induced to form chlamydospores under specific environmental conditions (Jansons and Nickerson 1970), but the biological significance of this growth form remains obscure (Staib and Morschhäuser 2007). See text.

The polymorphic nature of *C. albicans* is integral to both commensalism and pathogenesis. This fungus is able to switch reversibly between different growth forms and morphologies (Noble, Gianetti and Witchley 2017) (Fig. 3). Depending upon the environmental conditions, *C. albicans* can grow as unicellular yeast cells, pseudohyphae, or true hyphae that lack invaginations at septal junctions (Sudbery, Gow and Berman 2004). Also, depending on the presence of certain environmental cues, *C. albicans* can undergo phenotypic switching to interchange reversibly between white, grey and opaque phenotypes, each of which displays distinct yeast cell and colony morphologies, and gene expression profiles. Furthermore, a gastrointestinally induced transition (GUT) phenotype has been described for *C. albicans* cells that ectopically overexpress the Wor1 regulator which, together with Efg1, controls white-grey-opaque switching (Pande, Chen and Noble 2013). Phenotypic switching is a strictly regulated process that seems to be associated with commensalism, host niche adaptation, mating, immune evasion and virulence (Miller and Johnson 2002; Morschhäuser 2010; Pande, Chen and Noble 2013; Xie *et al*. 2013; Tao *et al*. 2014). Finally, *C. albicans* can differentiate to form chlamydospores, enlarged thick-walled cells, under nutrient limitation, low temperature and microaerophilia (Staib and Morschhäuser 2007; Böttcher *et al*. 2016) (Fig. 3).

**Figure 4.**
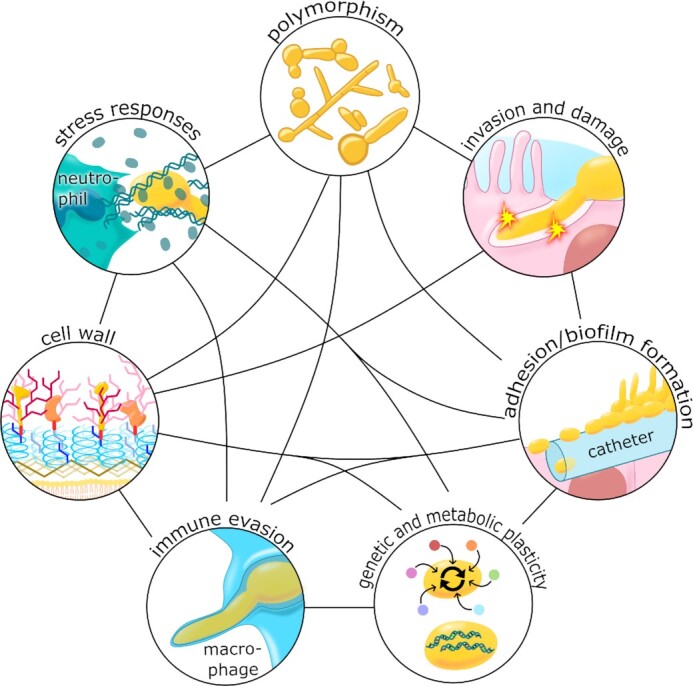
**A combination of virulence factors and fitness attributes promote *C. albicans* virulence**. *Polymorphism*: The ability of *C. albicans* to undergo morphological transitions allows it to adapt to different growth conditions, adhere to biotic and abiotic surfaces, invade cells and tissue, and escape from immune cells. *Invasion and damage*: A combination of induced endocytosis and active penetration promote fungal invasion of host tissues, and the accumulation of the toxin, candidalysin, in the invasion pocket leads to pore formation and host cell damage. *Adhesion/biofilm formation*: The battery of adhesins promotes fungal adhesions to biological and abiotic surfaces, which can lead to the development of biofilms, for example on medical devices such as catheters. *Genetic and metabolic plasticity*: *Candida albicans* displays a high degree of metabolic flexibility, which allows it to adapt rapidly to diverse host niches. This fungus also displays great genetic plasticity, which permits rapid evolutionary adaptation to selective pressures and stresses such as exposure to antifungal drugs. *Stress responses*: *Candida albicans* activates robust stress responses following exposure to host imposed stresses, including ROS and RNS, which enhances fungal survival following immune attack, for example. *Cell wall*: As well as maintaining cell morphology, the robust cell wall provides protection against host-imposed stresses including changes in osmolarity. *Immune evasion*: *Candida albicans* has evolved a variety of immune evasion strategies that include the modulation of PAMP exposure at the cell surface to evade immune recognition, and phagocytic escape mechanisms to evade killing by innate immune cells. See text.

Both yeast and hyphal morphologies are necessary for the full virulence of *C. albicans* (Lo *et al*. 1997; Murad *et al*.^2001^; Saville *et al*. 2003; Jacobsen *et al*. 2012) (Fig. 4). However, it is generally thought that yeast cells are well suited to dissemination, and hyphal cells to tissue invasion (Gow, Brown and Odds 2002). The yeast-to-hypha transition is accompanied by an extensive change in gene expression profile, in cell wall structure, and by the expression of many virulence factors (Jacobsen *et al*. 2012; Mayer, Wilson and Hube 2013; Chen *et al*. 2020). The change in morphology can be triggered by many environmental factors present in host niches, such as physiological temperatures (>36°C), starvation, an ambient pH of >7, the presence of serum, *N*-acetylglucosamine, or elevated CO_2_ levels (Mayer, Wilson and Hube 2013). Furthermore, hyphal development is triggered by the bacterial cell wall component, peptidoglycan (Xu *et al*. 2008), which is of particular relevance to fungus-host-microbiota interactions. Not surprisingly given the complexity of environmental inputs and cellular outputs, yeast-hypha morphogenesis is regulated by a complex signalling network that includes the cAMP-protein kinase A, Efg1, Cph1, Czf1, Hog1 and Nrg1 pathways (Basso *et al*. 2019; Kadosh 2019; Kornitzer 2019).

During experimental colonisation of the murine GI tract, *C. albicans* was found to thrive in the yeast form (Vautier *et al*. 2015). The basis for the predominance of the yeast morphology during gut colonisation remains unclear, but unknown selective pressures favour growth in the yeast form during experimental GI colonisation in mice during GI dysbiosis (Tso *et al*. 2018). Furthermore, mucus covering the epithelium, tight junctions between epithelial cells, and the lamina propria serve as physical barriers that limit *C. albicans* translocation and dissemination from the gut (Yan, Yang and Tang 2013; Arevalo and Nobile 2020). Mucin, the main component of mucus, prevents hyphal formation (Kavanaugh *et al*. 2014) and reduces the adherence of *C. albicans* to epithelial cells (de Repentigny *et al*. 2000). Similarly, saliva can exert anti-*Candida* effects in the oral cavity (Hibino *et al*. 2009) (see *Oral cavity*). More recent work suggests that filamentous forms can exist in certain parts of the GI tract where the microenvironment favours hyphal development (Witchley *et al*. 2019). Only under certain circumstances, for example when a perturbed microbiota and a compromised immune system lose control over *C. albicans* growth (see *The Host* and *The Microbiota*), the fungus can switch from commensalism to pathogenicity (Gow *et al*. 2011).

Significantly, the host can exploit the yeast-to-hypha transition to discriminate between colonisation and infection. This involves a biphasic innate immune response at the epithelial barrier (Moyes *et al*. 2010; Roselletti *et al*. 2019). The first signalling event is triggered by fungal cell wall components, notably β-glucans and mannans, irrespective of cell morphology (Moyes *et al*. 2010). The second, danger response, is only induced once a high fungal burden is achieved, hypha formation occurs, and the hypha-associated toxin candidalysin is expressed (see *Host damage*) (Moyes et al. 2010, 2016). This leads to the secretion of pro-inflammatory cytokines and phagocyte infiltration, which promote fungal clearance. In addition, phagocytes can distinguish hyphae from yeast cells based on the shorter cell wall mannan fibrils of hyphal cells (Cheng *et al*. 2011). Macrophages also respond to hyphal load, in part through the degree of metabolic competition between host and pathogen, displaying reduced activation of the NLRP3-inflammasome pathway at low hyphal burdens (Tucey *et al*. 2020; Westman *et al*. 2020). Thus, while hypha formation is critical for invasion (see *Invasion mechanisms*), the host has developed mechanisms to recognise the invasive form of *C. albicans*. Therefore, hypha formation seems to be detrimental for *C. albicans* commensalism.

#### Adhesion to abiotic and biotic surfaces

*Candida albicans* cells can adhere to each other as well as to host cells and abiotic surfaces, such as catheters or dental implants, which promotes colonisation and the formation of biofilms (de Groot *et al*. 2013; Lohse *et al*. 2018) (Fig. 4). *Candida albicans* forms hyphae upon sensing contact to a surface (Kumamoto 2008) and hyphae express specific adhesins that promote adhesion to such surfaces (de Groot *et al*. 2013).

The Agglutinin-Like Sequence (*ALS*) genes represent one family of adhesins in *C. albicans*, some of which are morphogenetically regulated (Hoyer and Cota 2016). Analogous adhesin families are present in other pathogenic and non-pathogenic fungi (Butler *et al*. 2009). Als adhesins have a three-domain structure: the N-terminal ligand-binding domain (Lin *et al*. 2014); internal tandem repeats; and the C-terminal domain, which binds the cell wall *via* a modified glycosylphosphatidylinisotol (GPI)-anchor. In *C. albicans*, the *ALS* gene family has nine members, each of which displays a high degree of variability between alleles and strains, particularly in the length of the central repetitive domain (Hoyer and Cota 2016). Als3, the best-studied Als family member, has multiple functions. It binds heterogenous ligands including cadherins, ferritin and a *Streptococcus gordonii* surface protein (Phan *et al*. 2007; Almeida *et al*. 2008; Bamford *et al*. 2015). Als3 also acts as an invasin that promotes fungal invasion of host cells (Phan *et al*. 2007) and iron assimilation (Almeida *et al*. 2008). This makes Als3 an asset for the fungus during infection, but also a potential target for anti-*Candida* therapies (Edwards *et al*. 2018; Marc *et al*. 2018; Kioshima *et al*. 2019).

The hyphal wall protein 1 (Hwp1), is specifically expressed during hyphal growth (Staab, Ferrer and Sundstrom 1996), and is the founding member of a second family of five adhesins in *C. albicans* (de Groot *et al*. 2013). Members of the Hwp family are required for both virulence and mating. The N-terminus of Hwp1 is enriched in glutamine residues that become cross-linked to the host extracellular matrix by host transglutaminases (Staab *et al*. 1999). In contrast, Yeast wall protein 1 (Ywp1) appears to counteract adhesion leading to the release of yeast cells from surfaces, which might promote fungal dissemination during systemic candidiasis (Granger 2012).

A third family of putative adhesins is encoded by the twelve-member *HYR* gene family (de Groot *et al*. 2013). The founding member of this family, *HYR1*, like *ALS3* and *HWP1*, is expressed during hyphal development (Bailey *et al*. 1996). This *HYR* family has been less well characterised than the *ALS* and *HWP* families. Nevertheless, it adds to the adhesins that *C. albicans* expresses to promote robust adhesion to each other, abiotic surfaces or the host.

#### The cell wall

Both cellular polymorphism and adhesion are intimately associated with the *C. albicans* cell wall, the organelle that maintains the morphology of the *C. albicans* cell and that supplies the scaffold for most adhesin proteins (Klis, de Groot and Hellingwerf 2001; de Groot *et al*. 2004; Gow, Latge and Munro 2017) (Fig. 4). The cell wall also provides osmotic stability and protects against environmental stresses. It is robust in exerting control of cell shape, and yet elastic during responses to acute osmotic stress (Ene *et al*. 2015). Furthermore, the cell wall is a highly flexible organelle, in that it displays a high capacity to adapt and remodel itself in response to environmental challenges or antifungal drugs (Sosinska *et al*. 2008; Ene *et al*. 2012; Childers *et al*. 2019).

The *C. albicans* cell wall is a two-layered structure. The inner layer consists of chitin, β-1,3- and β-1,6-glucans and mannoproteins. The outer layer is enriched in mannan fibrils that are anchored to mannoproteins cross-linked to the inner layer of the wall (Kapteyn *et al*. 2000; Gow *et al*. 2011; Gow, Latge and Munro 2017). Chitin comprises about 2%–3% of the mass of the yeast cell wall, but represents an important structural component that is essential for the integrity of the cell wall. The main structural polysaccharide of the *C. albicans* cell wall is β-glucan, which accounts for 50%–60% of the mass of the yeast cell wall (Shepherd 1987; Klis, de Groot and Hellingwerf 2001). The β-1,3-glucan network provides the platform for covalent attachment of chitin, β-1,6-glucan and mannoproteins.

Two main classes of cell wall mannoproteins have been defined in *C. albicans*. GPI-anchored proteins are the more abundant class. As their name suggests, these are linked *via* modified GPI anchors to β-1,6-glucan which, in turn, are covalently attached to β-1,3-glucan (Kapteyn *et al*. 2000). Pir proteins (proteins with internal repeats) are covalently attached to β-1,3-glucan directly (Kapteyn *et al*. 2000). *C. albicans* cell wall mannoproteins contribute 30–40% of the mass of the yeast cell wall (Kapteyn *et al*. 2000) and are adorned with *N*- and/or *O*-linked oligosaccharides. The *O*-linked oligosaccharides are often linked to serine-threonine-rich repeats (e.g. in ALS adhesins: see *Adhesion to abiotic and biotic surfaces*) and are thought to confer rod-like structures to these domains (Gatti *et al*. 1994). *N*-linked mannans are highly branched structures that form the fibrils in the outer layer of the wall (Gow, Latge and Munro 2017; Childers *et al*. 2019). The functions of about 70% of cell wall mannoproteins remain obscure, but some are known or suspected to be involved in the infection process (De Groot, Ram and Klis 2005; Richard and Plaine 2007).

The cell wall is an attractive target for antifungal therapy because it is essential for fungal viability and not present on human cells. Consequently, β-1,3-glucan synthesis is the target for a major class of antifungal drugs in clinical use—the echinocandins (Odds, Brown and Gow 2003). Significantly, in the context of this review, the cell wall is also the first point of direct contact with the host, and therefore a prime target for immune recognition (see *Fungal recognition*) (Netea *et al*. 2008; Erwig and Gow 2016).

#### Biofilm formation

*Candida albicans* can form florid biofilms on biological surfaces and also abiotic surfaces such as catheters, dentures and prosthetic joints (Fig. 4). Biofilms are a common source of nosocomial infection (Ramage *et al*. 2005; Nobile and Johnson 2015), and they increase therapeutic challenges by enhancing the resistance to antifungal drugs (Taff *et al*. 2013).

Biofilm formation is initiated by adhesion of *C. albicans* cells to the surface (see *Adhesion to abiotic and biotic surfaces*). Surface contact stimulates hyphal growth (see *Cellular polymorphism*), the development of the biofilm and the production of extracellular matrix, and the biofilm matures into an organised and robust structure (Nobile and Johnson 2015). Biofilm formation is a complex process that is controlled by a network of transcription factors and that integrates the expression of adhesins, cellular morphogenesis and the production of extracellular matrix. Accordingly, biofilm formation is controlled by a complex transcriptional network of over 1000 genes (Finkel and Mitchell 2011; Nobile *et al*. 2012; Lohse *et al*. 2018). These target genes include members of the *ALS* family, which are essential for biofilm formation and enhance aggregation between fungal cells *via* amyloid formation (Dehullu *et al*. 2019; Vida Ho *et al*. 2019).

Biofilm maturation is followed by the dispersal of yeast cells from the biofilm, which promotes fungal dissemination. *Candida albicans* cells dispersed from biofilms are distinct from planktonically grown yeast. These dispersed cells are transcriptionally reprogrammed to utilise alternative carbon sources and they acquire nutrients, such as zinc and amino acids, with higher efficiency (Uppuluri *et al*. 2018).

*Candida albicans* clinical isolates display a high degree of heterogeneity with respect to their capacity to form biofilms and the underlying regulatory network (Sherry *et al*. 2017; Huang *et al*. 2019), and biofilm-forming ability has been associated with high mortality rates in patients (Rajendran *et al*. 2016). In the clinical setting, the situation is further complicated by the formation of multispecies biofilms. For example, *C. albicans* is commonly associated with *Streptococcus* and *Actinomyces* species in dental samples, with *Lactobacillus* species in vaginal specimens, and with *Pseudomonas* in the lungs of cystic fibrosis patients (Hogan, Vik and Kolter 2004; Falagas, Betsi and Athanasiou 2006; Bamford *et al*. 2009; Bandara *et al*. 2009; Cruz *et al*. 2013; Bamford *et al*. 2015) (see *Synergistic and antagonistic interactions between kingdoms*). These inter-kingdom associations affect *C. albicans* growth, morphogenesis and drug resistance (Hogan, Vik and Kolter 2004).

#### Invasion mechanisms

The invasion of host cells and tissues provides an effective strategy to access more nutrients, avoid competition with other members of the microbiota, and potentially escape antimicrobial treatment (Fig. 4). Two distinct routes for the invasion of epithelia and endothelia are known for *C. albicans*: induced endocytosis and active penetration (Dalle *et al*. 2010; Wächtler *et al*. 2012). Induced endocytosis is mediated by the fungal proteins Ssa1 and Als3 (the adhesin-invasin, mentioned above), both of which are present on the cell wall. These proteins bind to E- and N- cadherins on epithelial and endothelial cells, as well as to the epithelial growth factor receptor of oral epithelial cells, to induce the uptake of fungal cells through remodelling of the host cytoskeleton (Phan *et al*. 2007; Moreno-Ruiz *et al*. 2009; Sun *et al*. 2010; Solis *et al*. 2017). Active penetration is achieved through the growth of hyphae into host tissue. This is the dominant route of fungal invasion into oral epithelial cells and the only observed route in enterocytes (Dalle *et al*. 2010; Wächtler *et al*. 2012).

As stated, the GI tract is a major reservoir for resident *C. albicans* (Nucci and Anaissie 2001; Gouba and Drancourt 2015), and hence fungal translocation across intestinal barriers is a common source of systemic candidiasis. This translocation can be promoted by injury, GI pathologies or medical interventions. Nevertheless, the translocation of *C. albicans* cells through enterocytes in a transcellular manner, and subsequent necrotic host cell death, is a major mechanism by which the fungus crosses the epithelial barrier (Allert *et al*. 2018). *C. albicans* directs physical force against cell membranes to stretch and rupture host cell membranes *via* a combination of hyphal growth and secreted virulence factors (Wächtler *et al*. 2012). Meanwhile, host cells employ several mechanisms to expand and repair membranes to limit this damage (Westman, Hube and Fairn 2019). This leads to the formation of the so-called ‘invasion pocket’ where the invading hypha is surrounded by host membrane (Moyes *et al*. 2016). The confined space around the hypha, within the invasion pocket, permits the accumulation of *C. albicans* secreted virulence factors to high local concentrations that cause further damage and stress to the host (Dalle *et al*. 2010; Moyes *et al*. 2016; Allert *et al*. 2018).

#### Host damage

The ability to damage host cells provides *C. albicans* with access to cytoplasmic nutrients, and the fungus possesses an extensive weaponry to impose damage (Fig. 4). Damaging factors that accumulate in the invasion pocket include secreted hydrolases such as phospholipase B1, lipases and secreted aspartic proteases (Saps) that degrade host membranes, proteins and extracellular matrix releasing nutrients (Mukherjee *et al*. 2001; Naglik, Challacombe and Hube 2003; Schofield *et al*. 2005). *Candida albicans* also expresses candidalysin—a pore forming α-helical peptide toxin that is encoded by the *ECE1* gene (Moyes *et al*. 2016). Pores formed in the host cell membrane by candidalysin probably leak cytoplasmic contents into the invasion pocket, thereby providing additional nutrients for the fungus. This may include access to essential micronutrients such as iron and zinc. Specific proteins bind these micronutrients, which are then endocytosed or transported across the fungal cell membrane *via* specific transporters. For example, members of the Rbt5-family transport heme across the cell wall (Kuznets *et al*. 2014; Nasser *et al*. 2016). Also, zinc is acquired *via* the zincophore Pra1 (pH-regulated antigen 1), which is released into the extracellular space and then, when loaded with zinc, is transported back into the fungus by the zinc transporter Ztr1 (Citiulo *et al*. 2012).

### Fitness attributes

Fitness attributes are factors that promote fungal virulence by enhancing the physiological robustness of the fungus in host niches, rather than by interacting directly with the host. In *C. albicans*, fitness attributes include metabolic flexibility combined with potent nutrient acquisition systems, and robust stress response mechanisms (Mayer, Wilson and Hube 2013; Brown, Budge *et al*. 2014; Brown, Brown, *et al*. 2014). These promote the success of *C. albicans* both as a commensal and as a pathogen of humans.

#### Flexible metabolic adaptation

Metabolic adaptability is critical during *C. albicans* transitions between commensalism and pathogenicity (Fig. 4). This was highlighted by an elegant screen for regulatory circuitry that drives the commensal and pathogenic states in *C. albicans* (Pérez, Kumamoto and Johnson 2013). Much of this circuitry is involved in the regulation of metabolism. Metabolic regulation in *C. albicans* is integrated with the control of virulence factors and stress resistance through major regulatory hubs such as Efg1, Tup1, Nrg1, Hog1 and Gcn4 (Murad *et al*. 2001; Tripathi *et al*. 2002; Doedt *et al*. 2004; Alonso-Monge *et al*. 2009). Therefore, metabolic adaptation is essential for commensalism and virulence, and is intimately linked with other pathogenicity traits (Mayer, Wilson and Hube 2013; Brown, Brown, *et al*. 2014).

Glucose is a preferred carbon source for *C. albicans*, but under glucose-limiting conditions, such as in the colon or after entrapment in the phagosome, *C. albicans* tunes its metabolism to feed on alternative carbon sources (Lorenz, Bender and Fink 2004; Barelle *et al*. 2006). Even when glucose becomes available, *C. albicans* can simultaneously utilise alternative carbon sources through multiple pathways (Sandai *et al*. 2012; Childers *et al*. 2016). This metabolic flexibility allows the fungus to adapt to contrasting host niches. Significantly, it also influences the tolerance of *C. albicans* to antifungal drugs and environmental stresses (Ene *et al*. 2012). For example, growth on lactate protects against osmotic and cell wall stresses while utilisation of amino acids and *N*-acetylglucosamine (GlcNAc) increases fungal resistance to reactive oxygen and nitrogen species (ROS and RNS, respectively) (Williams and Lorenz 2020). These alternative carbon sources appear to serve as niche-specific signals that prime the fungus for impending challenges, pointing to the dexterity of *C. albicans* not only to adapt, but also to anticipate, local stress conditions (Brown, Budge *et al*. 2014; Alistair J P Brown *et al*. 2019; Williams and Lorenz 2020). The metabolic flexibility of *C. albicans* extends well beyond carbon metabolism to include nitrogen, phosphate and micronutrient assimilation (Lorenz, Bender and Fink 2004; Yin *et al*. 2004; Vylkova *et al*. 2011; Ene *et al*. 2014; Ikeh *et al*. 2016).

Micronutrients, such as iron and zinc, are essential for structural integrity and physiological processes in *C. albicans*. However, in response to infection, through a process called nutritional immunity, the host limits the availability of these micronutrients and exposes the fungus to toxic levels of other species such as copper ions (Noble 2013; Potrykus *et al*. 2013; Mackie *et al*. 2016; Sprenger *et al*. 2018). In response, the fungus activates efficient micronutrient acquisition strategies. High affinity iron uptake involving a cyclic iron reduction pathway (iron reductase, multicopper ferroxidase and iron permease) is activated to take over from low affinity ferritin-iron uptake *via* the protein Als3, which is operational in hyphae during iron-replete conditions (Wilson, Naglik and Hube 2016; Bairwa, Hee Jung and Kronstad 2017). *Candidaalbicans* can also assimilate iron from heme and hemoglobin using Common in Fungal Extracellular Membrane (CFEM) proteins, and can scavenge siderophores synthesised by other microorganisms using the Arn1/Sit1 ferrichrome transporter (Bairwa, Hee Jung and Kronstad 2017). Transcriptional circuitry involving Sef1, Sfu1 and Hap43 control iron homeostasis by activating iron assimilation mechanisms when iron is limiting, and by repressing iron uptake when it is in excess (Chen *et al*. 2011; Noble 2013). *Candida albicans* utilises two uptake mechanisms to scavenge zinc. The first, which operates mainly at acidic pHs, involves uptake *via* the Zrt2 transporter into the cytoplasm (Crawford *et al*. 2018). The second, which is functional at neutral pHs, entails zincophore-mediated zinc scavenging through a secreted protein, Pra1 and uptake *via* the transporter Zrt1 (Citiulo *et al*. 2012; Wilson 2015; Crawford *et al*. 2018). *C. albicans* responds to zinc limitation by forming goliath cells (enlarged and spherical yeasts that exhibit enhanced adhesion) and avoids zinc toxicity by rapidly compartmentalizing zinc in storage vacuoles called zincosomes (Malavia *et al*. 2017; Crawford *et al*. 2018).

#### Robust stress responses

Fungal pathogens generally display robust responses to certain stresses, particularly oxidative stress (Brown *et al*. 2017) (Fig. 4). *Candida albicans* is resistant to significantly higher levels of ROS than its distant cousin, *Saccharomyces cerevisiae* (Jamieson, Stephen and Terrière 1996; Nikolaou *et al*. 2009) and this helps the fungus to counter toxic ROS produced by innate immune cells, before and during phagocytic attack (Miramón *et al*. 2012). *C. albicans* and other fungal pathogens counteract acute exogenous oxidative stresses by inducing genes involved in ROS detoxification (e.g. catalase and superoxide dismutases), the synthesis of antioxidants (e.g. glutathione and thioredoxin), and the repair of ROS-mediated damage to DNA, proteins and lipids (Enjalbert, Nantel and Whiteway 2003, Enjalbert *et al*. 2006; Znaidi *et al*. 2009). The inactivation of key regulators of the response in *C. albicans* (Cap1, Skn7 and Hog1) compromises oxidative stress resistance (Alarco and Raymond 1999; Singh *et al*. 2004; Smith *et al*. 2004). Virulence is attenuated by the inactivation of the Hog1 stress activated protein kinase (Alonso-Monge *et al*. 1999; Cheetham *et al*. 2011), but only to a minor extent by the loss of Cap1 or Skn7 (Singh *et al*. 2004; Jain *et al*. 2013). The overexpression of catalase, which detoxifies hydrogen peroxide, enhances oxidative stress resistance *in vitro*, and yet, counterintuitively, reduces the virulence of *C. albicans* (Román *et al*. 2016; Pradhan *et al*. 2017). This is because overexpression of this abundant ferroprotein places an undue demand for the essential micronutrient, iron, under iron limiting conditions *in vivo* (Pradhan *et al*. 2017). Clearly, numerous and potentially opposing, selective pressures must be balanced to optimise fungal fitness in a particular host niche.

While much attention has focussed on oxidative stress, *C. albicans* faces other forms of environmental stress in the host, including nitrosative, osmotic and thermal stresses. Innate immune cells expose *C. albicans* to RNS) in an attempt to kill and clear the fungus. *C. albicans* responds by activating genes involved in RNS detoxification (such as the flavohemoglobin, Yhb1), glutathione synthesis and recycling, and the repair of RNS-mediated damage (Hromatka, Noble and Johnson 2005; Tillmann *et al*. 2015). The response to nitrosative stress is driven by the transcription factor Cta4 and Hog1 (Chiranand *et al*. 2008; Herrero-de-Dios *et al*. 2018). The inactivation of *YHB1*, *CTA4* or *HOG1* attenuates nitrosative stress resistance and virulence (Alonso-Monge *et al*. 1999; Hromatka, Noble and Johnson 2005; Chiranand *et al*. 2008; Cheetham *et al*. 2011; Miramón *et al*. 2012).

*Candida albicans* cells thrive in niches with different osmolarities (e.g. on skin, in the oral cavity or GI tract), and yet must maintain osmo-homeostasis to grow. Hypo- and hyper-osmotic challenges are countered by modulating the levels of intracellular osmolytes. For example, *C. albicans* upregulates the synthesis and accumulation of glycerol and arabitol in response to hyperosmotic challenges (San José *et al*. 1996; Kayingo and Wong 2005). This response is regulated at both transcriptional and post-transcriptional levels by the evolutionarily conserved Hog1 MAP kinase signalling pathway (Smith *et al*. 2004; Enjalbert *et al*. 2006).

*Candida albicans* must also restore and maintain proteostasis in the face of thermal challenges, even within the mammalian host (Nicholls *et al*. 2011). Even mild increases in temperature lead to activation of the so-called heat shock response (Leach, Tyc *et al*. 2012), which is regulated by an autoregulatory circuit involving the heat shock transcription factor (Hsf1) and heat shock protein 90 (Hsp90) (Leach, Budge *et al*. 2012). The response involves the induction of functions involved in protein refolding and protein degradation to repair or recycle damaged proteins (Nicholls *et al*. 2009; Leach *et al*. 2016). The heat shock response is integrated with key virulence attributes in *C. albicans* such as yeast-hypha morphogenesis, adhesion and the ability to damage epithelial cells (Shapiro *et al*. 2009; Leach *et al*. 2016). Consequently, the inactivation of the response attenuates virulence (Nicholls *et al*. 2011).

*Candida albicans* can thrive over an extremely wide range of ambient pHs, from pH 2 to 10 (Vylkova *et al*. 2011). pH responses are particularly relevant given the ability of *C. albicans* to colonise host niches with contrasting pHs such as the vagina (acidic), GI tract (acidic to mildly alkaline) and blood (neutral). These pH responses, which are regulated in part by the evolutionarily conserved Rim101 pathway (Davis, Wilson and Mitchell 2000), are tightly integrated with metabolic adaptation, nutrient acquisition and morphogenesis (Davis *et al*. 2000). Yeast-hypha morphogenesis in *C. albicans* is regulated in response to ambient pH (Buffo, Herman and Soll 1984; Porta *et al*. 1999; Chen *et al*. 2020; Villa *et al*. 2020). Ambient pH also affects trace metal solubility, and consequently, micronutrient assimilation strategies in *C. albicans* are regulated in response to pH (Noble 2013; Wilson 2015; Crawford *et al*. 2018). Significantly, *C. albicans* is not simply reactive to pH: it can proactively alkalinise its microenvironment through the catabolism of polyamines and amino acids, leading to the release of ammonia and/or CO_2_ (Mayer *et al*. 2012; Vylkova and Lorenz 2014; Danhof *et al*. 2016; Vylkova 2017). Interestingly, lactate production by a co-commensal in the oral cavity, *Streptococcus mutans*, provides carboxylic acid substrates that are sufficient to promote *C. albicans-*mediated alkalinisation of the microenvironment (Danhof *et al*. 2016; Willems *et al*. 2016).

#### Immune evasion

Immune evasion can be viewed as an additional type of fitness attribute because it promotes the physiological robustness of the fungus in the host (Fig. 4). *Candida albicans* has evolved a variety of mechanisms through which it can reduce recognition by immune cells, decrease the efficacy of antimicrobial killing mechanisms, escape immune cells following engulfment, and manipulate the immune system (see *Innate antifungal responses* and *Fungal countermeasures* for more detail). During co-evolution with its host, *C. albicans* has even developed mechanisms by which it can anticipate, and protect itself against, imminent immune attack.

Clearly, *C. albicans* possesses an array of powerful fitness attributes through which this fungus tunes its physiology to counter environmental challenges presented by the host. Significantly, the fungus not only adapts to host-defined conditions, but can also anticipate impending challenges, and actively modulate its microenvironment.

### *Candida albicans* epidemiology and variability

The flexibility of *C. albicans*, which underlies its success as a commensal and a pathogen, is also reflected at the genetic level (Fig. 4). Clinical isolates of *C. albicans* are generally diploid, with a haploid genome size of 16 Mb, organised into eight chromosomes. However, isolates display high levels of sequence heterozygosity between homologous chromosomes (Selmecki, Forche and Berman 2006; Ford *et al*. 2014; Hirakawa *et al*. 2015) and a high degree of genome plasticity driven by ploidy changes, karyotypic variations due to partial and whole chromosome aneuploidies, point mutations, short and long-range loss of heterozygosity (LOH) events and copy number variations (Chibana, Beckerman and Magee 2000; Selmecki, Forche and Berman 2006; Ford *et al*. 2014; Hirakawa *et al*. 2015; Ropars *et al*. 2018; Sitterlé *et al*. 2019). Furthermore, haploid and tetraploid strains have been observed both *in vitro* and *in vivo* (Hull, Raisner and Johnson 2000; Magee and Magee 2000; Hickman *et al*. 2013).

Multilocus sequence typing (MLST) and genome sequencing studies have revealed that *C. albicans* isolates are distributed amongst at least 23 genetic clusters (1–18, A-E) (Bougnoux *et al*. 2006; Odds *et al*. 2007; Odds 2010; Ropars *et al*. 2018). In general, there are no clear phenotypic associations with these clusters (Bougnoux *et al*. 2006; MacCallum *et al*. 2009). However, some clusters do exhibit geographical enrichment (Odds *et al*. 2007; MacCallum *et al*. 2009; Shin *et al*. 2011), suggesting independent recent evolutionary histories for these clusters. Cluster 13 is somewhat exceptional in that it represents a highly clonal lineage of isolates that exhibit low heterozygosity (Ropars *et al*. 2018). Isolates in cluster 13 are distributed worldwide (Fakhim *et al*. 2020), despite being called *Candida africana* strains (Tietz *et al*. 2001). They are isolated predominantly from the genital niche and display unusual morphological and phenotypic features that include slow growth, an inability to produce chlamydospores and assimilate aminosugars, and decreased virulence (Tietz *et al*. 2001; Romeo, De Leo and Criseo 2011; Borman *et al*. 2013). In contrast to other *C. albicans* clusters, cluster 13 isolates harbour a unique pattern of single nucleotide polymorphisms (SNPs) and a significantly lower level of heterozygosity (Ropars *et al*. 2018). In addition, in cluster 13 isolates, genes important for morphogenesis and virulence have undergone pseudogenisation, which probably explains the decreased virulence and apparent genital niche restriction of these isolates (Ropars *et al*. 2018).

Once thought to be an asexual obligate diploid organism, *C. albicans* has been shown to undergo a parasexual cycle (Magee and Magee 2000; Bennett and Johnson 2003; Ene and Bennett 2014). The majority of *C. albicans* diploid strains are incapable of mating, being heterozygous at the mating type-like (*MTL*) locus. However, mating can occur mainly between strains that have become homozygous at the *MTL* locus on chromosome 5, and have complementary *MTL* genotypes (i.e. are *MTLa/a* and *MTLα/α*). Additionally, mating in *C. albicans* is also dependent on a phenotypic switch from the mainly sterile ‘white’ phenotype to the mating competent ‘opaque’ phenotype (Miller and Johnson 2002). Mating between competent isolates of opposite mating-type results in tetraploid cells. These can subsequently undergo concerted chromosome loss, which can restore the diploid state in a meiosis-independent manner (Bennett and Johnson 2003; Hickman *et al*. 2015). However, this process yields diverse intermediate aneuploid states (Hickman *et al*. 2015). Hence, this mode of parasexual reproduction provides a means of generating genetic and phenotypic diversity in *C. albicans* (Forche *et al*. 2008; Hickman *et al*. 2015). Indeed, recombination has been shown to occur three orders of magnitude more frequently during concerted chromosome loss than during mitosis (Anderson *et al*. 2019). Interestingly, recombination during concerted chromosome loss is highly dependent on two meiosis-specific genes, *SPO11* and *REC8* (Forche *et al*. 2008; Anderson *et al*. 2019). The involvement of meiosis-specific genes in concerted chromosome loss has led to the suggestion that this process ‘*blurs the boundaries*’ between meiosis and mitosis, and that this ‘*parameiosis*’ might provide insight into the evolution of meiosis (Anderson *et al*. 2019).

The view that the parasexual cycle rarely occurs in the host is supported by population genetics, which shows that *C. albicans* populations are predominantly clonal (Pujol *et al*. 1993; McManus and Coleman 2014). Nevertheless, the conservation of mating genes suggests that this process is associated with an evolutionary advantage. Furthermore, because the parasexual cycle is stimulated by environmental stress, it may be a diversity-enhancing process that enhances adaptation and survival under hostile conditions (Selmecki, Forche and Berman 2010; Zhang *et al*. 2015; Hirakawa *et al*. 2017; Popp *et al*. 2019). This idea is corroborated by evidence of recombination and gene flow in natural isolates, despite the largely clonal structures of *C. albicans* populations (Odds *et al*. 2007; Bougnoux *et al*. 2008; Zhang *et al*. 2015; Ropars *et al*. 2018). This could explain why *C. albicans* isolates maintain a high degree of genetic diversity despite their predominantly clonal reproduction.

The diversity of *C. albicans* populations has arisen partly through changes in ploidy and aneuploidy. These mechanisms have provided *C. albicans* with a means of evolving rapidly in response to environmental challenges (Selmecki, Forche and Berman 2006; Diogo *et al*. 2009; Bennett, Forche and Berman 2014). The association of genome rearrangements with antifungal resistance acquisition has been well documented, with genomes of antifungal-resistant strains often exhibiting copy number variations and chromosome aneuploidies (Selmecki, Forche and Berman 2010). Indeed, a striking example of segmental aneuploidy was reported in fluconazole resistant strains, consisting of an isochromosome composed of the two left arms of chromosome 5 (Selmecki, Forche and Berman 2006, Selmecki *et al*. 2008). Trisomy of chromosome 2 or R has also been reported to enhance antifungal drug resistance in *C. albicans* (Xingxing Li *et al*. 2015; Yang *et al*. 2019). Large-scale chromosome rearrangements occur in *C. albicans* as an adaptation mechanism in both oral and GI niches (Ene *et al*. 2018; Forche *et al*. 2018). Similar observations have been made in isolates collected from a single human individual (Sitterlé *et al*. 2019). Genome sequencing of clinical isolates from patients that received antifungal therapy revealed that eight of the 21 isolates underwent karyotypic changes, with the majority being trisomic for chromosome 4 or 7 (Hirakawa *et al*. 2015). However, a more recent study of 182 clinical isolates might suggest that both segmental and whole chromosome aneuploidies are relatively infrequent events (Ropars *et al*. 2018). Changes in ploidy are known to provide a selective advantage under stress conditions, but can confer long-term fitness defects when grown under nonselective conditions, as illustrated by decreased growth and virulence (Hickman *et al*. 2015, 2013; Hirakawa *et al*. 2015). Therefore, the extent to which these events are observed in the genomes of *C. albicans* isolates must reflect the frequency of these types of genetic event and the nature of the selective pressures that these isolates recently faced.

Diversity has also arisen through high rates of mutation at the nucleotide level (SNPs, insertions and deletions). *Candida albicans* isolates display high levels of natural heterozygosity, with one heterozygous SNP occurring per 200–300 bp on average (Jones *et al*. 2004; Butler *et al*. 2009; Hirakawa *et al*. 2015; Ropars *et al*. 2018). The levels of heterozygosity are influenced by large LOH events, which can affect all chromosomes and are common in *C. albicans* isolates. LOH events are significantly elevated under stress conditions, such as exposure to antifungal agents, heat or oxidative stress (Forche *et al*. 2011; Ropars *et al*. 2018). Rapid phenotypic and genetic changes have been observed in various infection and colonisation models as well as in clinical isolates (Forche, May and Magee 2005; Bougnoux *et al*. 2006, 2009; Cheng *et al*. 2007; Bougnoux *et al*. 2008; Diogo *et al*. 2009; Lüttich *et al*. 2013; Ene *et al*. 2018; Forche *et al*. 2018; Sitterlé *et al*. 2020). This microevolution is driven primarily by *de novo* base substitution and short-range LOH events (Ene *et al*. 2018), and can clearly impact the relationship between fungus and host (Wartenberg *et al*. 2014; Tso *et al*. 2018; Liang and Bennett 2019) as well as resistance to antifungal therapy (Coste *et al*. 2006; Ford *et al*. 2014).

## THE HOST

**Figure 5.**
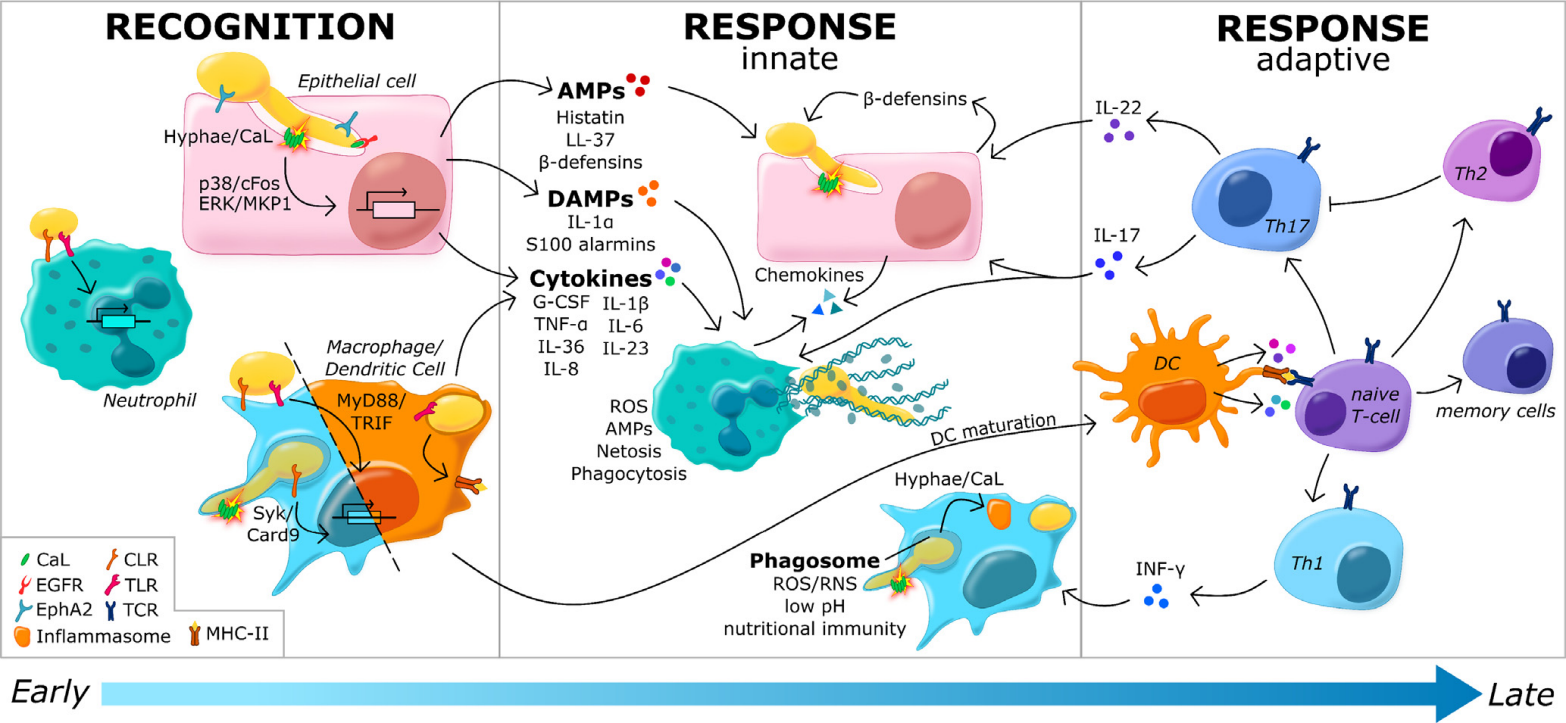
**Immune recognition of, and immune responses against, *C. albicans***. *Candida albicans* yeast and hyphal cells are recognised by neutrophils, macrophages and dendritic cells *via* pattern recognition receptors (see key). This recognition activates the expression and release of proinflammatory cytokines and chemokines that promote the recruitment of macrophages and neutrophils to the site of infection. Epithelial cells respond to hypha formation and the subsequent secretion of candidalysin by the fungus, by activating the expression and release of AMPs, DAMPs, chemokines and cytokines via p38/cFos and ERK/MKP1 signalling. The AMPs attenuate fungal growth and invasion, while DAMPs and cytokines promote inflammation. Myeloid cells promote fungal killing and clearance through a combination of phagocytosis and NETosis in the case of neutrophils. Fungal recognition leads to the maturation of dendritic cells, and their surface presentation of fungal antigens to naïve T-cells, which stimulates adaptive immunity. The interactions between antigen-presenting dendritic cells and naïve T-cells induces T-cell activation and differentiation into various effector T cell subsets that regulate mucosal immunity largely via IL-17 and IL-22 secretion, and stimulate macrophages via IFN-γ. See text.

Mammals are constantly exposed to microbes on the skin and mucosal surfaces of the GI, respiratory and reproductive tracts. Therefore, epithelial surfaces in the mucosal tissues represent primary sites of interaction between *C. albicans* and the host (Lim *et al*. 2012). To prevent microbial overgrowth on the epithelial barriers and microbial invasion of tissues, the host actively surveys and protects its barrier surfaces *via* two distinct, complementary and cooperating branches of the immune system: innate and adaptive immunity (Fig. 5). As well as forming a physical barrier, epithelial cells contribute to the host response through active recognition of microbes and evaluation of their pathogenic potential. This is complemented by myeloid cells of the innate immune system, which exploit evolutionarily conserved pattern recognition receptors (PRRs) to recognise microbial pathogen-associated molecular patterns (PAMPs). Recognition of PAMPs by PRRs triggers phagocytosis of the microbial target and/or antimicrobial effector responses with the purpose to eradicate the pathogen. In addition, the innate immune system, and dendritic cells (DCs) in particular, activate the adaptive immune system. T helper (Th) cells are activated in an antigen-specific manner to coordinate epithelial defenses, improve innate immune function, activate antibody responses, and ultimately control the fungal load and resolve inflammation. Through the development of immunological memory, adaptive immunity provides long-lasting protection against microbes. We address the cellular and molecular mechanisms of innate and adaptive immunity that provide critical protection against *C. albicans* infection at epithelial barriers where interactions between the fungus, host and microbiota play out. These interactions are dependent on tissue type and are influenced by variations between individuals that affect susceptibility to fungal infection.

### Innate immunity

#### Fungal recognition

The innate immune system is the first line of defense against *C. albicans* infection (Fig. 5). Epithelial cells (Richardson, Ho and Naglik 2018; Nikou *et al*. 2019; Swidergall 2019) combine with innate immune cells (Naglik *et al*. 2017; Verma, Gaffen and Swidergall 2017; Richardson *et al*. 2019) to provide this defense system, initiating anti-*Candida* immunity in response to fungal recognition.

Tissue-resident phagocytes, such as macrophages and DCs, are crucial in maintaining mucosal homeostasis (Ramirez-Ortiz and Means 2012; Xu and Shinohara 2017; Watanabe *et al*. 2019). However, innate immune cell populations differ between tissues, resulting in tissue-specific variation in the induction of innate and adaptive immune responses (see *Variability in the immune response*). Following hypha formation and *C. albicans* invasion, neutrophils and monocytes are rapidly recruited to the site of infection to mediate pathogen clearance through various antifungal responses (see *Antifungal response*) (Richardson *et al*. 2019).

Myeloid cells recognise specific microbial PAMPs using specific PRRs that fall into four main families: Toll-like receptors (TLRs), C-type lectin receptors (CLRs), nucleotide oligomerisation domain (NOD)-like receptors (NLRs) and RigI-helicase receptors (RLRs). CLRs are critical for fungal recognition (Hardison and Brown 2012). Several types of CLR recognise *C. albicans*, including Dectin-1, Dectin-2, Mincle, DC-Sign, and the mannose receptor (MR) (Hardison and Brown 2012; Dambuza *et al*. 2017; Goyal *et al*. 2018; Swidergall 2019). Dectin-1 recognises fungal β-glucans, which triggers the Card9-Syk pathway, leading to Nuclear Factor-kappa B (NFκB) activation and consequent cytokine and chemokine release (Drummond *et al*. 2011). In addition, dectin-1 induces phagocytosis and inflammasome activation (Kankkunen *et al*. 2010; Goodridge, Underhill and Touret 2012; Swidergall 2019). Dectin-2 recognises α-mannans (McGreal *et al*. 2006; Saijo *et al*. 2010) and induces the formation of Neutrophil Extracellular Traps (NETs) after recognising unopsonised *C. albicans* cells (Wu *et al*. 2019). In addition, Dectin-2 forms heterodimers with Dectin-3 and binds α-mannans on the surfaces of *C. albicans* hyphae (Zhu *et al*. 2013). Mannans are also recognised by Mincle, DC-Sign and the MR (Hardison and Brown 2012; Erwig and Gow 2016; Dambuza *et al*. 2017).

TLR-mediated PAMP recognition activates MyD88-dependent and TRIF signalling pathways in innate immune cells to regulate the inflammatory response (Kawasaki and Kawai 2014; Swidergall 2019). TLR2 and TLR4 recognise mannoproteins, while TLR9 recognises fungal DNA (Naglik *et al*. 2017). In addition, together with TLR9, the cytosolic NLR receptor NOD2 senses chitin particles (Wagener *et al*. 2014). NOD2-mediated recognition of chitin was found to down-regulate inflammatory responses (Wagener *et al*. 2014), which explains why NOD2 was initially described as being redundant for the induction of inflammatory responses against *C. albicans* (van der Graaf *et al*. 2006; van de Veerdonk *et al*. 2009). Recently, the epithelial Ephrin type-A receptor 2 (EphA2) was described as a non-classical PRR that recognises β-glucan (Swidergall *et al*. 2018). This receptor is expressed on neutrophils and stimulates antifungal activity during oropharyngeal candidiasis (OPC) (Swidergall, Solis *et al*. 2019). Meanwhile, the melanoma differentiation-associated factor 5 (MDA5), a member of the RIG-I-like receptor (RLR) family that senses viral RNA, has been reported to also trigger an antifungal immune response, although its ligand remains obscure (Jaeger, van der Lee *et al*. 2015) (Table 1).

**Table 1.** Pattern recognition receptors in epithelial and innate immune cells that recognise *C. albicans* pathogen-associated molecular patterns.

PRR family	PRR	Fungal PAMP	Expressed in	Reference
TLRs	TLR2	Phospholipomannans	Neutrophils, macrophages, DCs, Epithelial cells (oral, vaginal, intestinal)	(Kurt-Jones *et al*. 2002; Jouault *et al*. 2003; Fazeli, Bruce and Anumba 2005; Décanis, Savignac and Rouabhia 2009; McClure and Massari 2014)
	TLR4	O-linked mannans	Neutrophils, monocyte, macrophages, DCs, epithelial cells (oral, vaginal, intestinal)	(Netea *et al*. 2006; Hyung Sook Kim *et al*. 2016; Fazeli, Bruce and Anumba 2005; Weindl *et al*. 2007; McClure and Massari 2014)
	TLR9	Fungal DNA Chitin	DCs, Neutrophils, macrophages, epithelial cells (oral, vaginal, intestinal)	(Miyazato *et al*. 2009; Kasperkovitz *et al*. 2011; McClure and Massari 2014; Wagener *et al*. 2014)
CLRs	Dectin-1	β-glucans	Macrophages, monocytes, neutrophils, DCs, epithelial cells (oral, intestinal)	(Brown and Gordon 2001; Brown *et al*. 2002; Taylor *et al*. 2002; Ariizumi, Shen, Shikano, Xu *et al*. 2000; Cohen-Kedar *et al*. 2014)
	Dectin-2	Mannoproteins (a-mannans)	Macrophages, DCs	(Taylor *et al*. 2005; Ariizumi, Shen, Shikano, Ritter, *et al*. 2000)
	Dectin-3	Mannoproteins (a-mannans)	Macrophages,	(Zhu *et al*. 2013)
	DC SIGN	Mannoproteins	Macrophages, DCs	(Cambi *et al*. 2003; Rappocciolo *et al*. 2006)
	Mincle	Mannoproteins	Neutrophils, macrophages, DCs	(Wells *et al*. 2008; Vijayan *et al*. 2012; Martínez-López *et al*. 2019)
	MR	Mannoproteins Chitin	DCs, macrophages	(van de Veerdonk *et al*. 2009; Martinez-Pomares 2012; Wagener *et al*. 2014)
NA	EphA2	β-glucans	Oral epithelial cells, neutrophils	(Swidergall, Solis, *et al*. 2019)
	Galectin-3	β-mannosides	Monocytes, macrophages, DCs, neutrophils, epithelial cells	(Jouault *et al*. 2006)
RLRs	MDA5	Unknown	Monocytes, DCs, macrophages, epithelial cells	(Plato, Hardison and Brown 2015)
NLRs	NOD2	Chitin	Monocytes, DCs, macrophages	(Wagener *et al*. 2014)

PRRs involved in the recognition of *C. albicans* by myeloid cells have been well characterised (above), but less is known about epithelial cell PRRs that recognise *C. albicans*. Epithelial cells use several types of PRR to sense *C. albicans*, including TLR2, TLR4, dectin-1 and EphA2 (Weindl *et al*. 2007; Décanis, Savignac and Rouabhia 2009; Cohen-Kedar *et al*. 2014; Swidergall *et al*. 2018). Despite its primordial role in the recognition of *C. albicans* by myeloid cells, dectin-1 is thought to play a limited role in epithelial cells (Moyes *et al*. 2010; Verma *et al*. 2017; Richardson, Ho and Naglik 2018). Rather, sensing of fungal β-glucans by epithelial cells is achieved mainly by EphA2, which activates MAPK and STAT3 signalling to induce the secretion of inflammatory cytokines and antimicrobial peptides by oral epithelial cells (Swidergall *et al*. 2018). PRR expression patterns vary amongst epithelial cell types and this, together with differential myeloid cell types, contributes to niche-specific variations in mucosal responses against *C. albicans* (Nikou *et al*. 2019; Swidergall 2019) (see *Tissue-specific immune responses*).

Epithelial cells can be activated by the *C. albicans* peptide toxin, candidalysin, as well as through PRR-PAMP interactions. This cytolytic peptide damages epithelial cells and activates the epithelial growth factor receptor (EGFR) (Jemima Ho *et al*. 2019). This, in turn, activates p38/cFos and ERK/MKP1 signalling, leading to the initiation of various effector responses (see *Innate antifungal responses*). The epithelial response to candidalysin is particularly relevant to the transition of *C. albicans* from commensalism to pathogenicity, because candidalysin is synthesised during hyphal growth and accumulates in the invasion pocket as the fungus invades the epithelial surface (Moyes *et al*. 2016) (see *Invasion mechanisms*). This response to candidalysin endows epithelial cells with the ability to respond to the invasive hyphal form of *C. albicans*, rather than its relatively benign commensal state (Moyes *et al*. 2010; Naglik *et al*. 2017).

#### Innate antifungal responses

Following recognition of *C. albicans* by phagocytic receptors, phagocytes such as neutrophils and macrophages can engulf the target *C. albicans* cell by phagocytosis, the purpose being to entrap and kill the pathogen (Brown 2011) (Fig. 5). Phagocytosis involves rapid reorganisation of the plasma membrane and cytoskeleton, and the imposition of mechanical force to engulf the fungal cell and entrap it within a phagosome (Ostrowski, Grinstein and Freeman 2016; Huse 2017). The phagosome then undergoes a series of plasma-membrane phosphoinositide- and Rab-dependent membrane fusion and fission events with endolysosomal compartments that promote the assimilation of microbicidal and lytic enzymes, and the progressive acidification of the organelle, to form the mature phagolysosome (Brown 2011; Fairn and Grinstein 2012; Miramón, Kasper and Hube 2013; Erwig and Gow 2016; Walpole, Grinstein and Westman 2018). In an attempt to kill the fungus, the phagocyte exposes its fungal cargo to a low pH, a nutrient limiting microenvironment and a potent mix of proteases, reactive chemical species ROS and RNS, cation fluxes and AMPs (Lorenz, Bender and Fink 2004; Brown 2011; Miramón, Kasper and Hube 2013; Erwig and Gow 2016). However, these skirmishes between phagocyte and fungus do not always achieve fungal clearance. This is because *C. albicans* has evolved molecular mechanisms that help it to evade phagocytic recognition, escape the phagocyte following engulfment, and resist phagocytic killing mechanisms (Austermeier *et al*. 2020) (see *Fitness attributes* and *Immune evasion*).

PAMP-PRR interactions activate host cell signalling, which in turn, induces a myriad of effector responses that are specific to the cell and tissue type (Brown *et al*. 2002; Roeder *et al*. 2004). Epithelial cells secrete antimicrobial peptides (AMPs) such as LL-37, histatins and β-defensins. These AMPs exert their antifungal effects by a variety of mechanisms that include binding to the fungal cell wall or permeabilizing the fungal plasma membrane (Krishnakumari, Rangaraj and Nagaraj 2009; Chang *et al*. 2012; Swidergall and Ernst 2014). In the oral epithelium, nitric oxide and human-β-defensin (hBD)-2 production contribute to the early defensive response following direct contact with *C. albicans* and intra-epithelial invasion (Casaroto *et al*. 2019). In the GI tract, mucins produced by goblet cells suppress the yeast-hypha transition, surface adhesion and biofilm formation of *C. albicans*, thereby minimizing the capacity of the fungus to attach to, invade, and damage the epithelium (Kavanaugh *et al*. 2014) (see *Virulence factors*).

When *C. albicans* does manage to colonise the epithelium, the fungal toxin, candidalysin, plays a central role in triggering downstream responses (Kasper *et al*. 2018; Jemima Ho *et al*. 2019; Swidergall, Khalaji *et al*. 2019). The damage caused by candidalysin causes epithelial cells to passively alert professional immune cells through their release of danger-associated molecular patterns (DAMPs) or alarmins (Yang and Oppenheim 2009). For example, S100 alarmins produced by the vaginal epithelium are a potent driver of neutrophil influx during vaginitis in a murine model of infection (Yano *et al*. 2010, 2014). Similarly, damage to oral epithelial cells results in their release of the alarmin IL-1ɑ, which triggers the neutrophil response to *C. albicans* in the oral mucosa *via* IL-1 signalling (Dongari-Bagtzoglou, Kashleva and Villar 2004; Altmeier *et al*. 2016). Epithelial cells also produce pro-inflammatory cytokines such as IL-1β, IL-6, IL-8, G-CSF, TNF, and IL-36 (Villar *et al*. 2005; Verma *et al*. 2018). IL-8 acts as a chemoattractant that mobilises neutrophils from the circulation to the infection site. These neutrophils engage the fungus directly. They also engage in cross talk with local epithelial cells *via* TNF, thereby promoting TLR4-mediated signalling in the epithelium to enhance protection against fungal invasion and cell damage during oral candidiasis (Weindl *et al*. 2007).

Neutrophils are central players in antifungal defences due to their rapid activation of the fungicidal oxidative burst, (Peltroche-Llacsahuanga *et al*. 2000), their formation of NETs (Kenno *et al*. 2016), and their release of AMPs *via* degranulation (Urban *et al*. 2009) (Fig 5). Mice with *C. albicans* colonisation in their GI tract display enhanced neutrophil responsiveness and fungus-specific CD4 + T-cell responses during systemic candidiasis (Shao *et al*. 2019). This contrasts with observations during VVC in humans and mice, where fungal susceptibility is associated with uncontrolled inflammation and neutrophil influx (Black *et al*. 1998; Fidel *et al*. 2004). These observations reinforce the context-dependent nature of local immune responses.

Macrophages contribute to fungal clearance through their uptake of fungal cells, displaying a greater phagocytic capacity, but lower uptake rate, than polymorphonuclear leukocytes, (PMNs) (Rudkin *et al*. 2013) (Fig. 5). The hyphal form of *C. albicans* is relatively resistant to phagocytosis (Lewis *et al*. 2012). Nevertheless, macrophages still engulf portions of the hyphae, which can become trapped in ‘frustrated phagosomes’ (Maxson *et al*. 2018). After phagocytosis by macrophages, *C. albicans* yeast cells can undergo morphogenesis to generate hyphae. The yeast-hypha transition activates the NOD-and pyrin domain-containing protein 3 (NLRP3) inflammasome. This is essential for the release by the macrophage of pro-inflammatory IL-18 and IL-1β, which further promote Th1/Th17 activity during infection (Joly *et al*. 2009; van de Veerdonk, Joosten *et al*. 2011; Kasper *et al*. 2018). However, hyphal development within the phagolysosome can help *C. albicans* evade macrophage killing by inducing pyroptosis, rupture and death of the macrophage *in vitro* (Vázquez-Torres and Balish 1997; Uwamahoro *et al*. 2014; Wellington *et al*. 2014; Kasper *et al*. 2018; O'Meara *et al*. 2018; Westman *et al*. 2018; Austermeier *et al*. 2020). Nevertheless, macrophages provide an important contribution to antifungal defences during systemic infection. For example, the functionality of resident renal macrophages, which is dependent on expression of the chemokine receptor CX3CR1, is important for controlling *C. albicans* in the early stages of a systemic infection, and for survival of the host (Lionakis *et al*. 2013). Similarly, microglia play an important role in antifungal immunity in the central nervous system, promoting neutrophil recruitment *via* candidalysin induced IL-1β and CXCL1 signalling (Drummond *et al*. 2019).

Mast cells modulate the antifungal potency of macrophages. Activated mast cells enhance macrophage functionality by improving their crawling ability and chemotaxis in response to *C. albicans* stimulation (De Zuani *et al*. 2018). Meanwhile, resting mast cells inhibit the phagocytosis of *C. albicans* by macrophages, which suggests a role for mast cells in the maintenance of commensalism (De Zuani *et al*. 2018). Inflammatory monocytes expressing CCR2 and Ly6C also contribute to fungal clearance during the early stages of systemic infection. Clearance is enhanced in the kidney and brain, but less so in the liver and spleen, indicating an organ-specific role for these monocytes during disseminated infection (Ngo *et al*. 2014).

#### Fungal countermeasures

During co-evolution of fungus and host, the antifungal responses of the immune system have imposed strong selective pressures upon *C. albicans* to evade these responses. Consequently, the fitness of the fungus *in vivo* has been enhanced by the development of a variety of fungal countermeasures that promote immune evasion and manipulation (Underhill 2007; Marcos *et al*. 2016).

A number of the fitness attributes and virulence factors, described above, promote immune evasion (see *Virulence factors* and *Fitness attributes*). For example, the formation of biofilms shields *C. albicans* cells from immunological attack (Kernien *et al*. 2017). The ability of *C. albicans* to resist pH extremes and to actively resist phagolysosomal acidification reduces the antifungal potency of phagocytes (Vylkova *et al*. 2011; Bain, Gow and Erwig 2015; Vylkova and Lorenz 2017; Westman *et al*. 2018). Also, the activation of robust oxidative and nitrosative stress responses provides a degree of protection against the toxic ROS and RNS generated by innate immune cells (Miramón *et al*. 2012). These responses include secreted and cell wall bound ROS detoxifying enzymes that help to counter immune attack (Crowe *et al*. 2003; Fradin *et al*. 2005; Dantas *et al*. 2015). However, *C. albicans* is sensitive to certain combinations of stress encountered within the phagosome (Kaloriti *et al*. 2014; Kos *et al*. 2016).

Hypha formation reduces the exposure of *C. albicans* to phagocytic killing because lengthy hyphal cells are harder to engulf, and hyphae have been reported to display lower levels of the inflammatory MAMP, β-1,3-glucan, at their cell surface (Gantner, Simmons and Underhill 2005; Bain *et al*. 2014; Mukaremera *et al*. 2017). Furthermore, *C. albicans* can undergo yeast-hypha morphogenesis following phagocytosis by macrophages, rupturing the phagosome and eventually leading to host cell death and fungal escape (Lewis *et al*. 2012; Ermert *et al*. 2013; Vylkova and Lorenz 2017). Indeed, the fungus is capable of triggering pyroptosis, inflammasome activation and cell death in a macrophage that has engulfed it (Uwamahoro *et al*. 2014; Wellington *et al*. 2014; O'Meara *et al*. 2015; Kasper *et al*. 2018), and can also induce host cell death through metabolic competition for glucose (Tucey *et al*. 2018; Tucey *et al*. 2020). Like other fungal pathogens, *C. albicans* may also escape the macrophage without lysing the host cell (Bain *et al*. 2012), although this mode of escape is thought to be rare.

Members of the secreted aspartic protease family (Sap1-3) promote immune evasion by degrading complement proteins (C3b, C4b, C5) thereby reducing the inhibitory potential of the complement system (Gropp *et al*. 2009). *Candida albicans* also expresses complement binding proteins at its cell surface that reduce the efficacy of the complement system (Poltermann *et al*. 2007; Zipfel and Skerka 2009). Pra1, which promotes zinc assimilation in *C. albicans* (see *Fitness attributes*), also interacts with complement regulators and plasminogen. In addition, Pra1 was the first protein described to bind to C4BP, which regulates the classical and lectin complement pathways and avoids C3b and C4b deposition on the fungal surface when captured by *C. albicans*, impeding complement cascade progression (Luo et al. 2009, 2011; Zipfel, Hallström and Riesbeck 2013). Furthermore, *C. albicans* secretes prostaglandins that modulate host immunity by downregulating chemokine and TNF production (Noverr *et al*. 2001). On the other hand, host immune mediators such as IFNγ, IL-17, TNF and PGE_2_ influence *C. albicans* growth, filamentation and biofilm formation (Kalo-Klein and Witkin 1990; Noverr and Huffnagle 2004; Zelante *et al*. 2012; Rocha *et al*. 2017).

More recently, it was found that *C. albicans* yeast cells can evade phagocytic recognition by actively masking β-1,3-glucan at their cell surface. Interestingly the fungus exploits host signals, such as lactate, hypoxia, iron limitation and ambient pH, to modulate its β-1,3-glucan exposure (Ballou *et al*. 2016; Sherrington *et al*. 2017; Lopes *et al*. 2018; Pradhan *et al*. 2018; Cottier *et al*. 2019; Pradhan *et al*. 2019). Reducing the levels of β-1,3-glucan exposure leads to the attenuation of anti-*Candida* immune responses (Ballou *et al*. 2016; Sherrington *et al*. 2017; Lopes *et al*. 2018; Pradhan et al. 2018, 2019) and promotes disease progression (Lopes *et al*. 2018). Indeed, the fungus appears to use these host signals to anticipate impending immune attack and to protect itself by activating immune evasion mechanisms (Alistair J P Brown *et al*. 2019). These, and other anticipatory responses (Rodaki *et al*. 2009; Brunke and Hube 2014), provide strong evidence for the co-evolution of *C. albicans* with its host (Brown, Larcombe and Pradhan 2020).

### Adaptive immunity

The adaptive immune system evolved to establish long-term protection through its ability to generate immunological memory (Fig. 5). The key role played by this arm of the immune system in providing surveillance of commensal organisms is reflected in the fungal dysbiosis that occurs in the absence of adaptive immunity (Lanternier, Cypowyj *et al*. 2013). The adaptive immune system involves B and T cells. B cells are essential for the production of antibodies, whereas T helper (Th) cells provide essential support for mucosal host defense and the innate immune response.

*Candida albicans*-specific antibodies are detectable in individuals that have been exposed to the fungus (Swoboda *et al*. 1993; López-Ribot *et al*. 2004; Pitarch *et al*. 2006). Their role in the control of fungal colonisation remains unclear, although the presence of anti-*C*. *albicans* antibodies might provide protection to mice against a potentially lethal systemic challenge (Matthews *et al*. 1991), as does gut colonisation through the development of pronounced anti-*C. albicans* IgG levels (Huertas *et al*. 2017). For some time, it has been thought that antibodies may have diagnostic as well as therapeutic value (Matthews *et al*. 1988). Recent studies have reinforced their diagnostic potential (Wang *et al*. 2020), and recombinant anti-*C. albicans* antibodies have been shown to display therapeutic potential in preclinical models of infection by improving phagocytosis (Rudkin *et al*. 2018).

T cells exist as various subtypes that contribute differentially to antifungal immunity (Borghi *et al*. 2014; Verma *et al*. 2014; Lionakis and Levitz 2018) (Fig. 5). Among CD4^+^ T cells, Th1 and Th17 cells promote the phagocytic clearance of fungal cells through the release of inflammatory cytokines such as IFN-γ and IL-17A/F, respectively, and these T cell subsets are critical for protective antifungal immunity. On the other hand, Th2 cells counter-regulate Th1 and Th17 responses, which can favour fungal persistence and promote allergic manifestations. Regulatory T cells (Tregs) maintain the homeostatic balance between these responses and limit inflammation as the infection is cleared. Th17 cells represent a major subset, and Th1 and Th2 cells minor subsets, of the human *C. albicans*-specific T helper cell population (Becattini *et al*. 2015; Bacher *et al*. 2019). However, additional T helper cell subsets have been described more recently (Eyerich *et al*. 2011; Becker *et al*. 2016). Moreover, T helper cells express plasticity. For example, *C. albicans-*specific Th17 cells can adopt the ability to produce additional cytokines, such as the Th1 prototypic cytokine IFN-γ (Zielinski *et al*. 2012). Cytotoxic (CD8^+^) T-cells may also play a role in anti-*Candida* immunity (Beno, Stöver and Mathews 1995; Marquis *et al*. 2006).

The major protective role of Th17 cells in antifungal immunity is illustrated by the strong association of human defects in this T cell compartment and IL-17 signalling with uncontrolled fungal growth on mucosal surfaces and the skin (Puel *et al*. 2011; Ling *et al*. 2015; Li *et al*. 2017; Puel 2020). Consistently, mice with defects in the IL-17 signalling pathway display a reduced ability to cope with *C. albicans* administered *via* oropharyngeal or epicutaneous routes (Conti *et al*. 2009; Gladiator *et al*. 2013; Kashem, Igyarto *et al*. 2015), while IFN-γ-producing Th1 cells may have a disease-promoting effect (Igyártó *et al*. 2011). Also, the expansion of fungus-specific Th1 and Th17 cells in response to mucosal colonisation enhances protection against subsequent systemic *C. albicans* infections in mice (Romani *et al*. 1994; Shao *et al*. 2019). However, T cell- and IL-17-defects do not alter susceptibility to systemic infection in humans (Lionakis 2014).

CD4 + T cells are characterised by their ability to respond in an antigen-specific manner. Antigen-specific activation of (naïve) T cells depends on their interactions with DCs that present antigen on MHC-II molecules, and provide co-stimulatory and polarising cytokine signals. DCs are divisible into several subsets, most of which reside in peripheral tissues in close proximity to the microbiota where they interact with *C. albicans*. In response to PRR-mediated activation, DCs undergo a maturation program and migrate to the draining lymph nodes, where they encounter, activate, and prime antigen-specific T cells. This process relies on a tightly coordinated interplay between the innate and adaptive immune system (Fig. 5). The priming of T cells comprises of three steps. First, the recognition of peptide-MHC-II complexes by T cells *via* their T cell receptor (TCR) defines the antigen-specificity of the response. Second, this interaction is supported by adhesion and co-stimulatory molecules, which are induced at the cell surface of DCs in response to microbial stimulation, and these form an immune synapse that stimulates T cell proliferation. Third, the cytokine microenvironment directs the T cell differentiation towards distinct Th lineages *via* STAT (signal transducer and activator of transcription) signalling and the induction of fate-determining transcription factors (Wüthrich, Deepe and Klein 2012).

While antigens and the polarisation-inducing microbial signals are functionally distinct, the physical connection between antigen and PAMP enhances the efficiency of the T cell activation and differentiation process. Some of the few naturally processed and presented *C. albicans* antigens identified so far are glycosylated cell wall proteins, such as Mp65 (Pietrella *et al*. 2008) and Als3 (Bär *et al*. 2012). These mannoproteins can therefore serve concomitantly as a source of MHC-II antigen cargo as well as PAMPs. Such antigens support the coordinated process of antigen presentation and T cell polarisation.

The process of DC maturation is shaped by the specific PRR pathways that become activated in DCs following a microbial encounter (Fig. 5). This then determines the profile of cytokines that are produced, and hence directs the fate of the Th cell polarisation. Fungal cell wall components such as mannans and β-1,3-glucans trigger Syk- and CARD9-dependent cytokine signatures characterised by IL-23, IL-6, and IL-1β, which collectively instruct Th17 differentiation (LeibundGut-Landmann *et al*. 2007; Robinson *et al*. 2009; Saijo *et al*. 2010). IL-6 and IL-1β, together with TGF-β in mice, drive the commitment of Th17 cells, while IL-23 promotes lineage maintenance in a STAT3- and RORγt-dependent manner (Korn *et al*. 2009). Th17 cell differentiation is further modulated by the antigen dose and by tissue-specific cues.

Th17 cells produce the IL-17 family of effector cytokines: IL-17A and IL-17F as well as IL-22. These cytokines act primarily on epithelial cells and control the expression of genes linked to antimicrobial defense and tissue repair (Conti et al. 2009, 2016). IL-17 can also play an important role in promoting neutrophil recruitment (Liang *et al*. 2007), although, in the oral mucosa, the neutrophil response against *C. albicans* is largely independent of IL-17 (K Trautwein-Weidner *et al*. 2015). Instead, it depends on IL-1 and chemokines produced by epithelial cells in response to virulent *C. albicans* strains (Altmeier *et al*. 2016). While the functions of IL-17A and IL-17F are related, they do play non-redundant roles in host defense (Gladiator *et al*. 2013; Whibley *et al*. 2016). Similar to IL-17A and IL-17F, IL-22 also induces AMPs and contributes to fungal control (Liang *et al*. 2006). However, in contrast to IL-17A and IL-17F, defects in the IL-22 pathway have a minor impact on fungal control in experimentally infected mice (Conti *et al*. 2009; De Luca *et al*. 2013). Lately, IL-22 and IL-17A/F have been found to function nonredundantly during OPC, and IL-22 was shown to regulate the responsiveness of the epithelium to IL-17 (Aggor *et al*. 2020).

CD4 + T cells are the major source of IL-17 during responses to *C. albicans* at barrier tissues, but other sources may also contribute. CD8+ αβ T cells can produce IL-17 in response to *C. albicans* (and other fungi), and these cells may play a compensatory role in the absence of CD4 + T cells (Nanjappa *et al*. 2012; Hernández-Santos *et al*. 2013). Moreover, innate lymphocytes and innate lymphoid cells (ILCs) can generate IL-17 (Cua and Tato 2010; Gladiator *et al*. 2013). In experimental models of oral infection, where naïve mice were exposed to a virulent *C. albican*s strain, the antifungal response was characterised by induction of IL-17 in ILCs, γδ T cells and a tissue-resident population of αβ T cells that respond in a TCR-independent manner (Sparber *et al*. 2018; Conti, Peterson *et al*. 2014; Kashem, Riedl *et al*. 2015; Verma *et al*. 2017). These three cellular subsets act in a partially redundant manner (Conti, Peterson *et al*. 2014; Gladiator *et al*. 2013). Therefore, although small in size, the IL-17-producing ILC population can compensate for the absence of αβ and γδ T cells during acute OPC (Gladiator *et al*. 2013). The extent to which innate sources of IL-17 contribute to antifungal defense in humans to maintain host-fungus homeostasis is not yet clear.

As a result of their exposure to *C. albicans* in the microbiota, most humans produce *C. albicans*-specific memory Th17 cells. In the circulation, these cells display the phenotype of effector memory T cells, which can respond rapidly to fungal exposure (Acosta-Rodriguez *et al*. 2007). In the skin, their expression of CD69 and CD103 characterises these *C. albicans*-specific memory Th17 cells as tissue-resident memory cells (Park *et al*. 2018). The maintenance of *C. albicans*-specific T cells is dependent on the persistence of the fungus in the host (Park *et al*. 2018; Shao *et al*. 2019; Kirchner and LeibundGut-Landmann 2020). The relevance of tissue-resident memory T cells for local immunosurveillance of *C. albicans* in barrier tissues was confirmed recently in a model of stable *C. albicans* commensalism, where tissue-resident memory T cells were sufficient to prevent fungal overgrowth (Kirchner and LeibundGut-Landmann 2020). The relationship between circulating and tissue-resident memory T cells directed against *C. albicans* remains to be determined, although their shared T cell receptor sequences suggest a common origin for both populations of memory Th17 cells (Park *et al*. 2018). Clearly, IL-17 immunity plays an important protective role in antifungal immunity. However, IL-17 signalling also has pathogenic potential, such as in the context of some autoimmune disorders (Eyerich, Dimartino and Cavani 2017) (see *Immunopathology in candidiasis*).

FoxP3 + IL-2Rα(CD25+) regulatory T cells (Tregs) are key mediators of immune regulation that provide endogenous regulatory mechanisms that can prevent potentially harmful immune responses. These Tregs confer immune tolerance through the expression of IL-10 and TGF-β, the consumption of IL-2, and the expression of inhibitory receptors that target T cells directly or indirectly *via* modulation of DC functionality (Romano *et al*. 2019). Furthermore, Tregs are developmentally linked to Th17 as they can promote Th17 differentiation by consumption of IL-2 (a cytokine that constrains Th17 differentiation) and, in mice, by providing TGF-β (which promotes Th17 polarisation) (Pandiyan *et al*. 2011). While Tregs directed against *C. albicans* are largely expanded in the physiological T cell repertoire in humans (Bacher *et al*. 2014), their contribution to the maintenance of stable *C. albicans* homeostasis in barrier tissues remains unclear. In a murine model of *C. albicans* commensalism, Tregs were dispensable for stable fungal colonisation and an absence of Tregs did not result in dysregulation of the antifungal Th17 response (Kirchner *et al*. 2019). Instead, the kynurenine pathway, which regulates tryptophan catabolism, might contribute to antifungal tolerance and limit inflammation in mucosal tissues (De Luca *et al*. 2013).

To summarise, a combination of epidemiological data, association studies in human primary immunodeficiency (PID) syndromes, *in vitro* challenges with primary human cells, and experiments in various mouse models of superficial candidiasis, have highlighted the importance of Th17 immunity during long-term colonisation of barrier tissues by *C. albicans*, and the fine lines between fungal commensalism and pathogenicity, and health and disease.

### Tissue-specific variability of the mucosal immune response

*Candida albicans* colonises and causes infections in a range of different tissues, each of which characterised by a different architecture, nutrient supply, metabolic environment, and immune cell composition. Consequently, distinct host defense mechanisms against *C. albicans* exist in each tissue. Adaptive T cell immunity predominates in fungal control at the skin and most mucosal barriers (except for the vaginal mucosa), whereas innate myeloid cell-mediated mechanisms dominate the immune response to systemic infection (Lionakis 2014). Neutrophils and inflammatory monocytes have also been linked to antifungal immunity in barrier tissues. This notion has arisen primarily from experiments involving acute infections of previously *C. albicans*-naïve mice with highly virulent *C. albicans* strains, which trigger a strong inflammatory response and tissue damage. Under such conditions, inflammatory leukocytes (primarily neutrophils) are rapidly recruited to the infected tissues (Conti *et al*. 2009; K Trautwein-Weidner *et al*. 2015; Bai *et al*. 2020) where they prevent deep tissue invasion and mediate the rapid elimination of *C. albicans* (K Trautwein-Weidner *et al*. 2015). In contrast, *C. albicans* colonisation of barrier tissues is not generally accompanied by tissue inflammation (Schönherr *et al*. 2017), just as fungal commensalism in healthy individuals is not associated with inflammation.

In the vaginal mucosa, pathogenesis is thought to arise largely as a consequence of neutrophil-mediated immunopathology rather than a defect in T cell immunity (Fidel *et al*. 2004; Giraldo *et al*. 2012; Rosati, Bruno, Jaeger, Kullberg *et al*. 2020). Symptomatic infection correlates with elevated infiltration of neutrophils that are not able to limit the fungal burden (Yano, Noverr and Fidel 2017; Ardizzoni *et al*. 2020).

In contrast to the vaginal mucosa, where Th17 cells do not provide a major protective contribution, Th17 immunity is crucial for controlling the commensal colonisation of *C. albicans* on the skin and the mucosa of the oral cavity and GI tract (Sparber and LeibundGut-Landmann 2019). Experiments in mice have shown that the mechanisms of Th17 induction vary depending on the tissue. This is probably due to differences in the composition of antigen-presenting cells between the different tissues. Langerhans cells (LCs) are the predominant DC subset in the skin epidermis, but this cell type only represents a fraction of the DC population in the oral and vaginal epithelium (Hovav 2018). In the skin, LCs prime *C. albicans*-specific Th17 cells (Kashem, Igyarto *et al*. 2015), but they appear dispensable in the oral mucosa where conventional migratory DCs and monocyte-derived inflammatory DCs execute this task (Kerstin Trautwein-Weidner *et al*. 2015). In the gut, CX3CR1 + mononuclear phagocytes are essential for the initiation of adaptive immunity against *C. albicans* (Leonardi *et al*. 2018). Meanwhile, in the vaginal mucosa, plasmacytoid DCs may dominate and instruct a primarily tolerogenic response (LeBlanc, Barousse and Fidel 2006). Therefore, DCs are central coordinators of antifungal immunity. This relates not only to T cell activation in barrier tissues, but also to systemic candidiasis where DCs are indispensable for organising neutrophil-mediated innate immunity (Whitney *et al*. 2014).

**Explanatory Box 1: Immunopathology**

Neutrophils are amongst the first immune cells to be recruited from the bloodstream to the site of infection or tissue injury. Their recruitment is a multi-step process initiated by changes in the endothelium, and is induced by inflammatory mediators secreted by epithelial and tissue-resident immune cells (Kolaczkowska and Kubes 2013). At the site of infection, neutrophils clear pathogens through a combination of mechanisms including phagocytosis, degranulation, and NET formation (Selders *et al*. 2017; Rosales 2018). However, the secretion of ROS, proteolytic enzymes and AMPs by neutrophils can also lead to tissue injury and collateral damage (Wang 2018). Neutrophils die during the process of NETosis and release their nuclear and cytoplasmic contents. This can result in the presentation of auto-antigens and the production of pro-inflammatory cytokines, DAMPs and alarmins (Wang 2018; Wilgus 2018). DAMPs induce further neutrophil recruitment (Pittman and Kubes 2013), promoting a hyperinflammatory loop that, if not dampened by anti-inflammatory mechanisms, can exaggerate inflammation and tissue damage (Tisoncik *et al*. 2012). The adaptive immune system also mediates immunopathology *via* T cells, and Th17 cells in particular. *C. albicans*-specific Th17 cells promote inflammation and mediate immunopathological effects (Bacher *et al*. 2019; Shao *et al*. 2019; Hurabielle *et al*. 2020). Key anti-inflammatory mechanisms are mediated by Treg cells, myeloid suppressor cells, and anti-inflammatory molecules such as IL-1-family cytokines (IL-1Ra, IL-37, IL-38, IL-36Ra), IL-10, α1-antitrypsin, soluble cytokine receptors, and cytokine binding proteins (Netea *et al*. 2017; Dinarello 2018). The resolution of inflammation is an active process comprising of numerous signalling pathways that inhibit the inflammatory loop and limit tissue injury, as well as promoting pathogen clearance (Netea *et al*. 2017).

### Immunopathology in candidiasis

The innate and adaptive immune responses provide essential protection against mucosal and life-threatening systemic infections, but uncontrolled inflammation can contribute to disease by causing immunopathology (Explanatory Box 1). There is a balance between immune protection and immunopathology. Using mouse models of systemic candidiasis, some investigators found that type I interferons promote fatal immunopathology through the recruitment and activation of inflammatory monocytes and neutrophils (Majer *et al*. 2012), whereas others observed reduced survival and concluded that type I interferons are crucial for immunity against *C. albicans* (del Fresno *et al*. 2013). Neutrophil accumulation in the kidneys and lung has been shown to cause immunopathology and organ failure in murine models (Lionakis *et al*. 2011; Desai and Lionakis 2018; Lee *et al*. 2018). During VVC in mice and humans, candidalysin-induced mucosal damage allows DAMPs and proinflammatory cytokine secretion, which promotes neutrophil recruitment and the exacerbation of inflammation (Richardson *et al*. 2018). Moreover, activation of the NLRP3 inflammasome and unrestrained IL-1β production can induce a hyperinflammatory state at the vaginal mucosa and acute symptoms of VVC (Rosati, Bruno, Jaeger, Ten Oever *et al*. 2020). This is influenced by endogenous anti-inflammatory mediators and environmental conditions (Rosati, Bruno, Jaeger, Ten Oever *et al*. 2020) such as short-chain fatty acids (SCFAs) derived from resident bacteria, which also play a crucial role in the immunopathology of oral candidiasis in mice (Bhaskaran *et al*. 2018). Th17 polarisation associated with intestinal *C. albicans* colonisation can be deemed as protective as it can cross-protect against systemic disease (Shao *et al*. 2019). However, these specific Th17 cells also contribute to allergic airway inflammation (Bacher *et al*. 2019; Shao *et al*. 2019) through cross-reactivity to the lung pathogen *Aspergillus fumigatus* (Bacher *et al*. 2019). *C. albicans*-specific Th17 cells can also promote inflammation in the skin and thereby contribute to psoriaform pathology (Hurabielle *et al*. 2020).

Several endogenous mechanisms regulate inflammation to maintain the balance between immune protection and immunopathology (Netea *et al*. 2017). The neutrophil response protects against *C. albicans* by inducing neutrophil chemokines (Mengesha and Conti 2017; Sparber and LeibundGut-Landmann 2019), but these also promote inflammation. The IL-1 family of cytokines, which drive neutrophil responses (Altmeier *et al*. 2016; Verma *et al*. 2018), are regulated by endogenous anti-inflammatory cytokines. For example, IL-37 compromises protection against systemic infection by reducing neutrophil influx (van de Veerdonk *et al*. 2014), but the capacity to reduce this influx potentially makes IL-37 a key player for preventing immunopathology. Other endogenous regulators include IL-1Ra, which neutralises IL-1 signalling and dampens NLRP3 Inflammasome activity, thereby contributing to reduced immunopathology (Borghi *et al*. 2015). The anti-inflammatory cytokines IL-36Ra and IL-38 can also attenuate the *C. albicans*-induced Th17 response (van de Veerdonk *et al*. 2012).

Clearly, molecules that target the IL-17 and IL-1 signalling pathways may have potential therapeutic value as treatments for immunopathology associated with candidiasis. Targeting the NLRP3 inflammasome has also been suggested as a potential strategy to ameliorate inflammation during VVC (Bruno *et al*. 2015; Richardson *et al*. 2018). However, the fine balance between protection and pathology must be deciphered before the accurate therapeutic modulation of these pathways can be achieved. Furthermore, the role and therapeutic applications of immunomodulators such as Indoleamine-pyrrole 2,3-dioxygenase 1 (IDO1), an enzyme producing tolerogenic kynurenines (De Luca *et al*. 2013), should be further evaluated.

### Trained Immunity

The classical paradigm of host immune defense is based on the ability of the innate immune system to provide short term protection, combined with the capacity of adaptive immunity to mount immunological memory and provide long-lasting protection against the same pathogen. A growing body of evidence now shows that the innate immune system is able to generate immunological memory, independently of adaptive immunity. This phenomenon, which is termed *‘trained immunity’*, has been described in invertebrates, plants, and mammals (Kurtz and Franz 2003; Durrant and Dong 2004; Netea, Quintin and van der Meer 2011), and is based on functional reprogramming of innate immune cells.

*Candida albicans*, and individual components of its cell wall, are potent immune modulators (see *Cell wall*). Even in mice that are deficient in T and B lymphocytes (i.e. lack adaptive immunity), an initial non-lethal exposure to *C. albicans* provides protection against a subsequent *C. albicans* infection (Bistoni *et al*. 1986). This resistance to re-infection was described as a macrophage-dependent mechanism associated with enhanced production of the proinflammatory cytokines TNF, IFN-γ, and IL-1β (Vecchiarelli *et al*. 1989). Moreover, protection was not restricted to disseminated candidiasis: cross-protection to unrelated pathogens such as *Staphylococcus aureus* was also induced (Bistoni *et al*. 1986; Netea, Quintin and van der Meer 2011). Further studies demonstrated that stimulation with *C. albicans* or β-glucan, leading to activation of the dectin-1/PI3K-Akt-mTOR axis (Quintin *et al*. 2012; Cheng *et al*. 2014), elicits epigenetic remodeling of the transcriptional repertoire (Saeed *et al*. 2014). This leads to a shift in immune cell metabolism from oxidative phosphorylation to aerobic glycolysis (the Warburg effect) (Cheng *et al*. 2014), and enhanced pro-inflammatory cytokine production (Quintin 2019; Netea *et al*. 2020). Further studies revealed that β-glucan-primed monocytes differentiate into macrophages that display highly active metabolic activity and increased glucose consumption (Leonhardt *et al*. 2018). Interestingly, *C. albicans*-induced trained immunity is defective in chronic mucocutaneous candidiasis (CMC) patients, indicating that STAT-1 signalling is involved in the induction of trained immunity (Ifrim *et al*. 2015).

The induction of trained immunity depends strongly on the nature of the ligand and the PRR that is activated. For example, while TLR4 activation by lipopolysaccharide (LPS) can lead to a state of immunotolerance or immunoparalysis that compromises antifungal host defense (Grondman *et al*. 2019), the TLR4 agonist, monophosphoryl lipid A (MPLA), has been recently reported as an inducer of trained immunity (Fensterheim *et al*. 2018). Immunotolerance in sepsis patients increases the risk of secondary infections, including candidiasis (Otto *et al*. 2011). Conversely, trained immunity induced by *C. albicans* can enhance protection against sepsis in mice (Cheng *et al*. 2014). In addition, *C. albicans* colonisation of the GI tract provides protection against a variety of systemic pathogens (Tso *et al*. 2018). Therefore, the temporary transcriptional and metabolic rewiring *via* β-glucan-administration might provide a strategy to revert the LPS-induced tolerance of innate immune cells. Indeed, pharmacological targeting in myeloid cells, for example, by inhibition of the phosphatase SHIP-1 (Saz-Leal *et al*. 2018) or the IRG1-itaconate-SDH axis (Domínguez-Andrés *et al*. 2019), could play a pivotal role in harnessing beneficial effects of trained immunity (Mulder *et al*. 2019).

### Variability amongst individuals

Variation between individuals influences the host-fungus interaction and susceptibility to fungal infection. The identification of candidate genetic traits is, therefore, pivotal for the selection of patients that would benefit from host-directed therapy or antifungal prophylaxis.

The effectiveness of a person's anti-fungal immune response is severely impaired if they acquire an immunocompromised status, for example through HIV-induced AIDS, neutropenia induced by cytostatic therapy, or immunosuppressive therapy during organ transplantation. Furthermore, certain genetic variations compromise the efficacy of immune pathways and exert strong detrimental effects upon antifungal immunity. Genetic susceptibility to fungal infection has been comprehensively studied and reviewed (Lionakis 2012). Mutations in *STAT1*, for instance, predispose individuals to CMC (Puel *et al*. 2011; van de Veerdonk, Plantinga *et al*. 2011). Also, inborn errors in Th17 or CARD9 immunity are associated with recurrent mucosal and invasive candidiasis, respectively (Puel 2020). Interestingly, genetic immunodeficiencies often lead to different susceptibilities to fungal infections of the mucosal surfaces, skin, and nails. Similarly, HIV patients develop oropharyngeal candidiasis (OPC) more often than vaginal infections (VVC) (Fidel 2002). This is consistent with the existence of distinct anti-*Candida* immune mechanisms in different mucosal niches (see *Tissue-specific variability of the immune response*).

Many genetic polymorphisms in PRRs have been associated with impaired antifungal host defense (Jaeger, Stappers *et al*. 2015). For instance, SNPs in the *TLR1* and *TLR4* genes increase the risk of candidaemia (Plantinga, Johnson *et al*. 2012; Van der Graaf *et al*. 2006), and a variable number tandem repeat (VNTR) polymorphism in the *NLRP3* gene is associated with increased susceptibility to VVC (Jaeger *et al*. 2016). Susceptibility to mucosal or systemic candidiasis varies depending on the nature of the receptor or effector molecule that is mutated. For example, a homozygous mutation in the dectin-1 gene is more likely to predispose the individual to CMC (Ferwerda *et al*. 2009), whereas defects in CARD9 result in systemic, mucosal, and subcutaneous candidiasis (Drewniak *et al*. 2013; Lionakis and Holland 2013; Lanternier, Pathan *et al*. 2013). Interestingly, CARD9-deficient individuals are prone to fungal proliferation in the central nervous system (CNS), but not in the kidney, spleen, or liver (Drummond *et al*. 2015), which highlights an organ-specific CARD9-dependent immune mechanism, such as IL-1β/CXCL1-mediated neutrophil recruitment by microglial cells (Drummond *et al*. 2019).

Genome-wide association studies (GWAS) have been performed to identify genetic polymorphisms associated with susceptibility to infectious diseases (Newport and Finan 2011), and overviews of comprehensive multi-*omic* systems approaches towards an understanding of host-fungal interactions have been published (Horn *et al*. 2012; Culibrk, Croft and Tebbutt 2016). The first GWAS analysis for fungal infections identified three novel risk loci associated with increased susceptibility and severity of candidaemia: *CD58*, *LCE4A-C1orf68*, and *TAGAP* (Kumar *et al*. 2014). Although GWAS is an ideal approach for the identification of novel genetic associations with susceptibility to fungal infections, it is difficult to achieve a high level of statistical significance (<5 × 10^–8^) with the generally small cohorts of candidaemia patients available (Manolio 2010; Chapman and Hill 2012). Hence, the power of GWAS can be enhanced by combining the outputs with systems biology, transcriptomics and available knowledge of immunology and microbiology, to pinpoint disease-associated genetic determinants. The functional validation of putative hits in an independent cohort can underline the relevance of newly identified genetic associations. For instance, the integration of gene expression data and functional genomics revealed the importance of type I IFNs in the host response against *C. albicans* (Smeekens *et al*. 2013; Jaeger, van der Lee *et al*. 2015). Also, using a computational approach based on publicly available transcript profiling data sets, *MALT1, SERPINE1, ICAM1, IL8*, and *IL1A* were discovered as common immune response-inducing genes during fungal infection (Kidane, Lawrence and Murali 2013). Furthermore, combining genetic data from candidaemia cohorts with immune-profiling of *C. albicans*-stimulated cells, the *MAP3K8* and *SERPINA1* genes were shown to contribute to candidaemia susceptibility (Matzaraki *et al*. 2017). Mapping genetic determinants to variability in transcription or cytokine levels can lead to the identification of expression quantitative trait loci (eQTL) or cytokine-quantitative trait loci (cQTL), respectively (i.e. the genetic variation associated with different levels of transcriptional and cytokine responses). The analysis of eQTLs is leading to an understanding of how human genetic variation affects the anti-*Candida* host response and of the populations of cells involved in the clearance of the pathogen (de Vries *et al*. 2020). The investigation of cQTL datasets revealed *SIGLEC15* as a susceptibility factor for RVVC (M Jaeger *et al*. 2019) as well as susceptibility pathways, such as lipid homeostasis and inflammation, that affect the response of monocytes to fungal bloodstream infections (Martin Jaeger *et al*. 2019).

In addition to genetic variability, external factors such as broad-spectrum antibiotics or immunosuppression regimens negatively influence the microbial community. This, in turn, affects metabolic homeostasis (Zarrinpar *et al*. 2018) and host resistance to both antibiotic-resistant microbes and fungal pathogens (Ubeda and Pamer 2012). For instance, preexposure to antibiotics not only increases *Candida* colonisation levels in the GI tract, but also facilitates disruption of the mucosal barrier and leads to *C. albicans* bloodstream infections (Das *et al*. 2011; Gutierrez *et al*. 2020). This could be due to the loss of protection from microbiota-derived metabolites, such as short-chain fatty acids (Guinan *et al*. 2019; Gutierrez *et al*. 2020). Moreover, antibiotic-induced dysbiosis can reduce pro-inflammatory cytokine production towards LPS stimulation (Lankelma *et al*. 2016). Thus, supplementation with probiotics may represent a useful strategy to counterbalance the negative effects of antibiotic-induced therapy and improve the host immune response against fungal infections (Ubeda and Pamer 2012).

## THE MICROBIOTA

### Gastrointestinal (GI) tract

**Figure 6.**
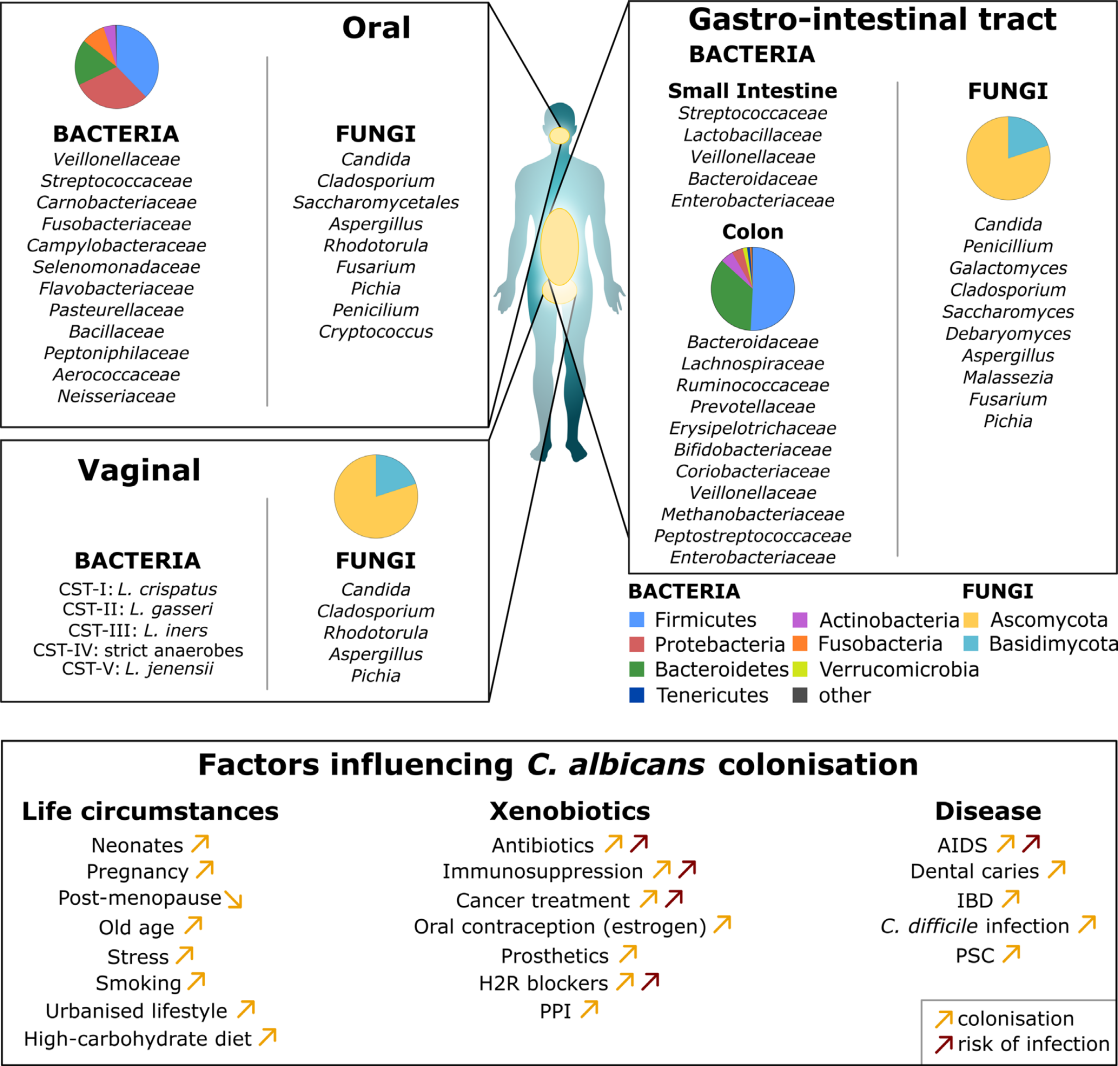
**Oral, vaginal and GI microbiota, and factors that influence *C. albicans* colonisation of these body sites**. The major microbial groups (family level for bacteria and genus level for fungi) of the healthy oral cavity (only for bacteria) (Bik *et al*. 2010; Dewhirst *et al*. 2010), GI tract (Booijink *et al*. 2010; Arumugam *et al*. 2011; Zhou *et al*. 2013; Villmones *et al*. 2018) and vagina (Human Microbiome Project Consortium 2012) are listed in decreasing order of abundance. Pie charts indicate the relative abundance of the phyla in a representative healthy oral cavity and colon (see key for colour code). The fungal component of the oral microbiota is extremely variable, and many fungi present in this compartment are likely to be transient (see text). Therefore, for the oral cavity, the fungal genera are not presented in descending order of abundance, and no pie chart is provided. The lower panel summarises factors that influence the degree of *C. albicans* colonisation (yellow) and likelihood of infection (brown arrows) of these mucosal surfaces: arrows up, increased likelihood of colonisation/infection; arrows down, decreased likelihood of colonisation/infection. See text.

The collection of microbes that colonises the GI tract is termed the ‘gut microbiota’, and is composed of bacteria, archaea, eukaryotic microbes and viruses. Thousands of microbial strains have been detected in the human gut, and these microbes can be important contributors to human health and disease (Fig. 6). For example, the gut microbiota plays key roles in nutrition (by degrading dietary components that would otherwise pass through the GI tract undigested), in host immune development and maintenance, and in protecting the host against pathogenic microbes, including *C. albicans*. This latter process, termed ‘*colonisation resistance*’, is multifactorial. It involves both microbe-microbe interactions (such as competition for nutrients, niches and binding sites, and the release of antimicrobial substances), and host-microbe interactions (whereby the microbiota can stimulate the host's immune system or strengthen the gut epithelial barrier against invading pathogens) (Lawley and Walker 2013). Significantly, an individual's degree of colonisation resistance is thought to be strongly influenced by the composition of their gut microbiota, with some individuals being more intrinsically resistant to infection than others (Ubeda *et al*. 2010).

Most gut microbiota studies have focussed on the bacterial component, which accounts for the greatest proportion of biomass present by far (Qin *et al*. 2010; Arumugam *et al*. 2011). These gut bacteria display both antagonistic and mutualistic relationships with *C. albicans* and other members of the fungal community (mycobiota) that help to maintain homeostasis in the human GI tract. Fungi represent just ∼0.1% of the GI tract biosphere, which makes the fungal mycobiota more challenging to study than the bacterial microbiota (Underhill and Iliev 2014). Also, mechanical lysis steps are best employed during DNA extraction to recover reasonable quantities of fungal DNA (Angebault *et al*. 2018), and because different extraction and sequencing methods have been used, gut mycobiota compositional analyses are difficult to compare between studies (Bellemain *et al*. 2010; Tedersoo *et al*. 2015; Tedersoo and Lindahl 2016; Huseyin *et al*. 2017; Angebault *et al*. 2018). Many mycobiota studies provide information about genera (e.g. *Candida*) rather than species (e.g. *C. albicans*). This section discusses causes of variability in the bacterial microbiota of the human GI tract and, where possible, the impact upon the GI mycobiota (fungal microbiota), and *C. albicans* in particular.

#### Variability along the GI tract

The compartments of the human GI tract, including the small intestine, caecum and large intestine (colon), have variable physiology and, as a result, each harbours distinct microbial communities (Fig. 6). Compared to the colon, the small intestine contains comparatively high levels of stomach acids, oxygen and antimicrobials, and is characterised by a short transit time. The small intestine also contains higher concentrations of bile acids, which are bactericidal to certain microbial species (Donaldson, Lee and Mazmanian 2016). Accordingly, the microbial community of the small intestine is less diverse than the colonic microbiota, and tends to be dominated by fast-growing facultative anaerobes such as streptococci and Proteobacteria that have the ability to adhere to epithelia or mucus (Zoetendal *et al*. 2012).

Moving from the small intestine, the caecum is the gateway to the colon. In the caecum, relatively long transit times and the prevailing environmental conditions favour the growth of fermentative anaerobes that can degrade complex polysaccharides, notably members of the Firmicutes and Bacteroidetes phyla (Donaldson, Lee and Mazmanian 2016). In contrast to the small intestine, the structure and physiology of the colon allows the survival of a more dense and diverse bacterial community, which can reach densities of up to 10^11^ cells per gram of colonic contents, one of the highest microbial concentrations in nature. The colon contains two layers of mucus, secreted by goblet cells, which separate colonic epithelial cells from the bacterial mass. The inner, firmly attached mucus layer is nearly sterile, whereas the outer layer is in direct contact with the luminal contents and can be a rich niche for microbial colonisation (Johansson et al. 2008, 2010). The luminal and mucosal compartments of the colon are often colonised by different profiles of bacterial populations. In a mouse model, *C. albicans* cells are visible in the lumen as well as the mucus layer (Witchley *et al*. 2019).

The fungal component of the GI tract is less diverse than its bacterial counterpart (Chehoud *et al*. 2015; Nash *et al*. 2017). Levels of fungal colonisation are lower in the small intestine, compared to the oral cavity and colon (Schulze and Sonnenborn 2009). Nevertheless, *C. albicans* can colonise the stomach, small intestine, caecum, and colon of mice (Witchley *et al*. 2019). The human intestinal mycobiota is characterised by a high inter- and intra-individual variability, which makes it difficult to define a ‘normal’ or healthy GI mycobiota composition (Nash *et al*. 2017; Raimondi *et al*. 2019). However, Ascomycota and Basidiomycota represent the two dominant phyla in the GI tract (Chehoud *et al*. 2015; Nash *et al*. 2017). The most frequently identified genera are *Candida* (e.g. *C. albicans), Saccharomyces* (e.g. *S. cerevisiae), Galactomyces, Penicillium, Aspergillus, Malasezzia* and *Debaryomyces*. Some of these fungi may not be true colonisers of the GI tract, but transient species that are brought by food or the environment. Consequently, an individual's lifestyle has a strong influence on their GI mycobiota and its variability (Auchtung *et al*. 2018; Raimondi *et al*. 2019).

#### Variability between individuals

Each individual has a distinct intestinal microbiota composition and structure. Many factors such as birth delivery mode, environmental exposure to colonising microbes, host genetics, host diet and lifestyle contribute to the unique nature of a given individual's intestinal bacterial and fungal microbiota (Qin *et al*. 2010; Salonen *et al*. 2014; Mehta *et al*. 2018), including their carriage of *C. albicans* (Neville, d Enfert and Bougnoux 2015) (Fig. 6).

#### Variability across lifespan

The mode of childbirth strongly affects the initial structure of the gut microbial community in neonates (Reyman *et al*. 2019). Bacteria such as *Enterobacter*, *Haemophilus*, *Staphylococcus* and *Veillonella* species are found in relatively high abundances in the faecal microbiota of babies born *via* caesarean (C)-section (Bäckhed *et al*. 2015). In contrast, the faecal microbiota of vaginally-delivered babies is enriched in *Bifidobacterium*, *Lactobacillus*, *Prevotella* and *Atopobium* spp., which are typically derived from the vagina of mothers (Dominguez-Bello *et al*. 2010). Babies delivered vaginally appear to be twice as likely to become colonised by *C. albicans* compared to those born by C-section (Parm *et al*. 2011).

Following birth, the main driver of gut microbiota composition is infant diet (breastfeeding *versus* formula milk). The colon of infants that are exclusively breast-fed is characterised by high numbers of milk oligosaccharide-utilising *Bifidobacterium* species (Yatsunenko *et al*. 2012; Odamaki *et al*. 2016; Hill *et al*. 2017), whereas formula-fed infants tend to possess more diverse microbiota that are less dominated by bifidobacteria (Rubaltelli *et al*. 1998; Klaassens *et al*. 2009; Lee *et al*. 2015). Following the introduction of solid food, alpha-diversity (*i.e*. the variation of microbes in a single sample) increases and the microbiota transitions towards an adult-like composition (Yatsunenko *et al*. 2012; Schei *et al*. 2017). Little is known about *C. albicans* GI primocolonisation, but this species has been detected in newborns and infants (Bliss *et al*. 2008; Schei *et al*. 2017) and in maternal milk, suggesting that breastfeeding could be a source of colonisation (Boix-Amorós *et al*. 2017).

Once established, the adult bacterial microbiota is generally considered to be quite stable over many decades (Faith *et al*. 2013), albeit allowing for temporary disruptions by factors such as antibiotic treatment or inflammation. Every adult has a distinct microbiota at the species/strain level. Nevertheless, in the mature adult gut, obligate anaerobes generally predominate, the microbiota typically containing high levels of Firmicutes and Bacteroidetes spp. (Qin *et al*. 2010). There is significant variation between studies (see *Variability due to diet and geography*), but *Candida* spp. are thought to be present in the GI tracts of over half of adults (Odds 2010; Hoffmann *et al*. 2013). However, the fungal mycobiota of the adult GI tract appears to be less stable than the bacterial microbiota (Dollive *et al*. 2013).

Further changes in the microbiota occur in the elderly (generally over 65), possibly associated with altered dietary habits and living environments, reduced metabolism and immune function, and increased antibiotic usage (Lovat 1996; Simon, Hollander and McMichael 2015). Accumulating evidence suggests that old age can be associated with a decrease in ‘beneficial’ bacteria and an increase in ‘harmful’ species (Xu, Zhu and Qiu 2019), potentially making the elderly population more susceptible to *C. albicans* colonisation (Kauffman 2001; Miranda *et al*. 2009).

#### Variability arising from host genetics, diet and geography

The composition of the gut microbiota might also be shaped to some degree by the genetics of the individual, although recent work has shown that this only has a minor impact (between 1.9% and 8.1% of gut microbiota variability) compared to other factors such as environmental exposure and host diet (Rothschild *et al*. 2018) Studies of twins have indicated that certain types of bacteria might be more influenced by host genetics than others. For example, the *Christensenellaceae* family is more likely to be influenced by host genetics, while Bacteroidetes carriage is most likely shaped by environmental factors (Simões *et al*. 2013; Goodrich *et al*. 2014).

An individual's dietary choices have major impacts on the composition of their bacterial community, and diet is therefore an important driver of inter-individual variation (Walker *et al*. 2011; David, Maurice *et al*. 2014). The main energy sources for colonic microbiota are complex plant fibres. These can be recalcitrant to degradation by host enzymes in the small intestine and therefore pass into the colon relatively intact. Additional energy sources include residual peptides and host secretions such as mucus (Derrien *et al*. 2004, 2008; Hai Li *et al*. 2015; Van den Abbeele *et al*. 2010; Lukovac *et al*. 2014; Van Herreweghen *et al*. 2017; Van den Abbeele, Gérard *et al*. 2011; Tramontano *et al*. 2018). The type and availability of these various nutrients exert selective effects on numerous groups of bacteria. The starkest difference is between animal-based (high fat and protein content) and plant-based diets (rich in plant polysaccharides). Plant-based diets lead to increases in *Prevotella* species and Firmicutes, whereas animal-based/lower fibre diets stimulate an increase in *Bacteroides* and bile-tolerant species such as *Bilophilia* and *Alistipes* (Wu *et al*. 2011; David, Maurice *et al*. 2014; De Filippis *et al*. 2016; Pareek *et al*. 2019).

Differences in dietary habits between people inhabiting different regions of the world (He *et al*. 2018), and between those living in urbanised versus rural settings, are considered to be a main driver of geographical variation in microbiota composition. Indeed, the migration of people from rural to westernised settings greatly impacts the composition of their resident intestinal microbiota (Vangay *et al*. 2018). Those living in less urbanised societies typically consume greater amounts of dietary fibre and less meat and processed foods. Consequently, they tend to have a greater predominance and prevalence of more specialist fibre-degrading bacteria in their gut. In contrast, people living in cities or more urbanised countries, are characterised by their consumption of more refined, high protein and high fat diets, and they harbour microbial communities with reduced diversity (Schnorr *et al*. 2014).

Geographic location also affects the prevailing mycobiota, especially as many of the fungi detected in the GI tract are not true colonisers, but only transient species brought in as a result of different diets/environmental exposures (Auchtung *et al*. 2018; Raimondi *et al*. 2019). For example, *Aspergillus oryzae*, a species used to ferment soybeans to make soy sauce, is often present in the guts of healthy Japanese (Motooka *et al*. 2017). Also, the relatively high abundance of *Penicillium* and *Debaryomyces* spp. in Sardinian volunteers has been linked with high levels of cheese consumption in this region (Wu *et al*. 2020). Interestingly, the GI tracts of Wayampi Amerindians harbour a relatively high abundance of *Candida krusei* and *S. cerevisiae*, and less *C. albicans*, compared to individuals with more industrialised lifestyles (Angebault *et al*. 2013). However, the carriage of *Candida* spp. in the gut has been negatively associated with amino acid-, protein-, and fatty acid-rich diets (Hoffmann *et al*. 2013), which are characteristic of urbanised societies, suggesting that geographically-related factors other than diet may also affect the likelihood of *Candida* carriage in the gut. These may include exposure to environmental stressors and pollutants, including antibiotics (Jin *et al*. 2017; Karl *et al*. 2018) (see below).

#### Variability due to lifestyle and xenobiotics

A range of non-dietary lifestyle factors can also impact the gut microbiota and its resilience against invading pathogens. For example, exposure to stress is thought to lower the numbers of potentially beneficial gut bacteria such as *Lactobacillus* spp., and this has been postulated to have multiple effects on colonic motor activity *via* the gut-brain axis (Grenham *et al*. 2011; Galley *et al*. 2014; Murakami *et al*. 2017). Therefore, *Lactobacillus* spp. have been proposed as candidates for probiotic intervention (Bravo *et al*. 2011). Interestingly, preliminary studies have shown that *Lactobacillus* might reduce *C. albicans* overgrowth (Drutz 1992; Ceresa *et al*. 2015; Morais *et al*. 2017), and reductions in the prevalence of *Lactobacillus* spp. in the gut may be associated with stress-induced candidiasis (Meyer, Goettlicher and Mendling 2006; Akimoto-Gunther *et al*. 2016). Indeed, *L. rhamnosus* has been shown to reduce the capity of *C. albicans* to damage epithelial barriers and translocate into the ‘bloodstream’ in an intestine-on-chip model (Graf *et al*. 2019; Maurer *et al*. 2019).

Many xenobiotics interact with, and influence, the gut microbiota. In turn, these may increase the risk of developing opportunistic infections by disrupting colonisation resistance. Antibiotics have been the most studied xenobiotics. In addition to treating the aetiological agent of a disease, long-term broad-spectrum antibiotics can exert collateral damage upon beneficial indigenous gut bacteria (Dethlefsen *et al*. 2008; Fouhy *et al*. 2012; Burdet *et al*. 2019). This can have the unintended effect of suppressing colonisation resistance, leading to the outgrowth of opportunistic pathogens. This includes *C. albicans*, as antibiotic treatments permit persistent *C. albicans* colonisation of the GI tract in mice that are normally resistant to colonisation (Fan *et al*. 2015). Several studies have attempted to define the mechanisms underlying this outgrowth (Guinan *et al*. 2019; Gutierrez *et al*. 2020; Zhai *et al*. 2020). Cefoperazone-treated mice display reduced levels of the short-chain fatty acids generated by the gut microbiota, which enhances *C. albicans* growth, morphogenesis and biofilm formation (Guinan *et al*. 2019). On the other hand, the outgrowth of *C. albicans* in antibiotic-treated mice has been linked to increased levels of carbohydrates, sugar alcohols and primary bile acids as well as decreases in carboxylic acids and secondary bile acids (Gutierrez *et al*. 2020). Although the effect of antibiotics on the gut mycobiota of healthy humans remains largely unknown, the administration of antibiotics to immunocompromised patients has been associated with decreases in the diversity of the gut microbiota and marked expansions in the burdens of pathogenic *Candida* species (Zhai *et al*. 2020). The extent of overall microbiota recovery after cessation of antibiotic treatment depends on the spectrum of activity of the antibiotic, the length of time it was administered, and the underlying composition of the baseline gut microbiota. In general, the microbiota appears to be reasonably resilient to short courses of certain antibiotics, displaying an ability to recover after treatment with, for example, ciprofloxacin (Pop *et al*. 2016) or azithromycin (Wei *et al*. 2018). However, recovery is not always complete (Dethlefsen *et al*. 2008; Fouhy *et al*. 2012).

Attention has also turned towards the susceptibility of the microbiota to non-antibiotic xenobiotics, many of which are commonly used drugs (Jackson *et al*. 2018; Maier *et al*. 2018; Vich Vila *et al*. 2020). Proton pump inhibitors (PPIs) have been the most studied non-antibiotic xenobiotics (Jackson *et al*. 2018; Vich Vila *et al*. 2020). Some evidence suggests that the use of PPIs increases the risk of *Candida* colonisation in intensive care patients (Mojazi Amiri *et al*. 2012; Jacobs *et al*. 2015). Histamine-2 receptor blockers also disturb colonisation resistance against opportunistic infections, primarily *C. albicans* (Saiman *et al*. 2001).

#### Variability associated with illness

Perturbations in the GI tract microbiota are associated with a multitude of disorders such as Inflammatory Bowel Disease (IBD), diabetes, obesity, colorectal cancer and cirrhosis. IBD includes conditions such as Crohn's disease (CD) and ulcerative colitis (UC). These diseases can further drive variability within the gut microbiota. Increasing evidence suggests that people suffering from some of these conditions display even more inter-individual variability than healthy controls (Zaneveld, McMinds and Vega Thurber 2017). IBD patients tend to have reduced overall microbiota diversity with decreased prevalence of potentially beneficial Firmicutes lineages such as *Faecalibacterium prausnitzii*. They also have increased levels of opportunistic pathogens such as *Enterobacteriaceae*, which are better able to thrive in an inflammatory environment than many other obligately anaerobic gut commensals (Manichanh *et al*. 2006; Sokol *et al*. 2009; Pascal *et al*. 2017; Franzosa *et al*. 2019; Lloyd-Price *et al*. 2019). IBD patients often show a disequilibrium in the diversity of bacteria and fungi in their GI tracts (Wheeler *et al*. 2016), which suggested that *Candida* spp. might also play a role in IBD pathogenesis. *C. albicans*, and the *Candida* genus in general, are more abundant in IBD patients (Ott *et al*. 2008; Kumamoto 2011; Chehoud *et al*. 2015; Sokol *et al*. 2017). Recent data indicate that *Malassezia*, rather than *Candida*, is associated which Crohn's disease (Limon *et al*. 2019). Nevertheless, a positive clinical response to faecal microbiota transplantation in ulcerative colitis patients has been associated with high levels of *Candida* spp. colonisation before treatment and decreased *Candida* abundance in the gut following treatment (Leonardi *et al*. 2020).

Patients with primary sclerosing cholangitis (PSC) also harbour decreased bacterial diversity, while the fungal diversity in their GI tract is increased (Lemoinne *et al*. 2020). Patients with *Clostridioides difficile* infections that have received a faecal microbiota transplant, often show reduced fungal diversity and *C. albicans* outgrowth in their gut. Indeed, a high abundance of *C. albicans* in the donor's gut might compromise the success of the faecal transplantation (Zuo *et al*. 2018). Alcoholic hepatitis has been associated with an increase in the abundance of *Candida* spp. in the gut mycobiota and a decrease of fungal diversity (Lang *et al*. 2020), while an outgrowth of *Candida* spp. has also been observed in children suffering from autistic spectrum disorders (Strati *et al*. 2017). Therefore, changes in the gut mycobiota are associated with, and potentially contribute to, a wide range of pathologies.

### Oral cavity

Defining the core oral microbiota for a healthy individual is complicated by the fact that the oral cavity is a primary entry point for microbes in food and from the environment. Thus, microbes identified in the oral cavity may be transient, and washed out through saliva before having any impact upon health, rather than being active colonisers of this niche. Nevertheless, the oral cavity does harbour the second largest microbiota, in terms of diversity, compared to other body sites (Zhou *et al*. 2013) (Fig. 6).

#### Variability between individuals

Many of the factors that contribute to the variability of GI tract microbiota have a similar impact upon the microbiota present at other body sites, such as the oral environment (Fig. 6).

#### Variability across lifespan

The development of the oral microbiota in infants is influenced by their mode of delivery (Lif Holgerson *et al*. 2011; Dzidic *et al*. 2018). Infants born by C-section initially have more oral colonisers, such as *Staphylococcus, Corynebacterium* and *Propionibacterium* spp., which are derived from human skin (Dominguez-Bello *et al*. 2010). In contrast, babies born vaginally have bacterial communities reflecting their mothers’ vaginal bacterial communities, dominated by *Lactobacillus, Prevotella* and *Sneathia* spp. *Candida* spp. are identified more frequently in the oral microbiota of newborns that were vaginally born, especially by mothers whose vagina was colonised by *Candida* (Al-Rusan, Darwazeh and Lataifeh 2017).

After 6 months of age, the impact of delivery mode is gradually eliminated as microbial patterns converge to that observed for older individuals. The oral microbial communities then evolve together over time with the host. This applies to both the oral bacterial and fungal microbiota. However, no consistent pattern has emerged for fungal colonisation, with conflicting results observed between studies (Baley *et al*. 1986; Caramalac *et al*. 2007; Farmaki *et al*. 2007; Bliss *et al*. 2008; Siavoshi *et al*. 2013; Filippidi *et al*. 2014; Stecksén-Blicks *et al*. 2015; Ward *et al*. 2018). Some studies have suggested vertical transmission from mother to child (Caramalac *et al*. 2007; Filippidi *et al*. 2014). Other studies consider breastmilk to be a source of fungal colonisation, with *Malassezia* (44%), *Candida* (19%) and *Saccharomyces* (12%) being the main taxa detected within one month of birth (Boix-Amorós *et al*. 2017). However, once again, no consistent pattern has emerged (Darwazeh and al-Bashir 1995; Matee *et al*. 1996; Mattos-Graner *et al*. 2001; Kadir, Uygun and Akyüz 2005; Neves *et al*. 2015; Stecksén-Blicks *et al*. 2015). The development of the oral mycobiota can also be influenced by nail biting and finger sucking, which might enhance the colonisation by microbes usually found on the skin (e.g. *Malassezia* spp.) (Dupuy *et al*. 2014), and the use of pacifiers has also been correlated with increased fungal colonisation (Darwazeh and al-Bashir 1995; Mattos-Graner *et al*. 2001; Zöllner and Jorge 2003).

By the age of three, children have developed a complex oral microbial community, although they carry higher levels than older children of *Pseudomonadaceae, Moraxellaceae* and *Enterobacteriaceae*, which are not usually associated with healthy commensal oral microbiota (Crielaard *et al*. 2011). The oral bacterial microbiota of healthy adults is marked by increased proportions of Bacteroidetes (*Prevotella* spp.), Spirochaetes, Actinobacteria and Firmicutes (Keijser *et al*. 2008; Crielaard *et al*. 2011). The fungal taxa most frequently isolated from the oral cavity are *Candida* spp. and *S. cerevisiae* (foodborne) (Baley *et al*. 1986; Darwazeh and al-Bashir 1995; Matee *et al*. 1996; Mattos-Graner *et al*. 2001; Zöllner and Jorge 2003; Kadir, Uygun and Akyüz 2005; Farmaki *et al*. 2007; Filippidi *et al*. 2014; Neves *et al*. 2015; Ward *et al*. 2018). *C. albicans* is the *Candida* species most frequently isolated from the oral cavity, although other species such as *C. tropicalis*, *C. krusei*, *C. kefyr* and *C. glabrata* have also been detected.

After maturation of the microbiota, the oral cavity is thought to have the most stable microbial profile among all body sites (Zhou *et al*. 2013). Several studies have analysed temporal variation in the salivary microbiota (Caporaso *et al*. 2011; David, Materna *et al*. 2014; Flores *et al*. 2014; Belstrøm *et al*. 2016). This revealed high variability in the relative abundances of taxa, with, for instance, greater stability in individuals harbouring a more diverse tongue community (Flores *et al*. 2014). As with the GI tract microbiota, there is evidence that the oral microbiota can be influenced by birthplace and current geographic residence (Xu and Mitchell 2003; Wang *et al*. 2013).

Supragingival, tongue and salivary communities display strong inter-individual and inter-site differences (Hall *et al*. 2017). Among all the anatomical sites of the oral cavity, the supragingival plaque community is distinct from that of the tongue plaque and the saliva, with high similarity between the tongue and saliva. The supragingival plaque harbours a bacterial community with much lower diversity compared with that of the tongue and the salivary communities. Saliva has the highest number of bacterial taxa while supragingival plaque has the lowest. Hall and co-workers (Hall *et al*. 2017) identified 26 core taxa, belonging to five phyla (Actinobacteria, Bacteroidetes, Firmicutes, Fusobacterium, and Proteobacteria), across all sites. Few taxa were shared among all sites.

#### Variability arising from diet

Dietary factors contribute to the variation of oral microbial communities. In general, foods are swallowed quickly after a short period of mastication. Nevertheless, the introduction or sudden lack of certain nutrients can cause shifts in the oral microbiota (Adler *et al*. 2013; Zheng *et al*. 2015). For example, microbes that contribute to folate biosynthesis, such as *Streptococcus*, increase after long-term deprivation of fresh fruit and vegetables, which are rich in folic acid (Zheng *et al*. 2015). Vegetarians and non-vegetarians display similar rates of *C. albicans* carriage in their oral microflora (Patil *et al*. 2017). Fungi are introduced to the oral cavity *via* food and drink. Therefore, fungi commonly derived from fermented beverages such as beer are often isolated from the oral cavity (Fan *et al*. 2018).

#### Variability associated with illness

The most common oral conditions include tooth decay, periodontal disease and oral cancer. While many intestinal diseases have been associated with gut dysbiosis, there is still debate as to whether oral diseases are correlated with oral microbial diversity. For example, periodontal disease patients display more diverse and complex oral microbial communities than peri-implantitis patients (Kumar *et al*. 2012; Liu *et al*. 2012). Nevertheless, pathogenic *Streptococcus* spp. promote caries by lowering oral pH, which results in the demineralisation of enamel (Ajdić *et al*. 2002; Mei *et al*. 2013; Ito *et al*. 2019). Interactions between bacteria and fungi are likely to be of relevance to oral health. Interestingly, a study assessing *Candida* load and the bacterial composition of saliva in a Dutch cohort revealed that a low diversity of salivary microbiota characterised by dominant acidogenic bacilli (streptococci and lactobacilli) is positively correlated with elevated *Candida* burdens and possible overgrowth (Kraneveld *et al*. 2012). However, only certain diseases correlate with fungal colonisation of the oral cavity. These include, but are not limited to, HIV/AIDS (Cassone and Cauda 2012), cancer treatments (Silk 2014), dental caries (Falsetta *et al*. 2014) and oral lesions (ulcerations, nodules or granulomas) (Muzyka and Epifanio 2013). All of these conditions are linked either to the creation of novel niches that are not naturally present, or to perturbation of immune function. They are often correlated with *Candida* overgrowth (*C. albicans* in 70–80% cases), leading to oropharangeal candidiasis, particularly in immunocompromised individuals (Millsop and Fazel 2016).

### Vaginal mucosa

#### Variability between individuals

Interactions between the resident microbiota and *C. albicans* in the vaginal tract are important for pathogenesis (Fig. 6). The vaginal bacterial microbiota of healthy reproductive-age women is generally dominated by *Lactobacillus* spp. (Ravel *et al*. 2011). Lactic acid production by these bacteria contributes to a healthy vaginal pH that is commonly lower than 4.5 (Ravel *et al*. 2011). The vaginal bacterial microbiota can be further sub-classified into five main community state types (CSTs): CST-I (*Lactobacillus crispatus*-dominated); CST-II (*L. gasseri*-dominated); CST-III (*L. iners*-dominated); and CST-V (*L. jensenii*-dominated) (Ravel *et al*. 2011). The CST-IV state is extremely diverse compared to the other types, comprising anaerobes and species linked to bacterial vaginosis (BV). CST-IV has been further divided into subgroups: CST-IVA (containing some lactobacilli); CST-IVB (high prevalence of *Atopobium* spp.); CST-IVC (*Gardnerella* subgroup A-dominated); and CST-IVD (*Gardnerella* subgroup C-associated) (Gajer *et al*. 2012; Albert *et al*. 2015). Women can transition between these CST states, for example, during menses (Gajer *et al*. 2012). CST-I has been associated with *C. albicans* colonisation (Sarah E Brown *et al*. 2019), but more studies are required to fully understand the complexity of the vaginal microbiota and its potential association with disease.

Less is known about the mycobiota of the human vagina. Culture-dependent studies indicate that *C. albicans* is the most abundant fungal species, although its abundance has been shown to vary according to lifestyle, age, ethnicity, hygiene habits and contraceptive methods (Fischer 2010; Wei, Feng and Luo 2010; Fischer and Bradford 2011; Shaaban *et al*. 2015; Donders *et al*. 2017, 2018). Indeed, intrauterine contraceptive systems have been reported to be associated with a rise in *C. albicans* colonisation, while progesterone-only pills result in lower rates of colonisation (Donders *et al*. 2017, 2018). However, due to the relatively low sensitivity of conventional culture approaches, these studies may underestimate the true fungal diversity of the vagina (Guo *et al*. 2012; Drell *et al*. 2013). Using 18S rRNA gene sequencing, Guo and co-workers demonstrated that the healthy vaginal mycobiota was mainly composed of *Ascomycota* (∼70% relative abundance), with the *Candida* genus dominating, and *C. albicans* as the main species. *Basidiomycota* were also detected, but with a lower proportional abundance (Guo *et al*. 2012). These results were confirmed using ITS1 pyrosequencing (Drell *et al*. 2013). Taking these findings together, the most abundant fungi in the healthy vaginal tract appear to be *C. albicans, S. cerevisiae* and *C. tropicalis* (Guo *et al*. 2012). Recent data indicate an association between the type of *Lactobacillus* species present and the likelihood of *Candida* colonisation (Tortelli *et al*. 2020).

#### Variability associated with age and pregnancy

The vaginal bacterial microbiota is influenced by oestrogen levels and is most stable when these are high (Gajer *et al*. 2012) (Fig. 6). Prepuberty is characterised by a bacterial microbiota comprised of anaerobes, diphtheroids, lactobacilli, streptococci, *Staphylococcus epidermidis*, and *Escherichia coli* (Hammerschlag *et al*. 1978). During puberty, increased oestrogen stimulates thickening of the glycogen-rich vaginal epithelium and establishes a vaginal microbiota dominated by lactobacilli (Miller *et al*. 2016). High oestrogen levels in reproductive women create unique features for the vaginal mucosa (Kalia, Singh and Kaur 2020), inducing a tolerogenic immune repertoire through immunomodulation of the neutrophil response (Willems *et al*. 2020). When glycogen is degraded by host α-amylases, products such as maltose and maltotriose foster the growth of *Lactobacillus*, leading to a reduced vaginal pH (Spear *et al*. 2014).

*Lactobacillus* spp. typically dominate the vaginal microbiota during pregnancy, and increased levels of these bacteria were reported in *Lactobacillus*-dominated CSTs compared to non-pregnant women (Aagaard *et al*. 2012; Romero *et al*. 2014; MacIntyre *et al*. 2015; Freitas *et al*. 2017). During pregnancy, the microbiota is also characterised by a lower occurrence of *Mollicutes*, and by members of the orders Clostridiales, Bacteroidales, and Actinomycetales (Aagaard *et al*. 2012; Freitas *et al*. 2017). Sampling six weeks postpartum revealed that bacterial diversity increases following birth and the vaginal microbiota readily assumes CST-IV (MacIntyre *et al*. 2015).

Fluctuations in oestrogen levels probably underlie variations in the abundance of *C. albicans*. Indeed, oestrogen injection is required to promote *C. albicans* colonisation of the vagina in rats and mice (Cheng, Yeater and Hoyer 2006). This is probably linked to the stimulatory effects of oestrogen upon *C. albicans* morphogenesis (White and Larsen 1997; Tarry *et al*. 2005). In humans, rising oestrogen levels during pregnancy has also been associated with an increase in *C. albicans* colonisation (Goplerud, Ohm and Galask 1976), which can potentially lead to premature birth (Roberts *et al*. 2011).

Postmenopausal women are also more likely to display a CST-IV microbiota (Brotman, Shardell *et al*. 2014). Menopausal women frequently experience a loss of lactobacilli and an increased vaginal pH (Brotman, Shardell *et al*. 2014; Gliniewicz *et al*. 2019). This elevation in vaginal pH, combined with an increase in vaginal glycogen levels, may contribute to the reduced incidence of VVC observed after the menopause (Hillier and Lau 1997; Spinillo *et al*. 1997). The reduced levels of oestrogen may also explain the decreased rates of VVC in postmenopausal women (Nwokolo and Boag 2000). Consequently, hormone replacement therapy (HRT) is a risk factor for VVC in these women (Fischer and Bradford 2011). HRT can restore a *Lactobacillus* dominated microbiota, similar to that of premenopausal women (Gliniewicz *et al*. 2019), and this treatment increases the likelihood of postmenopausal women succumbing to VVC (Fischer and Bradford 2011). However, more comprehensive studies are needed for a better understanding of the relationship between the overall vaginal mycobiota and health and disease.

#### Variability relating to geography and ethnicity

Independent of geography, the vaginal microbiota of women is dominated by *Lactobacillus* (Anukam *et al*. 2006; Shi *et al*. 2009; Zhou *et al*. 2010; Ravel *et al*. 2011; Pendharkar *et al*. 2013; Albert *et al*. 2015; Madhivanan *et al*. 2015). Nevertheless, the dominating species of the *Lactobacillus* genus may differ between geographical regions. Similar rates of vaginal colonisation by *Candida* spp. (11–17%) have been reported for asymptomatic women from European, South American and Middle Eastern countries (Gonçalves *et al*. 2016). However, VVC rates differ significantly for symptomatic women around the world, ranging from 12% to 57%, and most cases are caused by *C. albicans* (Gonçalves *et al*. 2016).

Regarding ethnic differences, Asian and Caucasian women from North America are mainly colonised by *Lactobacillus* spp. (CST-I, II, III and V*)*, whereas black and Hispanic women are more likely to be colonised by CST-IV communities (Ravel *et al*. 2011). Women of African, American and European ancestry are more likely to be colonised by *L. iners* and *L. crispatus*, respectively (Fettweis *et al*. 2014). However, the basis for these differences is not clear (Gupta, Kakkar and Bhushan 2019). The evidence for different VVC rates between ethnic groups is limited (Wei, Feng and Luo 2010). Further studies would be required to define whether significant differences exist, and to parse apart the basis for any apparent differences.

#### Variability arising from lifestyle and xenobiotics

A number of factors influence the vaginal microbiota and, consequently, may predispose women to infection or aid in preventing infection (Fig. 6). The effects of antibiotics on the microbiota of women with vaginal infections are well studied. Metronidazole treatment of women with BV has been shown to increase prevalence of *Lactobacillus* spp. (Mayer *et al*. 2015). Similarly, the vaginal microbiota becomes dominated by *L. iners* when azithromycin is administered to treat *Chlamydia trachomatis* (Tamarelle *et al*. 2020). The composition of the lactobacilli community can shift in response to antibiotics, since vaginal *Lactobacillus* spp. have varying antibiotic sensitivity profiles (Melkumyan *et al*. 2015). Pregnant women frequently become colonised with *Staphylococcus* when receiving antibiotic treatment (Stokholm *et al*. 2014). It is well known that antibiotic treatments predispose individuals to VVC if they are already colonised with *Candida* spp. (Sobel 2007).

Not much is known regarding the impact of diet on the vaginal bacterial microbiota. Individuals consuming fibre-rich diets are less likely to have BV-associated microbiota (Shivakoti *et al*. 2020). Ingestion of micronutrients such as the zwitterion betaine (an osmolyte and methyl donor) may result in a microbiota that is predominantly lactobacilli (Tuddenham *et al*. 2019). In addition, smoking reduces vaginal lactobacilli (potentially *via* amines, anti-oestrogenic effects and bacteriophage induction) and increases the probability of acquiring a CST-IV microbiota (Brotman, He *et al*. 2014). As the vaginal microbiota influences the vaginal mycobiota (Sobel 2007), these effects are likely to influence *C. albicans* colonisation.

### Translational opportunities

Given the impact that the microbiota appears to have on susceptibility to *Candida* infections, there are clear potential therapeutic benefits to bolstering our microbial communities at various body sites. Probiotics provide a means of altering the microbiota. Probiotics are defined as ‘*live microorganisms that, when administered in adequate amounts, confer a health benefit on the host*’ (Hill *et al*. 2014). Currently, robust evidence for clinical efficacy is limited to a relatively narrow set of conditions. However, there is clear potential to widen this applicability to IBD, for example (Rondanelli *et al*. 2017). Interactions between the microbiota and host are thought to play key roles in *Candida* colonisation and pathogenesis, and therefore, live biotherapeutic products (LBPs) that exert anti-*Candida* effects are worthy of further study. (LBPs are products containing live microbes that are used to prevent or treat a medical condition.) Some are under development (Poupet *et al*. 2019). These have the potential to dramatically ameliorate the economic and health burden imposed by this fungus and reduce the risk of vulnerable individuals to *Candida* infections. Several types of patient cohort may benefit from such an approach.

Premature neonates are among those most at risk of developing systemic *Candida* infections. Compared to full-term healthy babies, premature newborns have an altered microbiota and can be colonised by opportunistic pathogens (Hill *et al*. 2017; Korpela *et al*. 2018). In addition to weakened colonisation resistance, their immature immune system places them at risk of late-onset sepsis caused by *C. albicans*, which has colonised their GI tract through vertical transmission from their mothers, or from the hospital environment (Bliss *et al*. 2008). Supplementation with a *Lactobacillus* probiotic (*L. rhamnosus* and *L. reuteri*) results in lower GI tract colonisation of *Candida* spp. compared to controls (Manzoni *et al*. 2006; Romeo *et al*. 2011). *L. reuteri* was found to be as effective as the antifungal nystatin in preventing candidaemia (Oncel *et al*. 2015). Replacing prophylactic antifungal treatments with LBP-based therapy would have the advantage of reducing the selection for antifungal drug resistance. Additionally, LBPs may benefit premature neonates by preventing GI symptoms such as regurgitation, vomiting and abdominal distension (Indrio *et al*. 2008; Rougé *et al*. 2009; Romeo *et al*. 2011), while reducing hospitalisation time (Romeo *et al*. 2011). Consequently, the use of LBPs for premature neonates may help decrease their risk of developing nosocomial infections.

HIV positive individuals are another group at high risk of developing *C. albicans* infections, especially oropharyngeal candidiasis (Patil *et al*. 2018). Administration of probiotic strains with anti-*Candida* activity could prevent the development of such infections by reducing levels of fungal colonisation (Hu *et al*. 2013).

Studies have shown that LBPs are more successful than placebos at preventing recurrence of vulvovaginal candidiasis (Vladareanu *et al*. 2018). Furthermore, therapies that combine LBPs with azoles have been shown to improve drug efficacy by restoring the local bacterial community (Kovachev and Vatcheva-Dobrevska 2015; Russo *et al*. 2019). Increasing the efficacy of the antifungal drug in this way may allow drug doses to be reduced.

Taken together, current evidence is encouraging and suggests that probiotics can be used to prevent *C. albicans* infections in vulnerable cohorts. However, further studies are required to identify optimal LBP candidates, and to understand the underlying mechanisms of action that result in clinical efficacy.

## THE *FUNGUS-HOST-MICROBIOTA* INTERPLAY

The previous sections describe the multifaceted nature of *C. albicans* interactions with the host, how antifungal immunity influences these interactions, and how the microbiota is closely related to host physiology and impacts *C. albicans* colonisation. These tripartite interactions between host, fungus and microbiota are incredibly complex and strongly influence the likelihood and outcomes of *Candida* infections. Here we discuss the nature of interdependencies within the fungus-host-microbiota interplay and their impact on health and *Candida* infections, in particular.

### Synergistic and antagonistic interactions between kingdoms

**Figure 7.**
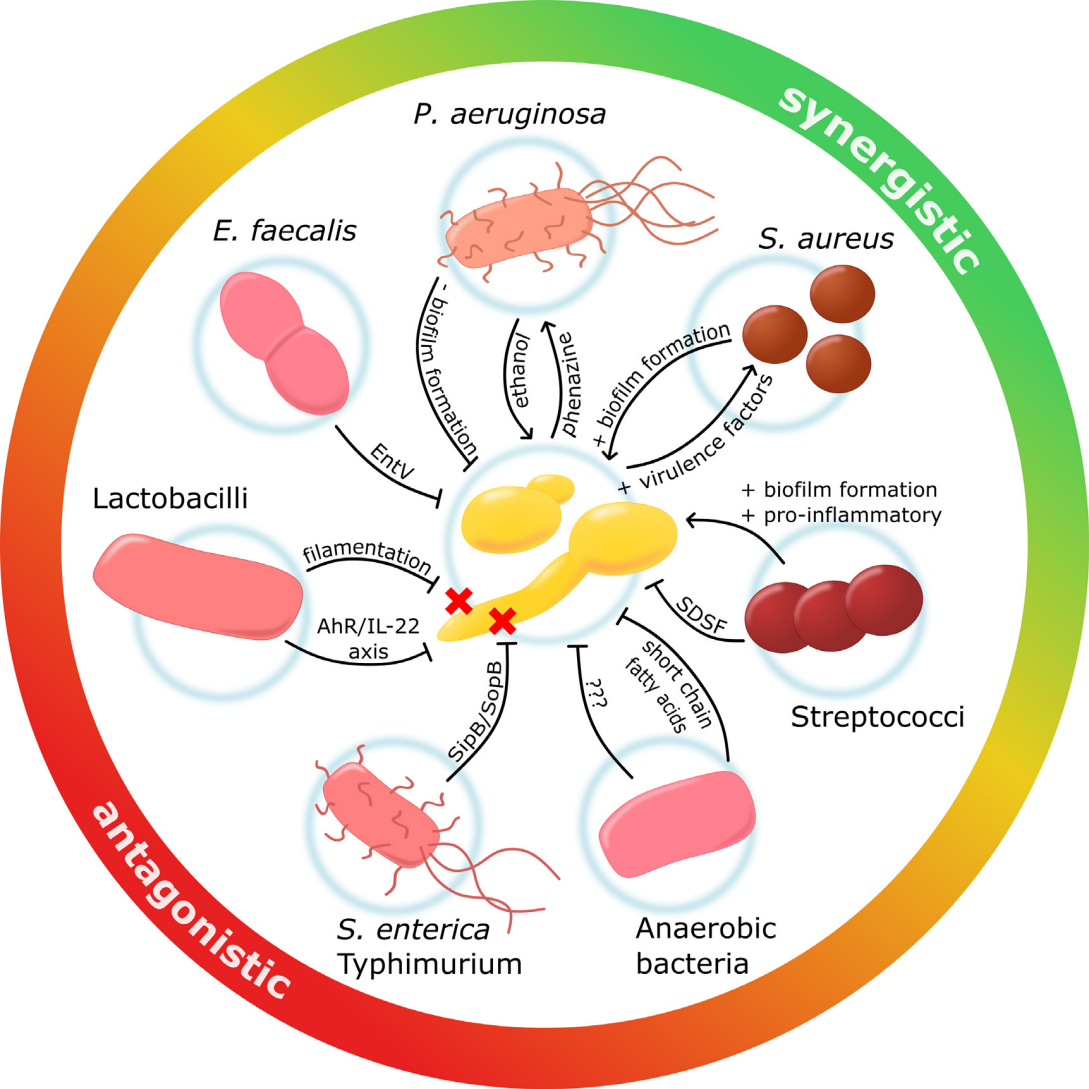
**The interplay between *C. albicans* and certain bacterial present in human microbiotas**. The growth of *C. albicans* in mucosal niches is generally constrained by the local bacterial microbiota *via* colonisation resistance. However, specific interactions with certain bacteria present in the vagina, oral cavity and/or GI tract influence the growth and/or virulence of *C. albicans* more directly. These interactions can be antagonistic (i.e. reduce the growth and virulence of the fungus) or synergistic (i.e. enhance the growth or virulence of the fungus). Anaerobic bacteria antagonise *C. albicans* colonisation by mechanisms that include the production of short chain fatty acids. *S. enterica* Typhimurium kills *C. albicans* hyphae by injecting effectors into the fungus via the SopB translocase. Lactobacilli antagonise *C. albicans* colonisation acidifying the local environment which reduces filamentation, and by generating metabolites that enhance IL-22-mediated immunity. *E. faecalis* blocks yeast-hypha morphogenesis and biofilm formation using *EntV*. Interactions between *C. albicans* and *P. aeruginosa* mutually enhance their virulence *via* cross-talk involving ethanol production by the fungus, which promotes toxic phenazine production by the bacterium, and this in turn promotes alcohol production by *C. albicans*. *S. aureus* binds *C. albicans* hyphae and promotes biofilm formation and antimicrobial resistance. Furthermore, co-infection with *S. aureus* significantly enhances the lethality of *C. albicans*. Streptococci block the formation of *C. albicans* hyphae via the diffusible factor, SDSF, but potentially co-exist as commensals with *C. albicans*. See text.

Many researchers have focused on antagonistic interactions between *C. albicans* and bacteria species because these could potentially be exploited in therapeutic approaches (see *Impact of a changing microbiota on the Fungus-Host-Microbiota interplay* and *Translational opportunities*). However, it is estimated that approximately 30% of all *Candida* bloodstream infections are polymicrobial and involve both fungi and bacteria (Klotz *et al*. 2007). This suggests that synergism can occur between *C. albicans* and certain bacterial species (Fig. 7). [In this context, ‘synergism’ describes polymicrobial interactions during which one microorganism promotes colonisation or infection by another (Brogden, Guthmiller and Taylor 2005)].

*Candida albicans* interacts with different types of resident microorganisms depending on the body site (see *The Microbiota*). *Candida albicans* synergises with various streptococcal species that are abundant in the oral cavity, through physical interactions that enhance bacterial growth and adhesion, lead to more pronounced biofilm formation and, in some cases, increase fungal invasion (Silverman *et al*. 2010; Diaz *et al*. 2012; Metwalli *et al*. 2013; Xu *et al*. 2016; Koo, Andes and Krysan 2018; Vila *et al*. 2020). Molecules involved in these physical interactions include bacterial adhesins (Holmes, McNab and Jenkinson 1996; Silverman *et al*. 2010), specific fungal surface proteins (Holmes, McNab and Jenkinson 1996; Dutton *et al*. 2014; Xu *et al*. 2017), and components of the extracellular polysaccharide matrix produced in biofilms (Falsetta *et al*. 2014). Communication within mixed biofilms involves bacterial and fungal quorum sensing systems that influence the expression of virulence factors and bacterial metabolism (Sztajer *et al*. 2014; He *et al*. 2017; Kim *et al*. 2017). In addition to this direct synergism, streptococcal co-infection stimulates complex immune reactions that promote the expression of proinflammatory cytokines and enhanced tissue inflammation in a murine model of *C. albicans* thrush (Xu *et al*. 2014). The clinical importance of these synergistic interactions is suggested by co-colonisation of bacteria with fungi in oral diseases such as childhood caries, periodontitis, and denture stomatitis (O'Donnell *et al*. 2015). Furthermore, a causal relationship between bacterial-fungal co-infection and disease severity has been demonstrated for caries in a rat model (Falsetta *et al*. 2014). Fungal colonisation also affects oral microbiota composition, which encourages invasive infection (Bertolini *et al*. 2019).

The synergistic cross-kingdom interactions between *C. albicans* and *Staphylococcus aureus* have been comparatively well studied (Carolus, Van Dyck and Van Dijck 2019) (Fig. 7). *Staphylococcus aureus* can bind to *C. albicans* hyphae, which indirectly enhances the attachment of the bacterium to abiotic surfaces and promotes the formation of mixed biofilms with increased resistance to antimicrobial compounds (Shirtliff, Peters and Jabra-Rizk 2009; Harriott and Noverr 2010; Peters *et al*. 2010; Harriott and Noverr 2011; Kong *et al*. 2016; Kean *et al*. 2017). Even more striking, though, is the enhanced lethality observed following co-infection in a mouse model (Peters and Noverr 2013). A synergistic enhancement of virulence occurs, independent of the ability of *C. albicans* to form filaments (Nash *et al*. 2016). This synergy is driven by an augmented host immune response (Nash *et al*. 2016, 2014; Peters and Noverr 2013). In addition, the presence of *C. albicans* increases the expression of staphylococcal virulence factors by modifying the environment (Todd *et al*. 2019; Todd, Noverr and Peters 2019). Significantly, this synergistic virulence depends on the *Candida* species involved, and was not observed for the closely related, but less virulent species, *Candida dubliniensis*.

Some relationships are difficult to place within a dichotomous scheme of synergism or antagonism. In some cases, diffusible molecules underlie inter-kingdom interactions (Deveau *et al*. 2018) as well as microbiota-induced immunomodulation of the host (Blacher *et al*. 2017). Most bacterial molecules target *C. albicans* virulence factors. *Salmonella enterica* serovar Typhimurium uses SipB translocase to inject SopB effectors and induce killing of the fungal hypha (Kim and Mylonakis 2011). *Enterococcus faecalis* restricts biofilm development by preventing yeast-to-hypha transition *via* the bacteriocin inhibitor *EntV* (Graham *et al*. 2017). *Streptococcus mutans* prevents hypha formation by targeting *HWP1* (a hyphal-specific gene) using Streptococcus Diffusible Signal Factor (SDSF), a fatty acid. (Vílchez *et al*. 2010). These molecules do not act on the yeast form, indicating a potential propensity for commensal co-existence.

The host responds to, and influences, some fungal-bacterial interactions. This has been observed for interactions between *C. albicans* and *Pseudomonas aeruginosa*, which engage in an interactive molecular dialogue that leads to mutual enhancement of their virulence (Chen *et al*. 2014) (Fig. 7). *C. albicans* produces ethanol, which favours the production of more toxic classes of phenazines by *P. aeruginosa*, such as pyocyanin, phenazine methosulfate and phenazine-1-carboxylate. As well as inhibiting filamentation and biofilm formation, these phenazines induce *C. albicans* to produce more ethanol by compromising mitochondrial functionality (Morales *et al*. 2013; Lindsay *et al*. 2014). Ethanol reduces the ability of macrophages to clear *P. aeruginosa* (Greenberg *et al*. 1999), while phenazines cause damage to respiratory epithelial tissues (Rada and Leto 2013). The mammalian host contributes actively to this interplay by responding to phenazines *via* the Aryl hydrocarbon Receptor (AhR) to enhance antimicrobial defences (Moura-Alves *et al*. 2014). Significantly, there is a strong association between ethanol production by *Candida* and the development of oral cancer (Alnuaimi *et al*. 2016).

The mammalian AhR is a multi-class receptor that modulates disease resistance (by activating IL-17A/IL-22 production) and disease tolerance (*via* TGF β activated Treg cell differentiation) (Cheng *et al*. 2010; Zelante *et al*. 2013; Bessede *et al*. 2014) (see *Adaptive immunity*). AhR functions within the indoleamine 2,3-dioxygenase 1 (IDO1)-catalysed pathway that converts tryptophan to L-kynurenine (Bessede *et al*. 2014). This pathway plays a dual role as microbial commensals use it to enhance host resistance, while pathogenic populations exploit it to dampen immune responses (Cheng *et al*. 2010; Zelante *et al*. 2013) (Fig. 7). Lactobacilli can switch their usage of carbon sources from sugar to tryptophan and utilise this pathway to initiate strain- and location-specific host effects that protect against *C. albicans* infection (Zelante *et al*. 2013). *L. reuteri* produces indole-3-aldehyde (3-IAld), which binds AhR and triggers the production of IL-22 in the gut. Meanwhile, *L. acidophilus* utilises the AhR/IL-22 axis against *C. albicans* in the vagina. However, *C. albicans* is able to switch tryptophan degradation mechanisms from L-kynurenine to 5-hydroxyltryptophan, which inhibits IL-17 production and impairs the host response against infections (Cheng *et al*. 2010). These examples illustrate the complexity of interactions between the fungus, the host and the microbiota.

### Impact of a changing microbiota on the Fungus-Host-Microbiota interplay

**Figure 8.**
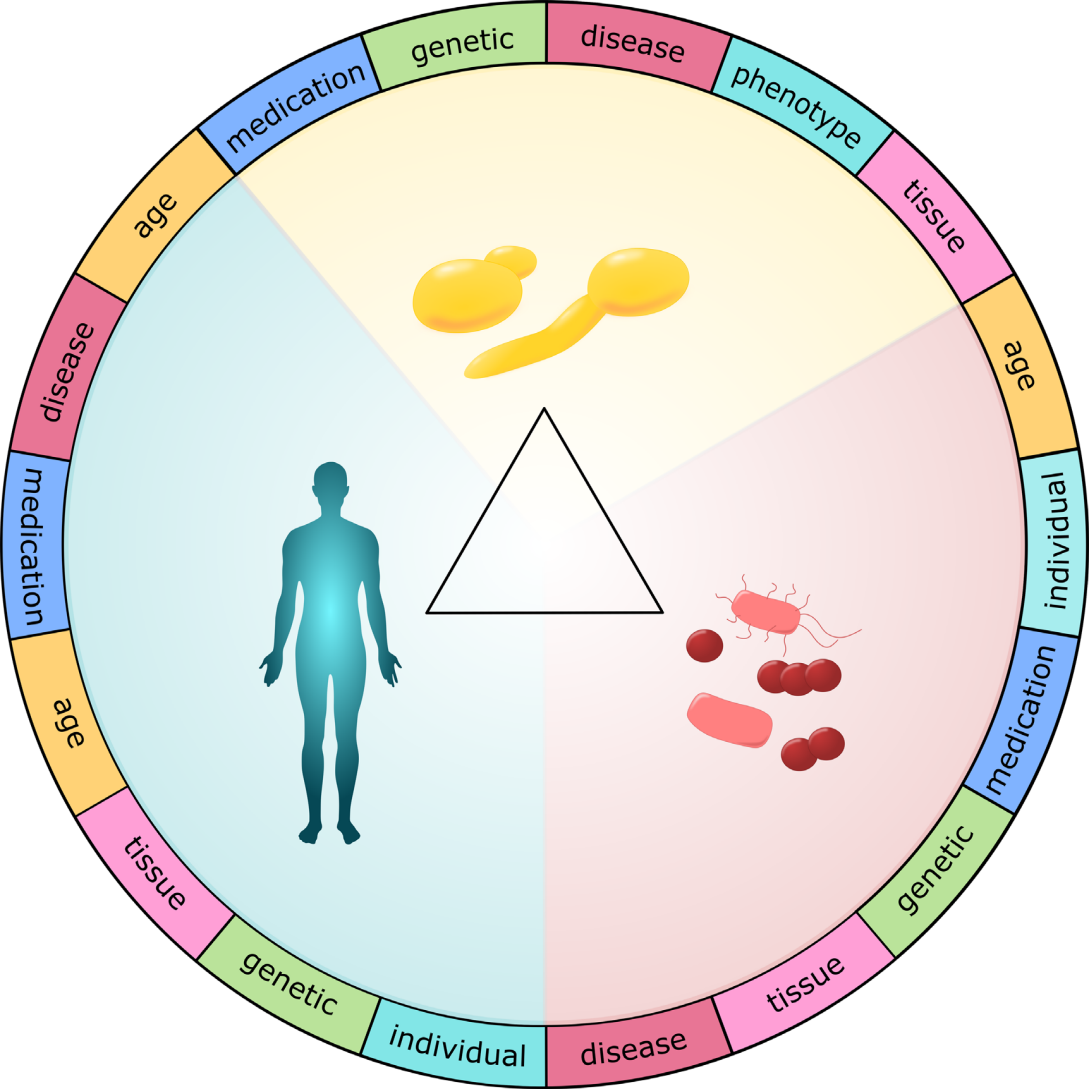
**The complexity of fungus*-*host-microbiota interactions is dramatically increased by variability between *C. albicans* clinical isolates, between individuals, and in their microbiotas**. Fungal variability arises through significant genetic and phenotypic variation between clinical isolates of *C. albicans*. The immune-competence of individuals varies significantly depending on their genetics, age and lifestyle. Furthermore, the microbiotas of the GI tract, oral cavity and vagina can each vary dramatically, depending on the age and health of the individual, and their diet, possible medications and life circumstances. Therefore, variation in each of the three elements of the fungus*-*host-microbiota interplay strongly influences the susceptibility of an individual to *C. albicans* infection as well as the outcome of that infection. See text.

The host microbiota contributes to anti-*Candida* defences through colonisation resistance. Consequently, perturbations of the healthy oral, gut and vaginal microbiota can predispose the host to *C. albicans* infection (see *The Microbiota*) (Fig. 8). However, *C. albicans* is not a passive player in these interactions (see *The Fungus*). For example, the fungus actively promotes oral microbiota perturbations under conditions of immunosuppression by increasing the prevalence of enterococci, which negatively impacts the integrity of the epithelial barrier and enhances *C. albicans* invasion (Bertolini *et al*. 2019). The complexity of interactions between the fungus, host and microbiota are also evident in the vagina, where high oestrogen levels promote *Lactobacillus* spp. colonisation, and affect *C. albicans* morphogenesis, thereby influencing the risk of *C. albicans* colonisation (see *Vaginal mucosa*). Colonisation resistance against *C. albicans* arises though a number of synergistic mechanisms, many of which target fungal virulence traits or modulate the host's response.

Several members of the gut microbiota that contribute to colonisation resistance against *C. albicans* produce short chain fatty acids (SCFAs). Antibiotic treatment leads to a reduction in colonic SCFAs and, consequently, an increase in the susceptibility of mice to *C. albicans* infection (Noverr and Huffnagle 2004; Guinan *et al*. 2019). Butyrate, in particular, has a profound impact on *C. albicans* growth, biofilm and hypha formation (Nguyen *et al*. 2011; Guinan *et al*. 2019). In the colon, such effects are unlikely to be mediated by weak acid stress (Ramsdale *et al*. 2008) given that the ambient pH of this niche is above the pKa for this SCFA. Rather, butyrate perturbs iron homeostasis (Cottier *et al*. 2015) and inhibits the metabolic activity of the fungus (Nguyen *et al*. 2011; Guinan *et al*. 2019) *via* Mig1 regulation of *HGT16*, which encodes a putative glucose transporter (Cottier *et al*. 2017). *In vitro* studies have demonstrated that SCFAs impair *C. albicans* morphogenesis and biofilm formation, in part by reducing the ambient pH (Noverr and Huffnagle 2004; Nguyen *et al*. 2011; Guinan *et al*. 2019). Similarly, lactic acid, generated by lactobacilli, maintains an acidic vaginal pH that inhibits *C. albicans* morphogenesis (Köhler, Assefa and Reid 2012). Although *Lactobacillus* spp. also produce hydrogen peroxide, it is believed that lactic acid is the main basis for anti-*Candida* activity in the vagina (Strus *et al*. 2005; Köhler, Assefa and Reid 2012).

Both hypha and biofilm formation promote the pathogenicity of *C. albicans*. The formation of *C. albicans* biofilms depends upon the yeast-to-hypha transition, and is a significant clinical challenge (see *Virulence factors*). Some members of the microbiota have been shown to hinder *C. albicans* morphogenesis and biofilm formation *via* secreted enzymes (Allonsius *et al*. 2019) or other products (Jarosz *et al*. 2009; Vílchez *et al*. 2010; James *et al*. 2016; Oliveira *et al*. 2016; Hager *et al*. 2019; Jang *et al*. 2019). Nevertheless, *C. albicans* can form polymicrobial biofilms with some members of the oral and gut microbiota, which display elevated drug and host resistance and can strongly influence clinical outcomes (Harriott and Noverr 2011; Fox *et al*. 2014; Cavalcanti *et al*. 2015).

Competition for adhesion sites and nutrients, especially glucose, by members of the microbiota also contributes to colonisation resistance against *C. albicans* in the gut, vagina and oral cavity (Boris *et al*. 1998; Basson 2000; Graf *et al*. 2019). *L. rhamnosus* GG is a common gut and oral isolate (Ahrné *et al*. 1998) that has been shown to prevent *C. albicans*-induced damage and invasion through both nutrient depletion and blocking of adhesion sites (Mailänder-Sánchez *et al*. 2017). *L. rhamnosus* can also reduce *C. albicans* proliferation and induce shedding from the epithelial barrier, thereby preventing invasion of the fungus into the tissue (Graf *et al*. 2019). Interactions between the microbiota, including microbial-associated factors, and the host immune system help to regulate *C. albicans* and prevent dissemination.

The microbiota also influences the colonisation of *C. albicans* indirectly by influencing host functionality. Macrophages exposed to microbiota-produced butyrate are more efficient at phagocytosing *C. albicans* cells, and they produce increased levels of nitric oxide, which enhances eradication of the pathogen (Nguyen *et al*. 2011). In response to butyrate generated by the microbiota, colon epithelial cells express the AMP, LL-37 (Schauber *et al*. 2003), which exerts candidacidal effects (see *Innate antifungal responses*). These host cells also activate LL-37 production in response to microbiota-induced hypoxia *via* HIF-1α (Hypoxia Induced Factor 1α) (Fan *et al*. 2015). *Blautia producta* and *Bacteroides thetaiotaomicron*, both common members of the human gut microbiota, promote colonisation resistance and eliminate *C. albicans* by stimulating LL-37 production in mice (Fan *et al*. 2015). Colonisation resistance against *C. albicans* is also provided by IL-22, which is produced by the host and induced by lactobacilli (Zelante *et al*. 2013). In addition, *L. rhamnosus* GG modulates the inflammatory response of epithelial cells by reducing IL-1α and GM-CSF production (Mailänder-Sánchez *et al*. 2017). By limiting the *C. albicans*-induced proinflammatory response of vaginal cells (with the exception of IL-1α and IL-1β), lactobacilli can alleviate symptomatic vulvovaginal candidiasis while sustaining an antifungal immune response (Wagner and Johnson 2012). Similarly, *L. crispatus* reduces epithelial TLR2/4 expression and IL-8 levels in the presence of *C. albicans*, but maintains antifungal activity by increasing β-defensin production (Rizzo, Losacco and Carratelli 2013). Clearly, the changes in the microbiota strongly influence the iterative interactions between fungus, host and microbiota. Specific probiotic bacteria, including *Bifidobacterium breve, L. rhamnosus*, and *Lactobacillus casei* can also modulate specific PRR ligand- and *C. albicans-*induced cytokine responses (Plantinga, van Bergenhenegouwen *et al*. 2012).

### Impact of patient variability upon the *Fungus-Host-Microbiota* interplay

The nature of an individual affects the types and outcomes of fungal-microbiota interactions directly and indirectly by: (i) genetic determinants that influence immune responses; (ii) personal environment and lifestyle, which affect the microbiota and (iii) iatrogenic interventions that target the microbiota or host response (Fig. 8). As outlined above, the microbiota is critical for colonisation resistance, leading to host protection, but on the other hand, certain combinations of opportunistic pathogens synergise to promote infection (see *Synergistic and antagonistic interactions between kingdoms*). It is well known that diet strongly influences the human gut microbiota (David, Maurice *et al*. 2014; Jeziorek, Frej-Mądrzak and Choroszy-Król 2019). Fundamental differences in diet, and possibly also exposure to microbes, are the most likely reason for the observed differences in *C. albicans* colonisation rates between industrialised and rural countries, which can differ by over 10-fold (Angebault *et al*. 2013) (see *Gastrointestinal (GI) tract*). Antibiotic treatments are probably the most common iatrogenic intervention that directly affects the microbiota, and one of the main predisposing factors to candidiasis in general. More specific iatrogenic factors include oral contraceptives and dental prostheses, which alter the local mucosal environment and thereby promote vaginal and oral candidiasis, respectively (Mothibe and Patel 2017; Jacob *et al*. 2018).

A healthy immune system is crucial for protection against fungal infections (see *The Host*). Individuals vary in their susceptibility to *C. albicans* infection because of genetic differences that affect susceptibility, and the existence of coexisting morbidities in some individuals. Genetic variations in key receptors or molecular effectors have been shown to increase the risk of *Candida* infections (see *Variability amongst individuals*). For instance, monogenic primary immunodeficiency syndromes highly predispose an individual to haematogenously disseminated candidiasis and mucosal candidiasis (e.g. OPC, skin, nails). However, the genetic mutations defined to date do not explain the observed variation in susceptibility to candidiasis within not-at-risk subjects. Phenotypic variation occurs also in healthy individuals. For instance, if primary immune cells from healthy immunocompetent individuals are challenged with *C. albicans in vitro*, different outcomes are observed due to variation in their immune cell populations (Misme-Aucouturier *et al*. 2017). This can arise through genetic variation at the *CR1* locus, which encodes a master regulator of *C. albicans*-specific immune responses (Piasecka *et al*. 2018). Thus, inter-individual variability in innate and adaptive responses against *Candida* spp. are likely to influence the degree of host-mediated damage during infection (Alvarez-Rueda *et al*. 2020). Consequently, understanding the basis of subject-to-subject diversity, and how this affects *Candida* pathogenicity, is likely to prove important for prevention and therapeutic strategies.

Comorbidities and treatment of other diseases can also affect a patient's susceptibility to *C. albicans* infection (see *C. albicans commensalism and pathogenicity*). Uncontrolled diabetes, for example, favours both bacterial and yeast infections due to metabolic alterations and impaired antimicrobial activity (Rodrigues, Rodrigues and Henriques 2019). Admission to an ICU, medical surgery, hematopoietic stem cell transplantation, and the use of external devices are independent risk factors for candidaemia and, together with the duration of hospitalisation, affect the mortality rates for candidiasis infections (Ortega *et al*. 2005; Das *et al*. 2011; Falcone *et al*. 2017; Poissy *et al*. 2020). These patients are commonly immunocompromised, either as a result of their primary disease, or through treatment. For example, OPC is a hallmark of HIV positive individuals and cancer patients (Samaranayake 1992; Redding *et al*. 1999). Moreover, as mentioned, dysregulated innate immunity is associated with failure to clear *Candida* spp., for example in neutropenic patients or neutrophil-related disorders (Nucci *et al*. 1997; Desai and Lionakis 2018). Glucocorticoids (Fan *et al*. 2012) and chemotherapy (Teoh and Pavelka 2016) weaken the host defence and increase the risk for invasive candidiasis.

### Impact of fungal variability on the *Fungus-Host-Microbiota* interplay

Variability in *C. albicans-*host relationships is driven by the fungus as well as the host and its microbiota (Fig. 8). Clinical isolates of *C. albicans* display a high degree of genetic and phenotypic diversity (see *Candida albicans epidemiology and variability*). This fungal diversity can be observed at the genetic level (Tavanti *et al*. 2006; Cavalieri *et al*. 2017; Schönherr *et al*. 2017) as well as the transcriptional level (Thewes *et al*. 2008). The variation impacts multifarious aspects of *C. albicans* biology, such as stress and nutrient responses, and virulence properties such as morphogenesis, adhesion and invasion, that consequently, influence initial host-pathogen interactions, as well as colonisation and infection (see *The Fungus*). Therefore, it is not surprising that fungal variation affects the fitness of a given *C. albicans* strain in the host, and also disease outcome (Thewes *et al*. 2007, 2008; Cavalieri *et al*. 2017; Schönherr *et al*. 2017; Kirchner *et al*. 2019).

In principle, *C. albicans* strains can be classified on the basis of their virulence, rather than their epidemiological relationship. Comparative studies of various *C. albicans* isolates have identified genes whose expression or lack of expression strongly influences the virulence potential of these strains. Examples include *EFG1*, encoding a key transcription factor involved in morphogenesis (Hirakawa *et al*. 2015), and *DFG16*, encoding a pH sensor (Thewes *et al*. 2007). Strains displaying reduced expression of *EFG1* or *DFG16* display reduced virulence in mouse models of systemic infection (Thewes *et al*. 2007; Hirakawa *et al*. 2015). Even the development of hemizygosity at the *EFG1* locus is sufficient to promote commensalism, rather than pathogenicity, in *C. albicans* (Liang *et al*. 2019).

A number of studies have highlighted the significance of variabilities between *C. albicans* isolates. The three *C. albicans* isolates, SC5314, 101 and ATCC10231 are all able to form hyphae. Nevertheless, SC5314 displays enhanced tissue invasion compared to ATCC10231 and 101 (Thewes *et al*. 2007; Schönherr *et al*. 2017), resulting in higher virulence. The strain SC5314 triggers rapid neutrophil recruitment and the production of proinflammatory cytokines leading to fungal clearance of the oral mucosa. In contrast, strains with lower virulence induce slower and weaker immune responses, which lead to fungal persistence (Schönherr *et al*. 2017). Similar results have been observed in a murine model of vaginal colonisation, where a less immunostimulatory *C. albicans* strain is able to persist over five weeks, in contrast to SC5314, which is cleared by week three (Rahman *et al*. 2007). The genetic background of *C. albicans* also influences survival in the phagosome (Tavanti *et al*. 2006; Cavalieri *et al*. 2017), the relative importance of specific PRRs for fungal clearance *in vivo* (Marakalala *et al*. 2013) and even the polarisation of the immune response (Cavalieri *et al*. 2017). Clearly, the intraspecies diversity of *C. albicans* has major consequences for the outcome of host-pathogen interactions.

## NEW CHALLENGES

### Elaborating the complexity of the microbiota

#### Meta-omics

The ability to define the complexity of relevant microbiotas rapidly and accurately represents a major challenge. It is vital that we address this challenge to establish phenotypic associations with specific members of the microbial community. Meta-omics, which refers to culture-independent functional and sequence-based analysis of the collective microbial genomes, transcriptomes, proteomes or metabolomes in a given sample (Handelsman *et al*. 1998; Riesenfeld, Schloss and Handelsman 2004), includes a powerful set of approaches to achieve this.

To date, DNA sequence-based studies have often been based on the analysis of amplicons generated from the microbial community with specific primers that are typically targeted towards bacterial 16S ribosomal RNA genes, and fungal 18S rRNA genes or internal transcribed spacer (ITS) regions. These approaches can provide comprehensive overviews of microbiota compositions, without directly assessing functional capabilities. However, the continuing development of DNA sequencing and genome analysis bioinformatics tools is permitting more widespread use of full shotgun metagenomics instead. This approach does not rely on amplification of marker genes since the extracted DNA is sequenced directly. The approach is more expensive and computationally intensive than marker gene sequencing because it requires sequencing to be carried out at a much higher depth. However, it has the advantages of avoiding biases associated with the amplification step, and generating information on both the function and composition of microbiotas, thereby providing information at much greater resolution (Walker *et al*. 2014; Almeida *et al*. 2019). For example, a recent metagenomic analysis of the human gut microbiota from over eleven thousand individuals identified 1952 candidate bacterial species that have not yet been cultured and increased the known phylogenetic diversity of the gut microbiota almost three-fold (Almeida *et al*. 2019). Furthermore, these new genomes were estimated to encode hundreds of new biosynthetic gene clusters, revealing valuable clues about the potential functionalities of these novel candidate species.

In principle, full shotgun metagenomics can also be applied to mycobiota studies, but further advances are essential before fungal metagenomes can be analyzed more accurately. In particular, the lack of non-redundant and comprehensive fungal databases presents one of the most significant limitations. The accuracy of sequence classification depends fundamentally on the quality of the reference database, and, due to the large number of microbial species that have not yet been identified or genome sequenced, the existing databases are incomplete. This problem is gradually being lessened however, by the continual addition of genomes from newly isolated species (see *Culturomics*) and metagenome-assembled genomes (MAGs), which can provide reasonably accurate draft genomes for uncultured organisms.

Metagenomics can also be complemented by other -omic approaches to increase the power of meta-analyses. For example, metagenomics is being combined with meta-transcriptomic [i.e. the combined transcriptomes of the microbial community as a whole (Martinez *et al*. 2016; Franzosa *et al*. 2018)], proteomic (Van Belkum *et al*. 2018; Zhou *et al*. 2019), and metabolomic data sets (Smirnov *et al*. 2016; Yachida *et al*. 2019). Major software challenges must be addressed to improve the efficiency with which these different data sets can be integrated. For example, linking a metabolic gene to its transcript is relatively straightforward, but linking these to the corresponding enzyme and metabolic reaction is less so. Nevertheless, studies such as these are enhancing the associations between the composition of the gut microbiota and disease state for numerous conditions, including cancer, diabetes and inflammatory bowel diseases (Erickson *et al*. 2012; Smirnov *et al*. 2016; Zhang *et al*. 2018; Yachida *et al*. 2019; Zhou *et al*. 2019). These technologies have the potential to revolutionise our understanding of fungus-host-microbiota interactions and, as a result, our ability to develop personalised therapeutic strategies for individuals at risk of life-threatening fungal infections.

#### Culturomics

In the early 2010s, the use of high throughput culturing coupled to MALDI-ToF mass spectrometry (MS) revolutionised clinical microbiology (Seng *et al*. 2009; Bizzini *et al*. 2010; van Veen, Claas and Kuijper 2010). This has since been termed ‘*culturomics’* (Lagier *et al*. 2012). In brief, culturomics can identify atypical bacteria by combining multiple culture conditions (Beijerinck 1901; Weinstein 1996) with MALDI-ToF MS and 16S rRNA gene sequencing. The pioneering study used 212 culture conditions (Lagier *et al*. 2012), which was subsequently reduced to 18 conditions (Lagier *et al*. 2015) and recently the overall workload has been further reduced (Chang *et al*. 2019). Culturomics permits the identification of microbial minorities present at concentrations lower than 1e + 05 CFU/mL, which can encompass up to 65% of bacterial species in a given sample (Lagier *et al*. 2012). This not only enables a better description of the bacterial diversity (Dubourg *et al*. 2014), but also provides viable microbes for downstream analysis. Downstream characterisations of the new species can include pathogenic potential, metabolic functionality and interactions with other residents of the microbiota studied.

Newly identified species whose genomes have been sequenced can be used to identify previously found, yet unidentified, operational taxonomic units (OTUs), thus filling gaps in sequence-based analyses (Rinke *et al*. 2013; Lagier *et al*. 2016). Between 2015, when 2172 different species cultured from different human body sites were reported (Hugon *et al*. 2015), and 2018, 288 new species were isolated by culturomics (Bilen *et al*. 2018). Therefore, culturomics and metagenomics are complementary techniques, with an overlap as small as 15% of detected species in a given sample (Lagier et al. 2012, 2015; Pfleiderer *et al*. 2013; Dubourg *et al*. 2014; Mailhe *et al*. 2018).

### Models for the experimental dissection of *Fungus-Host-Microbiota* interactions

Model experimental systems are essential for the detailed mechanistic dissection of disease establishment and progression in humans. Models of fungal infection can simulate the process with some degree of accuracy, but they never recapitulate human infections perfectly. Therefore, selecting an appropriate model is a crucial step that requires consideration of many parameters, such as similarity to the human situation, cost, workload, throughput and ethical concerns (Maccallum 2012; Brunke *et al*. 2015; Poupet *et al*. 2020). It is important to reconsider the relevance of a model to the human condition, and to clearly define the limitations of the model as well as the aspects of the human infection that are recapitulated by the model.

#### Mucosa simulating models

Rodents, particularly mouse models, have been used extensively to study vaginal and oral candidiasis (Rahman *et al*. 2007; Solis and Filler 2012; Cassone and Sobel 2016), as well as systemic candidiasis (MacCallum and Odds 2005; Szabo and MacCallum 2011; Brunke *et al*. 2015), allowing investigators to better understand the pathogenicity of *C. albicans*. However, there are significant differences between the immune systems of mice and humans (Mestas and Hughes 2004). Also, most laboratory mice are not naturally colonised by *C. albicans* and therefore do not develop a primed immunity to this opportunistic pathogen (Cassone and Sobel 2016). Moreover, the GI microbiota established in laboratory rodents generally mediates colonisation resistance against *C. albicans*. Thus, antibiotic treatment is required for prolonged high-level colonisation of the murine gut by *C. albicans* (Conti, Huppler *et al*. 2014). Oral models generally focus on infection, rather than colonisation, and immunosuppressive treatment is usually required to induce OPC (Solis and Filler 2012). Also, to study VVC in mice, oestrogen treatment is necessary to facilitate vaginal colonisation by *C. albicans* (Cassone and Sobel 2016).

Alternative *in vivo* mammalian models have been used to study Fungus-Host-Microbiota interactions, such as piglets and non-human primates, which are naturally colonised by *C. albicans*. However, these are cost- and labour-intensive and present ethical challenges (Steele, Ratterree and Fidel 1999; Cassone and Sobel 2016; M Jaeger *et al*. 2019). Under these circumstances, model hosts of lower phylogenetic or ontogenetic stage can provide alternative platforms to study *C. albicans* infections. Non-mammalian models that have been exploited to study *C. albicans* pathogenesis include a chorio-allantoic membrane chicken embryo model, zebrafish, nematodes and insects (Brennan *et al*. 2002; Gow *et al*. 2003; Jacobsen *et al*. 2011; Tobin, May and Wheeler 2012; Brunke *et al*. 2015).

*In vitro* cell culture systems also provide useful models of *C. albicans* infection. These are less expensive, provide higher throughput and present fewer ethical concerns compared to *in vivo* models. For example, static cell culture models that mimic *C. albicans* interactions with intestinal epithelial cells have been used to dissect processes involved in translocation through intestinal barriers (Allert *et al*. 2018). Also, *in vitro* circulatory *C. albicans*-endothelium interaction models have been used to study endothelial adhesion events under conditions of physiological blood pressure (Wilson and Hube 2010). Reconstituted Human Epithelium (RHE) uses Transwell® technology to form polarised epithelia and allows easy access to the apical and basolateral compartments for infection studies (Schaller *et al*. 2006). Such models closely recapitulate the histology of normal vaginal and oral mucosae and relevant aspects of innate immune responses (Schaller *et al*. 2005; Yadev *et al*. 2011) and can mimic epithelial interactions with phagocytes (Weindl *et al*. 2007). However, RHE models do have limitations, such as the lack of supporting cell types, the absence of mucins, non-constant desquamation, and the overgrowth of microbes due to static conditions (Tabatabaei, Moharamzadeh and Tayebi 2020). These limitations need to be addressed to gain accurate views of fungal infection.

Recently, human cell lines were incorporated into an oral-mucosa-on-a-chip model to study host-microbiota interactions (Rahimi *et al*. 2018). Also, oral mucosa organoids, which recapitulate the original tissue genetically, histologically and functionally, have been established (Driehuis *et al*. 2019). In principle, these organoids could be developed to integrate, for example, supporting cells and saliva, to further enhance their relevance to the natural oral mucosa. Organ-on-a-chip models of vaginal infection are under development (https://gtr.ukri.org/projects?ref=studentship-1818626; https://ncats.nih.gov/tissuechip/chip/female). Ideally, these models would include iron restriction and hypoxia as these conditions are known to influence the behaviour of *C. albicans* (Moosa *et al*. 2004; Sosinska *et al*. 2008; Rastogi *et al*. 2016; Pradhan et al. 2018, 2019). In the future, organ-on-a-chip models of oral and vaginal infection will exploit microfluidic platforms to combine patient-derived primary cells and microbes to represent donor variability and permit the development of predictive and potentially personalized infection models.

#### Gut simulating models

Models are also critical for the experimental dissection of host-microbiota interactions in the GI tract. These include organoid models as well as specialised fermentation systems (Fehlbaum *et al*. 2015; Park *et al*. 2017; Bein *et al*. 2018; Pearce *et al*. 2018; Pham *et al*. 2019), but the co-culturing of human and microbial cells remains a technical challenge. *L. rhamnosus* has been shown recently to modulate *C. albicans* pathogenicity in a commensal-like co-culture model (Graf *et al*. 2019). A similar model, involving co-culture of intestinal epithelial cells and M-cells, revealed that *C. albicans* translocate preferentially through the M-cell (Albac *et al*. 2016). These models are high-throughput, cost-efficient and able to recapitulate epithelial cell diversity by co-culturing different epithelial cell types. Nevertheless, such models do not provide the complex tissue architecture of the intestinal epithelium *in vivo* and, due to the static conditions, they only offer a short assay window before rapid microbial overgrowth occurs (Albac *et al*. 2016; Park *et al*. 2017; Pearce *et al*. 2018; Graf *et al*. 2019).

On-chip technologies permit the culture of human cells under perfusion, enabling their differentiation into a polarised columnar epithelium (Hyun Jung Kim *et al*. 2016; Trietsch *et al*. 2017). This has been extended to develop an immunocompetent intestine-on-a-chip model using caco-2 epithelial cells, endothelial cells and peripheral blood mononuclear cells (PBMCs) to study the interaction between *C. albicans* and probiotic *L. rhamnosus* (Maurer *et al*. 2019). Although this model already provides three-dimensional structures that resemble organotypic microanatomical structures and mimic microphysiological niches of the human intestine, further improvements to increase mucosa cellular diversity and mucus production are possible (Pan *et al*. 2015; Pearce *et al*. 2018). In the future, long-term cultures of patient-derived intestinal organoids may be feasible, which opens new avenues for the development of gut models that are even more physiologically relevant (Sato *et al*. 2009; Mottawea *et al*. 2019). Patient-derived ileal organoids and faecal samples have been used to culture a complex microbiota in an anaerobic gut-on-a-chip model for up to 5 days (Jalili-Firoozinezhad *et al*. 2019). This type of model is important because it permits the analysis of donor variability and potentially allows the development of personalised therapies.

Fermentation-based models provide powerful *in vitro* tools that permit the dissection of microbial processes in the human GI tract. Static batch fermentations with faecal inocula are the simplest and most frequently used models (Walker *et al*. 2005). These have provided a powerful first approach to study bacterial-fungal interactions and to screen novel therapeutics, but they do not recapitulate the richness of GI compartments (Hillman *et al*. 2017). Therefore, multicompartmental models have been developed (Guerra *et al*. 2012; Venema and van den Abbeele 2013). These often contain three-stage culture reactors (Gibson, Cummings and Macfarlane 1988) that can reproduce differences between proximal (acidic, carbohydrate-rich) and distal colonic regions (neutral, carbohydrate-depleted). The multicompartmental M-SHIME system is a powerful tool that permits the analysis of complex, rich and relatively stable microbial communities within GI compartments from the stomach to descending colon (Van de Wiele *et al*. 2015; Molly, Vande Woestyne and Verstraete 1993). This model has been used to study bacterial-bacterial interactions and the impact of diet and drugs on these interactions (Sivieri *et al*. 2014; Van den Abbeele *et al*. 2016; Marzorati *et al*. 2017; Rivière *et al*. 2018; Lambrecht *et al*. 2019).

Despite their power, these fermentation systems have rarely been used to examine fungal- bacterial interactions. In two studies, *C. albicans* colonisation and outgrowth was shown to be strongly correlated with antibiotic treatment, but mitigated by *L. plantarum* (Payne *et al*. 2003; Wynne *et al*. 2004). However, more recent work has shown that it is important to include the mucus layer to properly simulate the human gut environment *in vitro* (Van den Abbeele, Van de Wiele *et al*. 2011; Van den Abbeele, Gérard *et al*. 2011; Van den Abbeele *et al*. 2013). Indeed, the presence of mucus influenced interactions between the yeast *Saccharomyces boulardii* and *L. rhamnosus* GG and their ability to limit the outgrowth of toxigenic *E. coli* (Moens *et al*. 2019). Therefore, a M-SHIME-based system that includes a mucus-rich environment (Van den Abbeele *et al*. 2012) would seem most appropriate for the dissection of *C. albicans-*microbiota interactions in the GI tract.

## SUMMARY AND OUTLOOK

To summarise, it is clear that the interactions between *C. albicans*, the human host, and the local microbiota have a major impact upon the likelihood of mucosal and systemic infections and the severity of these infections. It is also apparent that these fungus-host-microbiota interactions are dynamic, iterative and enormously complex (Fig. 8). This immense complexity is increased further by the genetic and phenotypic variation within the species of *C. albicans*, and by numerous factors that contribute to the variability of individuals and their microbiotas. Yet this complexity must be addressed and defined if the research community is to develop: (i) sensitive and accurate diagnostics capable of distinguishing *C. albicans* infection from commensalism, and at an early stage when the infection is more amenable to therapy; (ii) novel and efficacious anti-fungal therapies that complement the limited antifungal drugs that are currently available, and that address the problematic emergence of drug resistance and drug resistant species; (iii) tests that quickly establish whether a particular patient is at risk of developing severe candidaemia or recurrent candidiasis and (iv) personalised therapeutic strategies that address the specific make-up and needs of the individual patient.

Despite these challenges, our increased understanding of antifungal immunity and responses is offering potential immunotherapeutic opportunities (De Luca *et al*. 2013; Davidson, Netea and Kullberg 2018). Effective anti-*Candida* vaccines, which have proven elusive for so long, are now in sight (Cassone 2015; De Bernardis *et al*. 2018; Edwards *et al*. 2018). Our deeper comprehension of fungal immune evasion strategies affords the potential to block these phenotypes and thereby enhance the efficacy of natural antifungal immunity mechanisms (Childers *et al*. 2020). Significantly, the dramatic expansion in genomic and phenotypic datasets for clinical isolates of *C. albicans* is providing a much clearer picture of the nature of fungal variability and in-patient evolution (Selmecki, Forche and Berman 2006; Ford *et al*. 2014; Hirakawa *et al*. 2015; Ropars *et al*. 2018; Sitterlé *et al*. 2019). This information is vital because it will reveal ways in which this microevolution might be inhibited or exploited therapeutically. This information will also highlight essential fungal processes that are less prone to variability and hence present better therapeutic targets. Rapid advances in metagenomics and culturomics are highlighting fungal-bacterial associations between the gut microbiota that are likely to yield useful prognostic tools for patients at risk of systemic candidiasis (Yachida *et al*. 2019). Our increased knowledge of local fungus-microbiota interactions is facilitating the development of probiotic therapies to address VVC, OPC and *C. albicans* colonisation of the GI tract (Romeo *et al*. 2011; Hu *et al*. 2013; Morais *et al*. 2017; Vladareanu *et al*. 2018). In the future, the availability of effective probiotics should help to reduce our dependence on antifungal drugs while, at the same time, enhancing antifungal immunity (Ubeda and Pamer 2012).

How relevant are these points to other fungal pathogens of humans, such as *Aspergillus, Cryptococcus, Pneumocystis* and other pathogenic *Candida* species? Pathogenic *Aspergillus* and *Cryptococcus* species are environmental fungi that infect humans *via* the lung. Therefore, while fundamental principles relating to local antifungal immunity, immunotherapy and microbiota-mediated colonisation resistance are clearly of relevance (Armstrong-James *et al*. 2017; Dumas *et al*. 2018; Hernández-Santos *et al*. 2018; Drummond and Lionakis 2019; Maschirow, Suttorp and Opitz 2019; Warris, Bercusson and Armstrong-James 2019), the specific details will differ significantly. *Pneumocystis jirovecii* also infects the lung, but this fungus is an intracellular parasite that is obligately associated with its human host. In this case, the lung microbiota has not been particularly informative in distinguishing infected from uninfected patients (Kehrmann *et al*. 2017). Although other *Candida* pathogens may infect from environmental reservoirs, these species cause similar types of infection to *C. albicans*, and therefore the points raised in this review will be of general relevance to these species. However, some differences in tissue tropism and patient type exist between species (Sullivan *et al*. 1995; Silva *et al*. 2012; Pammi *et al*. 2013), and some species differ in their immune avoidance strategies (Brunke and Hube 2013; Kasper, Seider and Hube 2015). Nevertheless, the importance of the general principles discussed in this *C. albicans-*oriented review cannot be understated, most notably the major impact of variability in the fungus, the individual host, and the local microbiota upon disease severity and outcome (Carvalho *et al*. 2010; Farrer *et al*. 2015; Hube 2015; Ballard *et al*. 2018; Stone *et al*. 2019; Vandeplassche *et al*. 2019).
